# A Comprehensive Analysis of Short Specific Tissue (SST) Proteins, a New Group of Proteins from PF10950 That May Give Rise to Cyclopeptide Alkaloids

**DOI:** 10.3390/plants14071117

**Published:** 2025-04-03

**Authors:** Lucía Albornos, Paula Iriondo, Silvia Rodríguez-Marcos, Patricia Farelo, Guillermo Sobrino-Mengual, Luz María Muñoz-Centeno, Ignacio Martín, Berta Dopico

**Affiliations:** 1 Department of Botany and Plant Physiology, University of Salamanca, Campus Miguel de Unamuno, 37007 Salamanca, Spain; piriondocampo@usal.es (P.I.); silvia.rodriguezmarcos@wur.nl (S.R.-M.); pmfoba@gmail.com (P.F.); guillermo.sobrino@udl.cat (G.S.-M.); luzma@usal.es (L.M.M.-C.); a56562@usal.es (I.M.); 2 Institute for Agribiotechnology Research (CIALE), University of Salamanca, Campus Villamayor-Parque Científico, 37185 Villamayor, Spain

**Keywords:** DUF2775 domain, SST protein, ST protein, root, cyclopeptides alkaloids, PF10950

## Abstract

Proteins of the PF10950 family feature the DUF2775 domain of unknown function. The most studied are specific tissue (ST) proteins with tandem repeats, which are putative precursors of cyclopeptide alkaloids. Here, we study uncharacterised short ST (SST) proteins with the DUFF2775 domain by analysing 194 sequences from 120 species of 39 taxonomic families in silico. SST proteins have a signal peptide and their size and several other characteristics depend on their individual taxonomic family. Sequence analyses revealed that SST proteins contain two well-conserved regions, one resembling the ST repeat, which could constitute the core of cyclopeptide alkaloids. We studied the unique *SST1* gene of *Arabidopsis thaliana*, which is adjacent to and co-expressed with a gene encoding a protein with a BURP domain, associated with cyclopeptide production. The empirical analysis indicated that the *SST1* promoter is mainly activated in the roots, where most of the transcripts accumulate, and that the SST1 protein accumulates in the root vascular cambium. At the cellular level, SST fused to GFP appears in vesicles that co-localise with the endoplasmic reticulum and the vacuole. Thus, SSTs are a new type of PF10950 protein found in core eudicots with two conserved regions that could be involved in root biology.

## 1. Introduction

Proteins belonging to the family PF10950 [1] feature the domain of unknown function DUF2775, whose biological meaning remains elusive. Within this family, the most studied proteins are specific tissue (ST) from *Pisum sativum* L. [2,3]; *Cicer arietinum* L. [4,5,6,7]; and *Medicago truncatula* Gaertn. [8,9,10,11,12]. STs are found exclusively in the plant kingdom and specifically in some core eudicots, mainly *Fabaceae* and *Asteraceae*, but they are absent in *Brassicaceae* such as *Arabidopsis thaliana* (L.) Heynh [8]. ST proteins are encoded by multigenic families, such as the six-member *M. truncatula* family (MtST1 a 6), which is the largest known thus far [8].

Three different regions can be found in ST proteins: an N-terminal signal peptide (SP) that leads the protein toward the secretion pathway, a middle zone containing 80–100 amino acids with some well-conserved residues (called the non-repeat zone), and a region comprising tandem repeats of typically 25 or 26 amino acids containing the characteristic sequence EFEPRPxxxxY. These tandem repeats are highly variable in number [2,3,4,8].

Several functions have been proposed for different ST proteins, such as early fruit morphogenesis [13]; cell elongation [4] and germination [5]; the establishment of biotic interactions like arbuscular mycorrhizal (AM) symbiosis [14]; response to abiotic stress [4,6]; and nitrogen storage [7]. The study of the *M. truncatula* ST family has shed light on the range of roles that these proteins might perform. MtST1 to MtST6 have been classified into three functional groups, with specific roles for each protein. MtST1 is associated with nutritional function in the plant [9], and it is involved in nodule organogenesis in the interaction between *M. truncatula* and *Sinorrhizobium meliloti* [12]. The second functional group includes MtST2 and MtST3, which are related to desiccation tolerance [9]. The last functional group comprises MtST4, MtST5, and MtST6, which have been associated with the establishment of a range of biotic interactions [10]. The involvement of MtST6 in AM symbiosis was reported [15], and MtST6 transcript accumulation throughout the interaction between *M. truncatula* and *Fusarium oxysporum f.* sp. *medicaginis* has been observed [12]. Moreover, the promoter activities of the six *M. truncatula* ST genes throughout flower and fruit development were mainly associated with vascular bundles, especially in pod and seed formation, which could be related to nutrient mobilisation [11].

Recently, it has been reported that the ST proteins could be processed to give rise to a kind of ribosomally synthesised and post-translationally modified peptide (RiPP) [16]. It was proposed that a cyclopeptide alkaloid-type burpitide (CPAB) consisting of five amino acids is produced after each ST repeat. In this process, the phenolic oxygen of the Tyr forms an ether linkage with one of the four preceding amino acids. However, these compounds have not yet been isolated from plants [16,17]. When fully maturated, such cyclopeptide alkaloids (CPAs) can have different biological activities, such as antibacterial, antifungal, antiplasmodial, antimycobacterial, sedative, cytotoxic, and immunostimulant activities, although their biological role in plants is still unknown [18].

Interestingly, in addition to STs, we found another group of proteins named short STs (SSTs) that also contain a DUF2775 domain, which indicates that they belong to PF10950. In contrast to STs, Arabidopsis (*A. thaliana*) does contain an SST. The name of SST was chosen because they lack the tandem repeat domain and, consequently, have a smaller size in comparison to STs. Remarkably, this absence of repeats in SST proteins could create functional differences with STs.

In the present work, we undertake SST characterisation by means of surveying and analysing in silico putative sequences deposited in databases. Also, we focus on the unique SST in *A. thaliana* (SST1), encoded by the At1g49310 gene, and empirically study its promoter activity, transcript accumulation, and subcellular localisation. The fact that *A. thaliana* has a single SST facilitates the functional study of these proteins and might also contribute to the knowledge of ST proteins that share DUF2775 with SST.

## 2. Materials and Methods

### 2.1. Identification, Collection, and Analysis of SST Nucleotide and Amino Acid Sequences

The *SST* genes were identified through a BLAST search against *A. thaliana* on TAIR (NCBI BLAST 2.9.0+) [19], using the amino acid sequence of the non-repeat domain of the MtST3 protein (Medtr3g116430, Gene ID 11422782) without the SP (RNNLGEYWKLFMKDQNMPEEIQGLLSANTKSNLKTLEKKKVFGE) [8]. This search yielded a single Arabidopsis gene (At1g49310) encoding a small protein, which was named AtSST1, abbreviated as SST1. Other results obtained in this search were small areas of much larger proteins, mostly with a BURP domain of unknown function (named after the four founding members, BNM2, USP-like, RD22, and PG1β [20]), which were not considered.

The nucleotide and amino acid sequences encoded by the *SST1* gene were used for further searches, starting with a search against EMBL Release [21], which only retrieved sequences from plants. When sequences from plants were excluded from the searches, no similarities were found. The next step was searching for *SST* genes in the species whose genomes were deposited in the Phytozome v13 database [22,23] using the BLAST search tool on a species-by-species basis working with the nucleotide or amino acid sequence of the SST of the phylogenetically closest species found in the first search. If no *SST* genes were found in any species, a new search was performed using the term DUF2775. The sequences found were visually analysed to discard those with tandem repeats having the EFEPRPxxxxY sequence or similar (characteristic of STs) [8] or BURP domains (PF03181) [1]. Species resulting from crosses, e.g., *Populus maximowiczii* × *nigra*, were not taken into consideration. To visualise the phylogenetic distribution of the species with SST proteins, a tree was constructed with those species that appeared in Phytozome v13 [22,23] using the Common Tree Taxonomy tool [24] according to the NCBI taxonomy. The names of the species without *SST* sequences are indicated in grey, whereas those with *SST* genes are in black. The generated tree was edited using FigTree v1.3.1 [25]. All species tested are collected in List S1.

The selected sequences are listed in Table S1, indicating whether they were obtained from the Phytozome v13 (D), BLASTN (R), or BLASTP (P) databases. The identifiers of each sequence refer to the location in the genome (D), GenBank (R), or Uniprot (P). The sequences were sorted by species (the number was randomly assigned) and grouped by taxonomic families according to the International Plant Names Index (IPNI) [26]. In the file, *Brassicaceae* and *Fabaceae* are listed first, owing to the high number of sequences obtained, followed by the rest of the families ordered alphabetically. Within each family, the species are ordered alphabetically. After an exhaustive analysis of each sequence, non-canonical ones were marked with an asterisk and a number. The meaning of the asterisks is as follows: *1, SP cleavage site does not correspond to the canonical start of the mature protein; *2, not predicted SP; *3, truncated C-terminal; *4, extra N-terminal sequence.

The *SST* genes and their encoded proteins (List S3) underwent various in silico analyses. In the *SST* genes, we analysed the total length of the coding sequence (CDS), the presence and length of an intron near the 5′ end of the sequence, and the size of the first exon for comparison to the *ST* genes [8]. The CDS obtained through BLASTn were translated into proteins [27]. Since the SST proteins were identified using a fragment of the ST proteins, the presence of a putative SP was determined by the SignalP-5.0 tool [28,29], and if undetected or of unusual size, SignalP-6.0 [30,31], TargetP-2.0 [32,33], and DeepLoc-2.0 [34,35]: the latter two were also used to determine its putative subcellular localisation. The presence of transmembrane zones was analysed with the DeepTMHMM program [36,37]. The molecular weight (Mw) and isoelectric point (pI) of the encoded and mature proteins [38], as well as the presence of different motifs [39], were explored. Finally, their possible subcellular localisation was determined using the TargetP-2.0 [33], DeepLoc-2.0 [35], and WoLF PSORT [40] tools.

### 2.2. Alignment of Sequences

Mature proteins (List S3), without an SP, were used to perform the SST alignment. The proteins marked with *1 (not starting with R), *2 (no SP), and *3 (truncated C-terminus) in Table S1, as well as the 18 sequences longer than usual, have been removed as they are putative misannotated sequences (Table S1). Only the sequences marked with *4 that give canonical mature proteins (Table S1) were used. In addition, SST 101 (*Daucus carota* SST1) was excluded due to significant differences in its central area, which affected the sequence alignment. Therefore, in this section we analysed 151 sequences: 52 from *Brassicaceae*, 34 from *Fabaceae*, and 65 sequences from a mixture of other different families (*Other*). List S4 contains the SSTs used in this section in FASTA format obtained in EMBOSS seqret [41,42]. To perform WebLogo alignment, sequence number 109 from *Betula platyphylla* was removed, except in the image represented in Figure S2A. In addition, SST proteins from *M. truncatula* and the non-repeat zone of STs from the same species were aligned, to compare the common region in both types of proteins.

Multiple sequence alignment was performed via the CLUSTAL OMEGA tool [43,44,45]. To make the phylogenetic trees, the output format ClustalW and phylogenetic tree were selected, which is a neighbour-joining tree without distance corrections with the branch length represented as a cladogram. When the guide tree was selected, a cladogram was generated, and the tree was edited using FigTree v1.4.4 [25].

The downloaded file was also opened with the Jalview program [46] and saved as a .fa file. This file was loaded into the WebLogo3 tool [47,48] to create the alignment representation. These representations were made with and without scaling the visible stack widths by the fraction of symbols in the column (i.e., columns with many gaps or unknown residues are narrow). This tool facilitates the visualisation of gaps but hinders the identification of amino acid residues.

### 2.3. Plant Material and Growth Conditions

Seeds of wild-type (WT) and transgenic *A. thaliana* ecotype Columbia-0 (Col-0) plants were sterilised, stratified, germinated, and grown as described previously [9]. To conduct semi-quantitative reverse-transcription polymerase chain reaction (sqRT-PCR) experiments, the roots and aerial parts of 5 and 10 d old seedlings were collected separately, except for etiolated plants. Several organs were collected from adult plants: the entire root; the first pair of leaves (1–1.5 cm in length), the second pair of leaves (0.8–1 cm in length), and the third pair of leaves (0.2–0.5 cm in length); the first internode (the basal part, approximately 1 cm) and the third internode (the apical part, approximately 1 cm); the flower bud and the open flower (in which the siliqua can be seen emerging from the petals); and the young siliqua (growing fruit, when the flower has not yet lost its petals) and the mature siliqua (fully developed but green fruit).

### 2.4. DNA and RNA Extraction, cDNA Synthesis, and sqRT-PCR

Genomic DNA (gDNA) and RNA were obtained as indicated in [9]; for the latter, an extra DNase treatment was performed using the TURBO DNA-free^TM^ kit (Invitrogen, Carlsbad, CA, USA). DNA and RNA quantification was performed using a nanophotometer and the integrity of the RNA was checked by agarose gel electrophoresis. First-strand complementary DNA (cDNA) was synthesised from 1 µg of RNA by priming with oligo dT as described in [9]. PCR, image analysis, and quantification were also performed as indicated in [9]. The quantification of *SST* amplicons was conducted relative to that of *ACTIN2* (Act2, At3g18780) and the band intensity was expressed as the normalised and integrated optical density (nIOD). The primer pairs used were intron-spanning: SST1.F, ACGTTACTTGGTCGTCTTCATCG; SST1.R, TGGTTCAGGCAACGGTTCAT; Act2.F, CACCCTGTTCTTCTTACCGAGGC; Act2.R, TTGGCACAGTGTGAGACACAC. RNA was obtained from three independent biological replicates, two cDNA synthesis reactions of each RNA were performed, and every cDNA template was used for sqPCR at least twice. The means and standard deviations of all the experiments were calculated.

### 2.5. SST1 Cloning and Construction of an Expression Vector for Arabidopsis Transformation

Two different fragments of the *SST1* gene (At1g49310) have been cloned: the promoter (pSST1) 1075 bp upstream from the translation start site, and the complete CDS (*SST1*) without the termination codon (246 bp). These fragments were PCR-amplified (Kapa HiFi HotStart polymerase by Kapa Biosystems, Wilmington, MA, USA) from *A. thaliana* gDNA (pSST1) or from the U22554 clone (SST1) obtained from the Arabidopsis Biological Resource Center (ABRC). The primers used were designed based on the gene sequence obtained from Phytozome v13 and included the attB sites (lower case) of Gateway^TM^ technology (Invitrogen): pSST1 F, ggggacaagtttgtacaaaaaagcaggctgcCAATGGGCTCCTTCAACTAA; SST1 F, ggggacaagtttgtacaaaaaagcaggctgcATGAAGCAACAGCAACGTTAC; pSST1 R, ggggaccactttgtacaagaaagctgggtcGTCTGCTTTAACTTTTGTTTTGAGG; SST1 R, ggggaccactttgtacaagaaagctgggtcTTCATTAGGATTGTGGTAGATG. The amplified products were gel-purified (NucleoSpin^®^ Gel and PCR Clean-up by Macherey-Nagel, Düren, Germany), checked for the correct size, cloned in pDNOR201 and sequenced.

In addition, a 1027 bp fragment including the sequences of attB1, SP, green fluorescent protein (GFP), mature SST1 (mSST1), and attB2 (SP-GFP-mSST1) was chemically synthesised (gblocks^®^ gene fragments, IDT, Coralville, IA, USA), so that once processed in plants, the GFP would be bound to the N-terminal end of the protein. The p35S::SST1, pSST1::GUS, p35S::SST1-GFP, and p35S::SP-GFP-mSST1 gene cassettes were prepared using the Gateway^TM^ system according to the manufacturer’s instructions and inserted in the destination vectors pK7WG2, pKGWFS7, pK7FWG2, and pK7WG2, respectively. The corresponding expression vectors were verified by PCR and subsequently electroporated into the *Agrobacterium tumefaciens* strain C58C1m as indicated in [9].

### 2.6. Arabidopsis thaliana Transformation

The *A. thaliana* ecotype Col-0 was transformed via the Agrobacterium-mediated floral dip method [49]. Several kanamycin-resistant transgenic plants were constructed carrying the transgenes of interest indicated in the previous section. Furthermore, phosphinothricin-resistant transgenic plants carrying marker proteins from different organelles bound to cyan fluorescent protein (CFP) were made. We used the cyan markers for the tonoplast, the endoplasmic reticulum (ER), and the Golgi apparatus obtained from the vectors pFGC5941-vac.cb (V/CFP), pFGC5941-ER.cb (ER/CFP), and pFGC5941-G.cb (G/CFP) [50] provided by the ABRC.

Double-transgenic plants were obtained by crossing selected lines of p35S::SP-GFP-mSST1 or p35S::SST1-GFP with organelle-marked plants and they were selected with kanamycin and phosphinothricin.

Seeds were germinated in the appropriate selection media and T2 plants were used to analyse promoter activities by a GUS assay and subcellular SST localisation by confocal microscopy.

### 2.7. Nicotiana benthamiana Agroinfiltration

For heterologous expression in *N. benthamiana* leaves, the *SST1* CDS, obtained as indicated above, was cloned using Gateway^TM^ cloning technology into the pEAQ-HT-DEST3 vector (provided by Plant Bioscience Ltd., Norwich, UK) [51]. The expression constructs and the GFP-containing pEAQ-GFP-HT vector used as control (also provided by Plant Bioscience Ltd., Norwich, UK) were electroporated into the *A. tumefaciens* strain AGL1, and *N. benthamiana* leaves were agroinfiltrated as described in [52]. Leaves were collected and proteins were extracted as indicated below.

### 2.8. GUS Assay

GUS staining using 5-bromo-4-chloro-3-indolyl-β-D-glucuronide (X-GlcA) (Duchefa, The Netherlands) was performed as described by [5]. GUS activity was assayed in 5 and 10 d old seedlings grown on MS plates (light- and dark-grown seedlings) and in the different organs of 20 to 30 d old plants growing in soil. Images were acquired and experiments were performed as indicated in [9].

### 2.9. Confocal Microscopy

Roots of 20 to 25 d old T2 transgenic plants harbouring the *SST1* CDS fused to GFP (p35S::SST1-GFP or p35S::SP-GFP-mSST1) were observed with a LEICA DMI-6000B microscope equipped with a confocal SP5 system. The cell plasmolysis and cell-wall staining were performed as described by [9].

Similarly, we visualised the roots of the double-transgenic plants with the SST1 fused to GFP and the organelle protein markers tagged with CFP. Images were processed using ImageJ 1.54f/FIJI Software and 3D projections and sections were obtained with Leica LAS X 5.1.0 Software.

### 2.10. Heterologous Expression and Purification of SST1 Recombinant Proteins

The expression and purification of the recombinant mSST1 protein were carried out as previously described [53], using the pET-28a(+) (Novagen, Darmstadt, Germany) expression vector and the *Escherichia coli* BL21 (DE3) strain (Novagen). The mSST1 sequence (171 bp) was PCR-amplified from the pENTR201-SST1 clone previously generated, using NheI.mSST1.F gctagcAGAACAGGAGGAGTTGCAG and HindIII.mSST1.R aagcttTTATTCATTAGGATTGTGGTAGATG primers (the restriction sites are in lower case and the stop codon is underlined). As a control of expression, pET-28a(+) without any insert was used. Briefly, transformed cells were cultured, induced to produce the recombinant protein by adding isopropyl-β-D-galactopyranoside (IPTG; 1 mM), and allowed to produce the protein for 3 h. Once the colony with the highest protein production was selected, a 0–5 h study was conducted to determine the best time for protein production. The inclusion bodies were separated from the soluble fraction using the BugBuster Protein Extraction Reagent (Novagen) according to the manufacturer’s instructions, and the presence of the recombinant protein was determined by SDS–PAGE.

### 2.11. Anti-SST1 Polyclonal Antibody Production and Purification

The most conserved region in SSTs, located in the N-terminal region of the mature protein, was determined by a comparison of 140 SST proteins from different plant species. Subsequently, the sequence of this region in the Arabidopsis SST1 protein (YWKKMMKNEPLPEPIK) was used to generate specific polyclonal antibodies (anti-SST1). The peptide was synthesised by Eurogentec (Seraing, Belgium) which also generated the anti-SST1 antibodies by immunising two New Zealand white rabbits and purifying the immunoglobulins.

### 2.12. Western Blot Experiments

Western blot experiments were performed to test the specificity of anti-SST1. The total proteins and purified inclusion bodies from *E. coli* colonies with and without mSST1 production were assayed, along with protein extracted from Arabidopsis seedlings (WT, p35S::SST, and p35S::SST-GFP transgenic plants) and from *N. benthamiana* leaves (control and agroinfiltrated with the p35S::SST transgene in the pEAQ vector). The proteins from *N. benthamiana* leaves and *A. thaliana* seedlings were extracted according to [54]. Frozen material was homogenised in a RETSCH mixer mill (RETSCH, Haan, Germany) and 200 mg was resuspended in 400 µL of extraction buffer (50 mM Tris/HCl pH 8.0, 100 mM NaCl, 1 mM EDTA, 5 mM β-mercaptoethanol, 0.02% Triton-X-100 (Sigma-Aldrich, St. Louis, MO, USA)).

A Western blot was carried out according to [55]. To separate the proteins in the low mass range, we used a Tricine-SDS-PAGE protocol as described in [56]. The separating gel consisted of two parts, the upper one of 10% and the lower one of 14% acrylamide/bisacrylamide and both with 3% of the crosslinker. The amount of total protein extract per lane was 8 µg and the dilutions of the anti-SST1 antibody and the horseradish peroxidase-conjugated secondary antibody were 1:5000 and 1:75,000, respectively.

### 2.13. Immunocytochemical Labelling of SST1

Immunocytochemical labelling was conducted in the basal zone of radicles of 25 d old seedlings of *A. thaliana*. Sample preparation and incubation with the antibodies were performed as described in [55]. Anti-SST1 and secondary antibody (goat anti-rabbit IgG conjugated with alkaline phosphatase) were applied at a 1:200 and 1:300 dilution, respectively. Once the colour reaction was visualised, sections were dehydrated in a graded ethanol series, dipped in xylene, and mounted in Entellan (Merck, Darmstadt, Germany). Controls were performed using pre-immune serum at 1:200.

## 3. Results

### 3.1. SST Genes Are Exclusive to Core Eudicots

**Figure 1:**
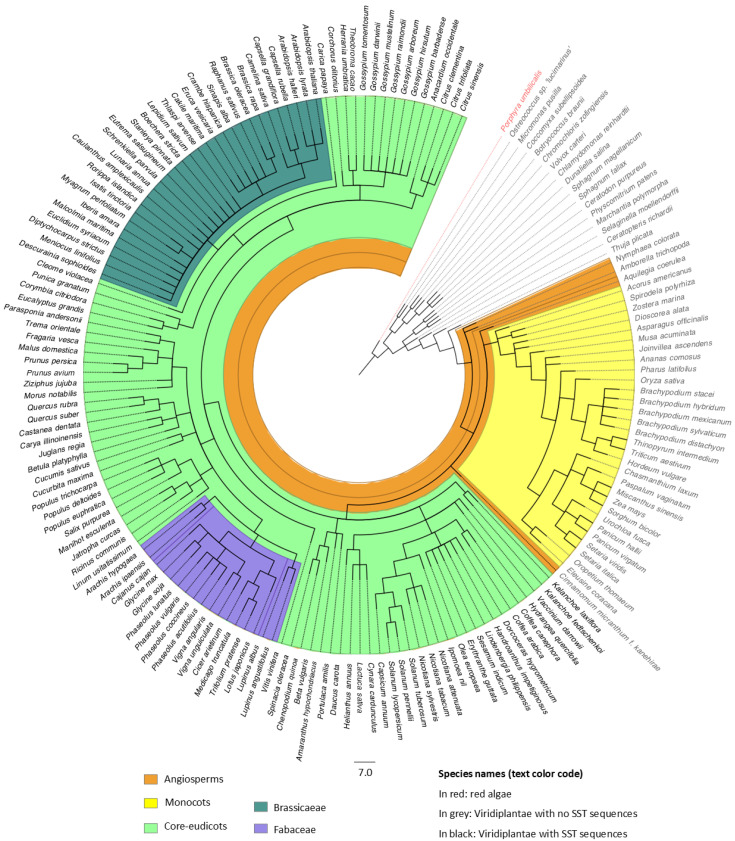
Distribution of SST sequences in the plant kingdom. Taxonomic classification of the species in the Phytozome database showing those in which SST sequences were found (in black), all of them core eudicots. The classification was performed according to the USDA Plants Database. The *Brassicaceae* (green) and *Fabaceae* (purple) families are highlighted. Species without SST sequences are indicated in grey except for red algae, which is shown in red.

The SST proteins were identified by searching databases using the sequence of the non-repeat region of the ST proteins, as detailed in Section 2. We found 120 different species (List S1) encoding SST proteins in their genomes (Table S1), mainly *Brassicaceae* (31 species and 54 sequences) and *Fabaceae* (17 species and 43 sequences) (Figure 1, List S1). In Arabidopsis, SST was encoded by a single gene (Table S1 #8) located on chromosome 1 (At1g49310), while *M. truncatula* had a four-member multigenic family (Medtr3g007880, Medtr3g116395, Medtr4g069770, Medtr5g095980) (#82–85). The largest families were found in *Glycine max* (L.) Merr. and *Glycine soja* Sieb. & Zucc., each having seven SSTs (#61–67 and 68–74, respectively, in Table S1).

Apart from the SSTs in *Brassicaceae* and *Fabaceae*, 97 additional sequences (Table S1) were identified in 72 species from 37 core eudicot families (List S1) (light green in Figure 1). SST sequences were not found in red algae (in red in Figure 1), nor in chlorophytes or bryophytes. Within vascular plants, they were only present in core eudicots (light green in Figure 1) (species where *SST* genes were not found are shown in grey). In fact, among angiosperms (orange in Figure 1), they were absent in Amborellales (*Amborella trichopoda* Baill.) and Nymphaeales (*Nymphaeae colorata* Peter), both classified as the most ancient basal angiosperms; Ranunculales (*Aquilegia coerulea* James), a basal eudicot; monocots (yellow in Figure 1); and Laurales (*Cinnamomum micranthum* Hayata), a less-evolved angiosperm of the Magnoliids group.

### 3.2. SST Gene Structure Determines the Existence of One Intron Close to the 5′ End

**Figure 2:**
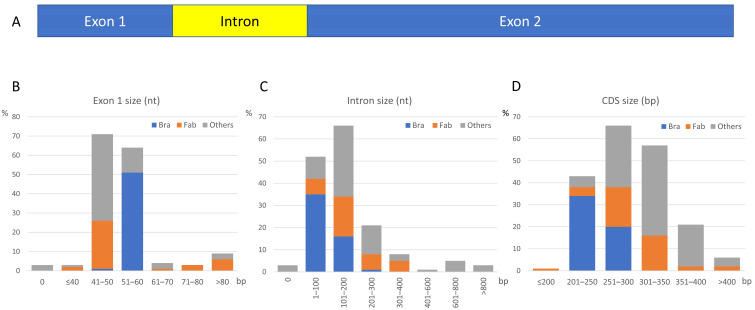
Structure and characteristics of the canonical *SST* gene. (**A**) The *SST* genes have two exons and one intron close to the 5′ end. (**B**) Percentage distribution of exon 1 length in the different families. (**C**) Percentage distribution of intron size in the different families. (**D**) Percentage distribution of the complete CDS size. The numbers indicate the length in bp, and the results have been represented by the three groups of families established in this analysis: *Brassicaceae* (Bra, blue), *Fabaceae* (Fab, orange), and *Other* families (Other, grey).

The *SST* gene structure (Figure 2A) was inferred from the analysis of 157 sequences out of the 194 SSTs found in our searches. We only examined those retrieved from Phytozome v13 [22], in which we could study the whole transcriptional unit.

All of these genes showed a single intron, mostly ranging from 70 to 200 bp (Table S1, Figure 2C), except for three sequences (#119, 120, and 142 in Table S1). The first exon had a typical length between 45 and 60 bp (Figure 2B), while the second one was more variable. Among the *SST* genes examined, the CDS showed a range of sizes depending on the taxonomic family. Therefore, we established three groups for sequence analyses: *Brassicaceae*, *Fabaceae,* and *Other* families. The first exon was 57 bp in 77% of the sequences from *Brassicaceae*, whereas it was 45 bp in most sequences from *Fabaceae* (62%) and from *Other* families (57%) (Table S1, Figure 2B). The complete CDS in most *Brassicaceae* (63%) was 201 to 250 bp, being smaller in comparison with the rest of the groups. In *Fabaceae,* the CDS was 251 to 300 bp (79.1%) (Figure 2D), though we found a CDS shorter than 200 bp (#83) and two longer than 400 bp (#64, 74) (Table S1). Despite the diversity of taxonomic families under the category of *Other* families, several CDSs ranged between 301 and 350 bp (42.3%) and there were four CDSs longer than 400 bp (#108, 109, 162, and 193).

### 3.3. SST Proteins Enter the Secretory Pathway

**Figure 3:**
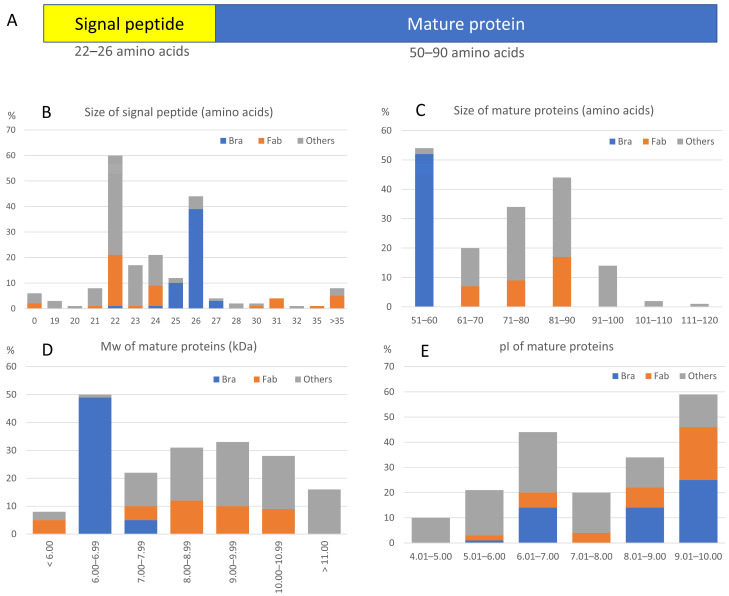
Structure and characteristics of the canonical SST protein. (**A**) Representation of the SST protein domains: the signal peptide and the mature protein, indicating the most frequent sizes. (**B**) Percentage distribution of the signal peptide size in the different families. (**C**) Percentage distribution of mature SST size across families. (**D**) Percentage distribution of the molecular weight (Mw) grouped by families. (**E**) Percentage distribution of the in silico calculated pI. The results have been represented by the three groups of families established in this analysis: *Brassicaceae* (Bra, blue), *Fabaceae* (Fab, orange), and *Other* families (Other, grey).

The SST protein sequences have been deduced from all 194 CDSs found in the databases (List S2). According to different prediction tools, the SST proteins had a signal peptide (SP) (Figure 3A) that was missing from only six sequences (#59, 60, 108, 126, 142, and 175 in Table S1). Of these six sequences, only #175 featured the above-mentioned gene structure, while the remaining sequences presented abnormal first exons: short (#59, 60, and 126), extra-short (#142), or extra-long (#108). These gene sequences were probably misannotated in the databases and thus were not included in subsequent analyses. However, we confirmed that they showed the typical features of mature SST (mSST) proteins described herein.

The 188 SSTs showing an SP were further analysed, and we observed that the length was dependent on the taxonomic family (Table S1, Figure 3B). In *Brassicaceae*, it was predicted to have 26 residues in 72.2% of the sequences, while in *Fabaceae* and *Other* families it often had 22 amino acids (46.5% and 40.23%, respectively) (Figure 3B). Sixteen proteins from different families (8.25%) presented a larger SP with at least 30 amino acids, but regardless of this feature they have been identified as SPs by several algorithms. Apart from the SP, no other transmembrane domains were predicted in silico.

The SST proteins studied ranged from 66 amino acids (#98) to 138 (#162) (Table S1). Once processed, the predicted size of the mSST (List S3) varied from 40 amino acids in *M. truncatula* SST2 (#83) to 114 in *Malus domestica* SST2 (#162) (Table S1, Figure 3C) which correspond to Mw values between 4.6 and 13.5 kDa (Figure 2D). Among them, the vast majority (86.7%) comprised 51 to 90 residues and had an Mw from 6 to 11 kDa. Regarding the seven sequences with less than 50 amino acids found in four genera, *Glycine*, *Medicago*, *Amaranthus,* and *Manihot* (#65, 67, 72, 73, 83, 98, and 131 in Table S1), they seemed to be truncated at the C-terminal end and were possibly misannotated sequences. These seven SSTs were not considered in comparative analyses hereafter.

Also, we found differences in size depending on the taxonomic family, mSSTs from *Brassicaceae* are highly homogeneous with 51 to 60 amino acids (Figure 3C) and 6.25 to 7.03 kDa (Figure 3D) (Table S1). Within this size range, there were only two proteins from non-*Brassicaceae* species: *Carica papaya* (#112) and *Solanum tuberosum* (#188). In *Fabaceae,* most SSTs (87.8%) have from 61 to 90 amino acids resulting in a higher Mw (Figure 3C,D). The size range of SSTs among the group of *Other* families, which includes 37 different families, was broader, but the majority had from 61 to 90 amino acids, similar to *Fabaceae* (Figure 3C). Additionally, there were 18 sequences showing more than 90 amino acids (#111, 123, 128, 129, 132, 134, 143, 162–165, 167–170, and 192–194) and belonging to 10 taxonomic families (*Cannabaceae*, *Ericaceae*, *Euphorbiaceae*, *Fagaceae*, *Litraceae*, *Rosaceae*, *Rubiaceae*, *Rutaceae*, *Ulmaceae,* and *Vitaceae*) (Table S1, Figure 3C). These larger SSTs with no other known functional domain were considered as SSTs but will not be included in the comparative studies below.

The pI of mSST varied from 4.38 (#119, 120) to 9.90 (#47) (Table S1). Remarkably, several proteins from *Brassicaceae* (46.3%) and *Fabaceae* (51.2%) had a pI between 9.01 and 10.00 (Figure 3E). Regarding post-translational modifications, most mSST proteins might undergo phosphorylation by different kinds of protein kinases, mostly casein kinase II phosphorylation site (CK2) and protein kinase C phosphorylation site (PKC) (Table S1). Sites for other post-translational changes were scarce within SST sequences, such as N-glycosylation (4%), myristoylation (13%), and amidation (4%).

With the aim to predict SST subcellular localisation in silico, we analysed the sequences to find motifs associated with protein sorting. The presence of an SP in SST proteins (Table S1) indicated that they enter the secretory pathway. Thus, the WoLF PSORT tool [40] indicated that most SSTs enter the ER (64.4%) to reach the extracellular compartment (37.6%), the vacuole (26.8%), the chloroplast (21.6%), or the mitochondrion (4.1%) (Table S1). However, the TargetP 2.0 tool [32] did not support the targeting to such organelles, despite confirming the existence of a SP. Likewise, using DeepLoc [34], the prediction for SST subcellular localisation is mostly extracellular (66.5%), followed by vacuolar (22.2%).

### 3.4. Mature SST Sequences Group by Taxonomic Families and Have Several Conserved Features

**Figure 4:**
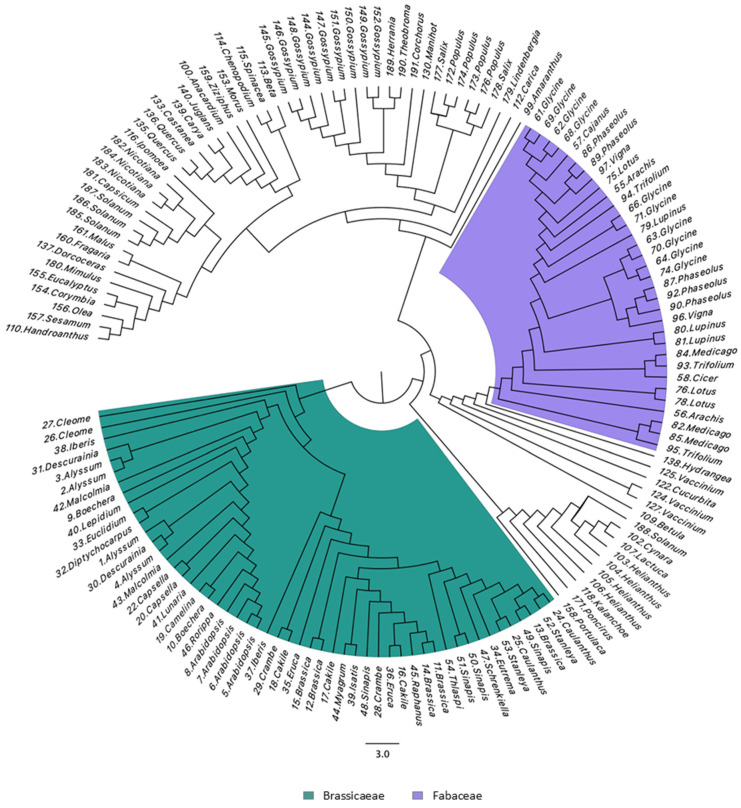
Dendrogram of the SST sequences. Multiple alignment was performed in the CLUSTAL OMEGA tool, applying the ClustalW output with the 151 mature full-length SST sequences listed in List S4. A guide tree was selected, and a dendrogram was made using FigTree v1.4.4. Sequences clade according to taxonomic subclass and even to family.

**Figure 5:**
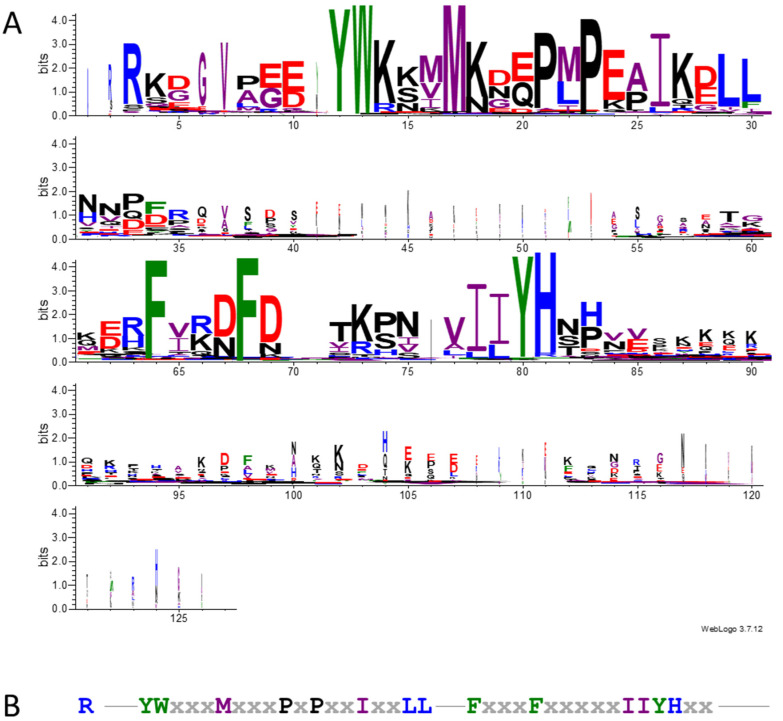
WebLogo of the alignment of mature SST proteins with scaled stack width. (**A**) The WebLogo includes the sequences listed in List S4, excluding #109. WebLogo consists of stacks of symbols: one stack for each position in the sequence alignment. The overall stack’s height indicates sequence conservation at that position, while the symbol height within the stack indicates the relative frequency of each amino acid at that position. The use of a scaled stack width gives the letters different widths depending on the number of sequences that have a given letter. Amino acids are coloured-coded for clarity: D and E are in red; Y, W, and F in green; L, R, and H in blue; A, V, L, I, G, and M in purple; and K, S, T, N, Q, C, and P in black. Bit: measure of conservation at a particular sequence position; the maximum conservation for a given amino acid in a sequence is 4.32 bits. (**B**) Consensus sequence displaying the most conserved amino acids in the mature protein showing two separate regions.

**Figure 6:**
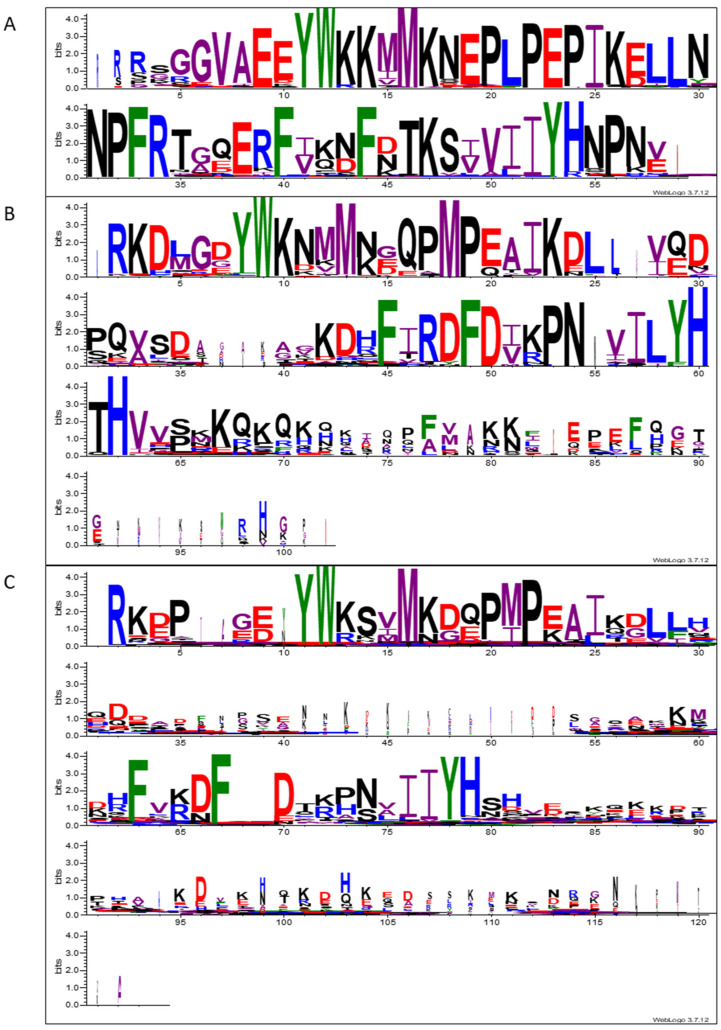
WebLogo of the mature SST proteins with scaled stack width sorted by taxonomic family. (**A**) *Brassicaceae*. (**B**) *Fabaceae*. (**C**) *Other* families. The characteristics of the WebLogo are detailed in the legend of Figure 5.

A preliminary visual analysis of the SST sequences revealed a greater similarity among proteins within the same taxonomic groups (Table S1). To assess the grouping by families and identify other conserved residues in SST sequences, we obtained a series of phylogenetic trees [25] (Figure 4 and Figure S1), and multiple sequence alignments were visualised with the WebLogo tool [47] (Figure 5, Figure 6 and Figure S2). As indicated in Section 2, all the SST sequences used in these analyses correspond to mature proteins and are listed in List S4.

The dendrogram using the 151 sequences (List S4) showed that SSTs from *Brassicaceae* (green) and *Fabaceae* (purple) were split into two groups according to their taxonomic classification (Figure 4). It is interesting to note that *Cleome violacea* appears on the margin of the *Brassicaceae*, which agrees with some classifications that include this genus in the family *Cleomaceae*, also in the order Brassicales.

To better visualise how the SSTs of *Other* families are grouped, a separate dendrogram was made and is presented in Figure S1. The SST sequences belonging to *Other* families showed more similarity within sequences from their own families, with some exceptions. For example, the SST2 from *Solanum tuberosum* (#188) was not grouped with other *Solanaceae*, which can be explained by its smaller size (between 15 and 39 amino acids less) and the SST1 from *Betula platyphylla* was not found in *Fagaceae*, possibly due to differences in its core zone (List S4). Also, the SSTs from *Scrophulariaceae* (#179 and 180) were not grouped together; within *Ericaceae*, SST3 from *Vaccinium darrowii* (#125) appeared separated from SST2 and SST5. It was also evident that the similarity in some cases was higher than at the family level; thus, most sequences from Malvids clustered together despite belonging to different families (Figure S1).

The WebLogo representations of the SST sequence alignments (Figure 5 and Figure 6) were made using a height scale for amino acid conservation at a given position and a width scale for the number of valid amino acids in one position (gap frequency in that position). The sequence of *B. plathyphylla* (#109) was excluded from the analysis as it caused significant distortion of the results. Figure S2 displayed the alignments using only the height scale. Figure S2A includes the #109 sequence showing that between the letter I in positions 80 and 90 there are nine amino acids, exclusive of sequence #109, which are responsible, in part, for its position in the dendrogram (Figure S1) and for the distortion it generated in the WebLogo.

Focusing on the amino acid sequence, 85.6% of the SSTs started with an R in the first position of the mature protein and had the amino acid pair YW (Figure 5). Some of the SST sequences analysed (#21, 23, 77, 88, 91, 117, 119, 120, 121, 141, and 166 in Table S1) had the R a few residues downstream from the predicted signal peptide cleavage site, so it might be inaccurate. The spacing between the initial R and the YW was variable, mostly five residues but frequently seven in *Brassicaceae* (Table S1).

There were other amino acids conserved in the SST sequences (Figure 5A), such as the M at position 17, the motif P-X-P (positions 21 to 23, where X is any amino acid, frequently M or L), and residue I at position 26. After a less-conserved zone in the protein, we found two Fs separated by three residues (positions 64 to 68) with the second one often surrounded by D or N. Also, there were I and/or I/L in positions 78/79 followed by YH. From this H, SSTs were less conserved in terms of composition and length (Figure 5A). The conserved regions are summarised in Figure 5B.

Given that the similarity was greater between members of the same family, we separately obtained the multiple sequence alignments of the *Brassicaceae*, *Fabaceae,* and *Other* families (Figure 6A–C and Figure S2B–D, respectively). In Cruciferae, the SST sequence was extremely conserved, with single amino acids prevailing at most positions (Figure 6A and Figure S2B). All the proteins had similar lengths; hence, the alignment contained few gaps, as can be observed in the WebLogo representation using a width scale (Figure 6A). The main difference among SSTs from Cruciferae was the number of amino acids from the initial R to the conserved Y (Figure 5A), which ranged between 5 and 10 in the different sequences (Table S1). Notably, a third conserved F close to the other two referred to above occurred (Figure 6A).

The sequence alignment of the *Fabaceae* showed that SST proteins are less conserved within this family (Figure 6B and Figure S2C) than in Cruciferae. However, in *Fabaceae,* the distance from the first R to the conserved Y, which is usually five residues, was less variable.

As observed in the general analysis, there are two areas clearly more conserved in SSTs (Figure 5). The length of the interzone between the two conserved regions was shorter in *Brassicaceae*, with only 10 amino acids (Figure 6A), whereas there were 19 residues in *Fabaceae* (Figure 6B) (between positions 1 to 26 and 45 to 62) and 34 amino acids in *Other* families (Figure 6C). We found several differences between the alignments of the SST sequences from *Fabaceae* and *Brassicaceae*. After the initial R, the SSTs in *Fabaceae* often displayed a K and a D, whereas these positions were not conserved in *Brassicaceae* (Figure 6A,B). Additionally, in *Fabaceae*, there was a clear preference for an M as the middle amino acid in the conserved P-X-P (position 18, Figure 6B), and the same amino acid was less conserved in *Brassicaceae*, often being an L (position 21, Figure 6A). In this zone, *Brassicaceae* SSTs presented a third P, with the sequence P-L-P-E-P being highly conserved (positions 20 to 24 in Figure 6A). Also worth noting, the C-terminal conserved YH observed in all the SST sequences was followed by a second H in *Fabaceae*, showing the pattern YH-X-H (with X usually being a T, positions 59 to 62 in Figure 6B). As expected, the alignment exclusively using sequences from *Other* families presented fewer well-conserved amino acids, apart from the characteristic SST consensus positions described previously (Figure 6C). However, the WebLogo representation showed that some features described for *Fabaceae* were sometimes present in SSTs from *Other* families. Specifically, we observed the prevalence of the pair RK at the beginning of the mature protein, the P-M-P sequence, or the second H after the well-conserved YH (positions 2 and 3, 20 to 22, and 58 to 61 in Figure 6C, respectively). Finally, the YH conserved region found in all SSTs was usually preceded by two Is (I-I-Y-H) in *Brassicaceae* (positions 51 and 52 in Figure 6A) and *Other* families (positions 76 and 77 in Figure 6C), while in *Fabaceae* the second I is frequently an L (I-L-Y-H) (positions 57 and 58 in Figure 6B).

### 3.5. SST and ST Proteins Share the N-Terminal Conserved Region

**Figure 7:**
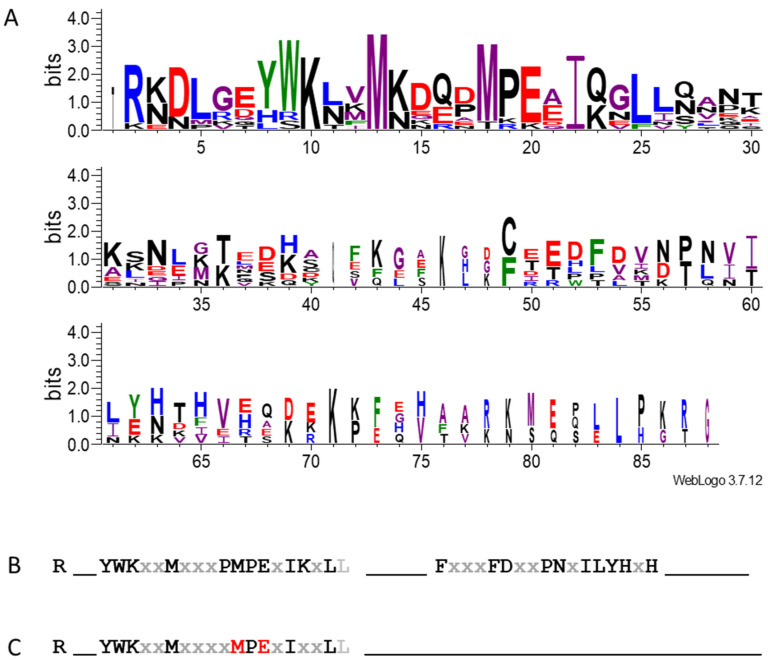
WebLogo of the alignment of the ST non-repeat zone and the mature SST with scaled stack width. (**A**) WebLogo representation of conserved amino acids in the 6 MtST non-repeat sequence and the 4 MtSST mature proteins of *Medicago truncatula*. (**B**) Consensus sequence in *Fabaceae* SSTs. (**C**) Consensus sequence of *M. truncatula* STs and SSTs. The characteristics of the WebLogo are detailed in the legend of Figure 5.

As already mentioned, SSTs were identified via sequence-similarity searches using the non-repeat zone of the ST proteins. To find differential characteristics between these two kinds of proteins, we aligned the four *M. truncatula* MtSSTs (#82, 83, 84, and 85) and the non-repeat zone of the six MtSTs [8] (Figure 7). WebLogo analyses of the SSTs and the non-repeat zone of the STs of *M. truncatula* were also performed separately (Figure S3).

The analysis showed that MtST and MtSST only share the N-terminal conserved region (Figure 7), where they are highly homologous. Both protein types began with an R and showed the pair YW five amino acids downstream. They also featured two Ms at positions 13 and 18, a P at position 19, an I at position 22, and an L at position 25 (Figure S3). However, in the ST proteins some positions are not as well conserved as in SSTs (highlighted in red in Figure 7C) (Figure S3). The second conserved region characteristic of SST proteins with two Fs separated by three residues was apparently absent in the WebLogo including both ST and SST (Figure 7C). Although, around the second F (position 53 in Figure 7A), we can observe the conserved sequence: DFDxxPxxxxY (Figure S3). An important difference between the SST and ST proteins is the absence of the well-conserved C found in the non-repeat region of the ST proteins (Figure S3).

### 3.6. The Genetic Environment and Co-Expression Analysis of the SST1 Gene from Arabidopsis Point to a Relationship with a BURP Protein

**Figure 8:**
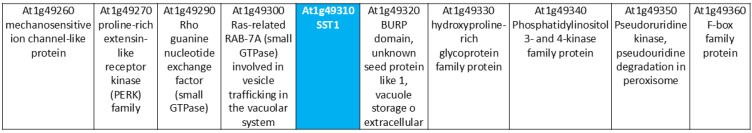
Genomic environment of the *SST1* gene. The *SST1* gene (At1g49310) is located on chromosome 1 of *Arabidopsis thaliana* (highlighted in blue), as determined using the JBrowse tool in the Phytozome database. Four upstream and five downstream genes are included in the figure, indicating the gene number and the encoded protein.

To obtain empirical results for the SST family of proteins, we focused on the *SST1* gene (At1g49310) of Arabidopsis, the only gene encoding an SST protein in this species. Sometimes the genetic situation of a gene allows us to infer possible interactions and/or functions. The environment of the *SST1* gene has been analysed using the JBrowse database [57,58] (Figure 8). The genes immediately flanking *SST1* upstream and downstream encode a Ras-related RAB-7A (RABG3E) (At1g49300) and a BURP protein named unknown seed protein like 1 (USPL1) (At1g49320), respectively. Other genes up- and downstream of *SST1* are shown in Figure 8 and encode proteins related to cell signalling, such as the mechanosensitive ion channel-like protein, proline-rich extensin-like receptor kinase (PERK) family, phosphatidylinositol 3 and 4-kinase family protein, pseudo uridine kinase, and F-box family protein. Also, we found a gene encoding a cell-wall hydroxyproline-rich glycoprotein that might be related to the above-mentioned PERK. The genomic environment of the genes encoding the ST and SST proteins of *M. truncatula* is included in Figure S4 as the results will be used to support the discussion.

Considering genes co-expressed with *SST1* according to the ATTEDII database [59], among the top three genes, we found the highly co-expressed *USPL1* gene (co-expression z score of 14.1) and two other genes that presented significant co-expression rates, encoding a tyrosine-sulphated protein of unknown function (PSY3) (At2g29995) (co-expression z score of 7.5) and a major facilitator superfamily protein (At1g33440) (co-expression z score of 5.4).

### 3.7. SST1 Promoter Activity Is Found Mainly in Roots

**Figure 9:**
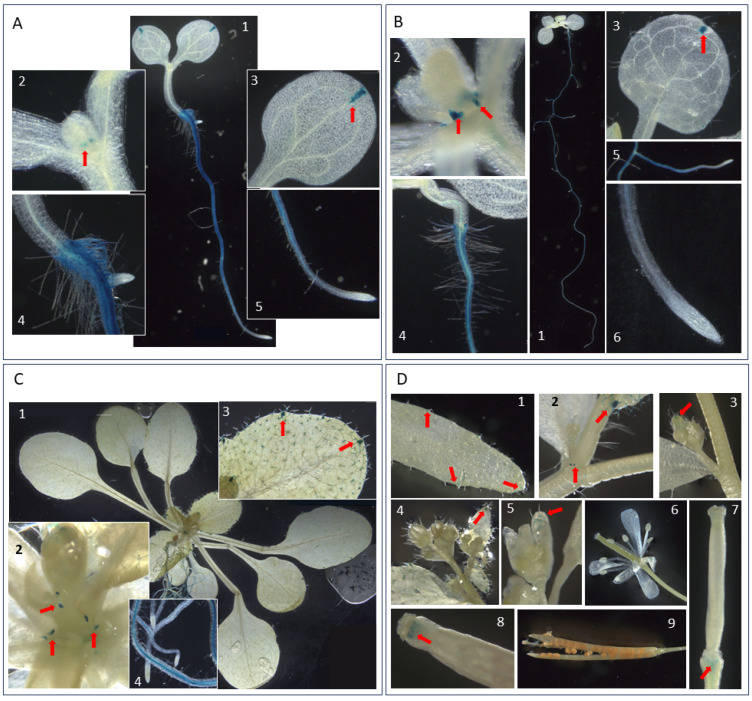
GUS activity of pSST1::GUS transgenic *Arabidopsis thaliana* plants. (**A**) 5 d old seedling (1), detail of the shoot apex (2), a cotyledon (3), the junction zone between the hypocotyl and the radicle with a lateral root (4), and the root apex (5). (**B**) A 10 d old plantlet (1), detail of the shoot apex (2), a cotyledon (3), the junction zone between the hypocotyl and the radicle (4), a lateral root (5) and the root apex (6). (**C**) A 20 d old plant (1), detail of the shoot apical zone (2), leaf (3), and root apices (4). (**D**) Organs of plants older than 30 d: cauline leaf (1), branch insertion zone (2), floral bud (3), flowers at different stages of development (4), flower before fertilisation (5), flower after fertilisation (6), immature silique (7), detail of immature silique tip (8), and mature silique (9). The red arrows show the areas with GUS activity.

To find the putative function of a protein, a preliminary step is to determine where the encoding gene is expressed. Therefore, pSST1::GUS transgenic plants were analysed throughout the vegetative and reproductive parts of the life cycle of the plant (Figure 9). GUS activity and hence promoter *SST1* (pSST1) activation was mainly observed in the roots (Figure 9(A1,B1,C4)). The staining extended through the entire organ except in the root apex (Figure 9(A4,B4,C4)), with more intense labelling in the vascular cylinder of the primary root (Figure 9(A5,B6,C4)) and lateral roots (Figure 9(A4,B5)). Furthermore, light did not influence the pSST1 activity in seedlings, as the etiolated seedlings showed the same pattern as the green ones (Figure S5).

In addition to the observed root staining, a faint blue colouration was detected in the zone of leaf primordium formation in 5 d old plants (Figure 9(A2)), which intensified in 10 d old (Figure 9(B2)) and 20 d old (Figure 9(C2)) plants and appeared in the basal lateral part of the newly formed leaves, which is the zone that gives rise to the serrate margin. Furthermore, pSST1 activity was found in the distal hydathode of cotyledons in seedlings of both ages (Figure 9(A3,B3)) and in adult plant leaves (Figure 9(C3)), where it was also detected in the trichomes of leaves (Figure 9(C3)), cauline leaves (Figure 9(D1,D2)), and sepals (Figure 9(D3,D5)).

During the reproductive phase, pSST1 activity was detected in the cauline leaf axil as the inflorescence developed (Figure 9(D2)). No activity was observed in any other floral organ (Figure 9(D4,D6)), except in the immature siliques, where a faint blue colour was seen in the upper part of the pedicel and in the style (Figure 9(D7,D8)). Finally, pSST1 activity was detectable in neither the mature siliques nor in the seeds (Figure 9(D9)).

### 3.8. The Accumulation of SST1 Transcripts Confirms Their Relationship with Root Physiology

**Figure 10:**
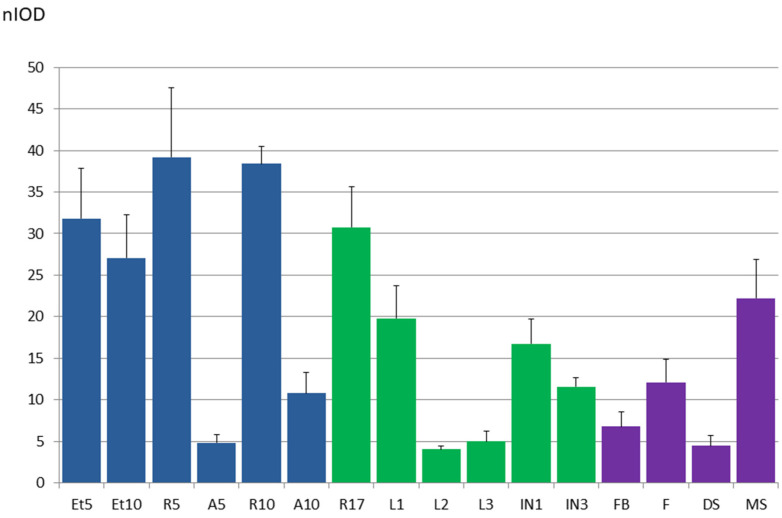
Levels of *SST1* transcript accumulation throughout plant development measured by sqRT-PCR. The bar chart represents the relative quantification of *SST1* transcript bands from PCR, normalised to an endogenous control set at 100 units of normalised and integrated optical density (nIOD). In blue, the levels of *SST1* transcripts in 5 and 10 d old seedlings: etiolated (Et) and green seedling roots (R) or aerial parts (A). In green, the levels in the roots (R), first (L1), second (L2), and third (L3) pair of leaves from 17 d old plants, and the first (IN1) and third internode (IN3) from 32 d old plants. In purple, the levels in the flower buds (FB), flowers (F), developing siliques (DS), and mature siliques (MS) of *Arabidopsis thaliana* plants. These results are based on several sqRT-PCR replicates, with bands from all the electrophoresis gels quantified and averaged. Figure S6 shows a representative agarose gel.

To contrast the results obtained by studying the activity of pSST1 (Figure 9), we analysed the accumulation of *SST1* transcripts in different plant organs during development (Figure 10). We found a significant accumulation in 5 and 10 d old etiolated and green seedlings (Figure 10 and Figure S6). In green seedlings, the root was examined separately from the aerial tissues (hypocotyl, cotyledons, and leaves) and *SST1* transcripts accumulated mostly in the radicles, corroborating the results obtained previously (Figure 9). Similarly, in adult plants (Figure 10), the level of *SST1* transcripts was clearly higher in the roots. In the aerial organs of adult plants, there were more transcripts in the first pair of leaves, the most developed ones, than in the second and third pairs. In addition, the *SST1* mRNA levels in the internodes were not very high, reaching higher amounts in the first internode than in the third internode, which was still growing (Figure 10).

Finally, the accumulation of the *SST1* transcript was studied in reproductive organs (Figure 10), where its level was low. A higher accumulation was observed in flowers than in flower buds. In fruits, *SST1* transcripts were up to 4-fold more abundant in mature siliques than in young ones, in contrast to pSST1 activity (Figure 9(D9)), but still did not reach the level found in roots.

### 3.9. SST1 Protein Accumulates Around Vascular Bundle in Roots

**Figure 11:**
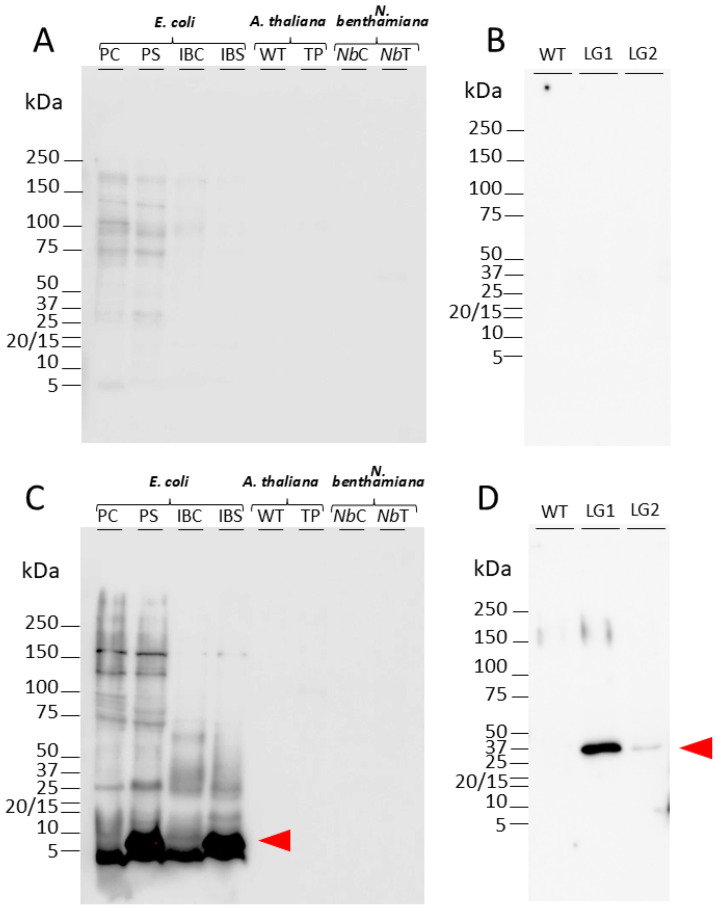
Western blot performed with anti-SST1 antibodies. Western blot performed with pre-immune (**A**,**B**) or immune serum (**C**,**D**) against the synthesised peptide from the SST1 sequence (YWKKKMMKNEPLPEPIK). (**A**,**C**) Western blot performed using total proteins (PC) and purified inclusion bodies (IBC) from control *Escherichia coli* colonies that do not produce the SST protein and those that do (PS and IBS) after 5 h of IPTG induction; protein extracted from 5 d old seedlings of Arabidopsis WT (WT) and p35S::SST1 transgenic plants (TP); and protein extracts from *Nicotiana benthamiana* leaves, control (NbC) and agroinfiltrated with the p35S::SST1 construct (NbT). The red arrow in (**C**) points to the putative band containing the mSST1 protein produced in *E. coli*. (**B**,**D**) Western blot performed using proteins extracted from the roots of 25 d old Arabidopsis WT plants and two lines of p35S::SST1-GFP transgenic plants (LG1 and LG2, respectively). The red arrow in (**D**) points to the putative band of the recombinant SST1 protein fused to GFP purified from transgenic plants.

A specific polyclonal antibody (anti-SST1) was generated and purified as indicated in Section 2, and it was tested against recombinant and native SST1. The mSST1 protein was produced in *E. coli* after induction with IPTG, resulting in a band above the 10 kDa marker that increased progressively until 5 h post-induction (Figure S7). Also, we produced the protein in the heterologous *N. benthamiana* system to test the antibodies. The rabbit pre-immune serum did not recognise any specific band in Western blotting using the protein extracts from different sources, i.e., *E. coli*, Arabidopsis, and *N. benthamiana* (Figure 11A,B).

The anti-SST1 antibodies can strongly recognise the mature protein produced in the bacteria, both in the total protein extract (PS) and in the inclusion bodies (IBS), close to its estimated molecular weight of 6.6 kDa (Figure 11C). Furthermore, these antibodies were not able to recognise such a band in the extracts from the control colony in which mSST1 is not produced (PC and IBC). The bands that appeared in all the *E. coli* extracts, even the 4 kDa band, are due to unspecific binding as they also appeared in the control extracts (PC and IBC). We also tested whether the antibodies recognised the native protein produced by plants and its specificity. The anti-SST1 antibodies were not able to recognise the native SST protein extracted from 5 d old Arabidopsis seedlings, WT or overexpression lines, or in agroinfiltrated leaves of *N. benthamiana* (Figure 11C). Due to the major expression of the *SST1* gene in roots, we tested protein extracts from this source to enrich the sample, but this produced a negative result (Figure 11D). Finally, protein extracts from the roots of two specific lines of transgenic plants producing SST1-GFP (LG1 and LG2) were used. A single band corresponding to the size of the fusion protein was detected (Figure 11D), indicating that the antibody anti-SST1 can specifically recognise the recombinant SST1-GFP protein.

**Figure 12:**
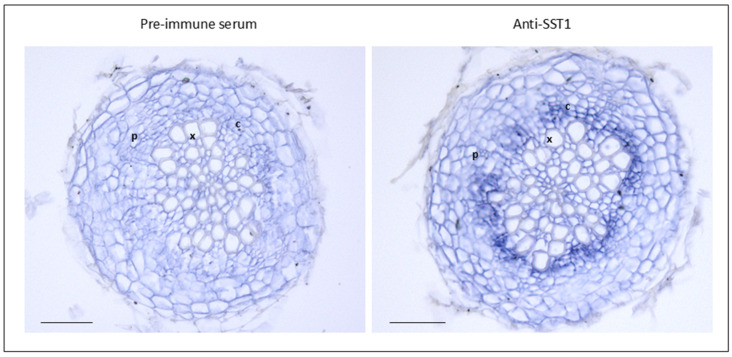
Immunolocalisation of SST1 in roots of 25 d old *Arabidopsis thaliana* plants. Cross-sections were taken from basal regions of the root and treated with either pre-immune serum (**left**) or anti-SST1 (**right**). c: cambium; p: phloem; x: xylem. Scale bars: 50 µm.

Finally, an immunocytochemical study using anti-SST1 antibodies was performed. We checked for the presence and tissular distribution of the protein in basal root sections of 25 d old *A. thaliana*. The SST1 epitope was detected in the roots, and the labelling was especially strong in the cambial cells around the vascular cylinder (Figure 12).

### 3.10. SST1 Protein Co-Localises with the Endoplasmic Reticulum and Vacuole

**Figure 13:**
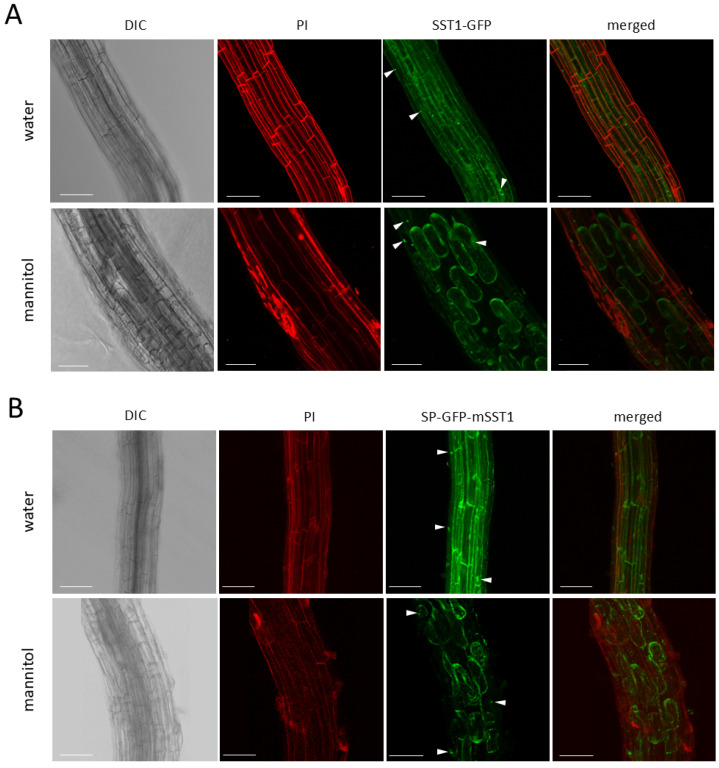
Subcellular localisation of *Arabidopsis thaliana* SST1 fused to GFP. Representative confocal microscopy images of roots producing (**A**) the SST1 protein fused to GFP at the C-terminus or (**B**) the protein fused to GFP at the N-terminus. The roots were mounted in water or in 0.6 M mannitol. Left to right, columns display the following: DIC, differential interference contrast micrographs of the roots analysed; PI, red fluorescence from propidium iodide-stained cell walls; green fluorescence from GFP fused to SST1; and merged signals from the red and green channels. The images are z-projections. Arrowheads point some denser granule-like GFP accumulation. Scale bars: 50 µm.

We investigated the subcellular localisation of SST1 tagged with GFP in the roots of 20 to 25 d old Arabidopsis plants (Figure 13). We analysed transgenic plants with either p35S::SST1-GFP (Figure 13A) or p35S::SP-GFP-mSST1 (Figure 13B). Regardless of the construction, confocal microscopy images of green GFP fluorescence showed that the SST1 protein accumulated inside the cell, showing a diffuse distribution and some spherical bright granules marked with arrowheads (Figure 13A,B). Overall, the green fluorescence in SST1-GFP was less intense than that in SP-GFP-mSST1, whereas granules were more abundant in the former. The green signal in the cell periphery observed in the water-mounted roots seemed to overlap with the PI-stained cell walls (red fluorescence) when both channels were merged (Figure 13A,B). To confirm the absence of SST1 in the extracellular compartment, the roots were mounted with mannitol to plasmolyse the protoplast. Irrespective of the C-terminal (SST1-GFP) or N-terminal (SP-GFP-mSST1) GFP fusion, no green fluorescence was detected in the cell wall (Figure 13A,B). In the mannitol-mounted roots, the green signal shrunk back—indicating that SST1 was restricted to the protoplast, which was clearly observed when the red and green channels were merged (Figure 13A,B).

**Figure 14:**
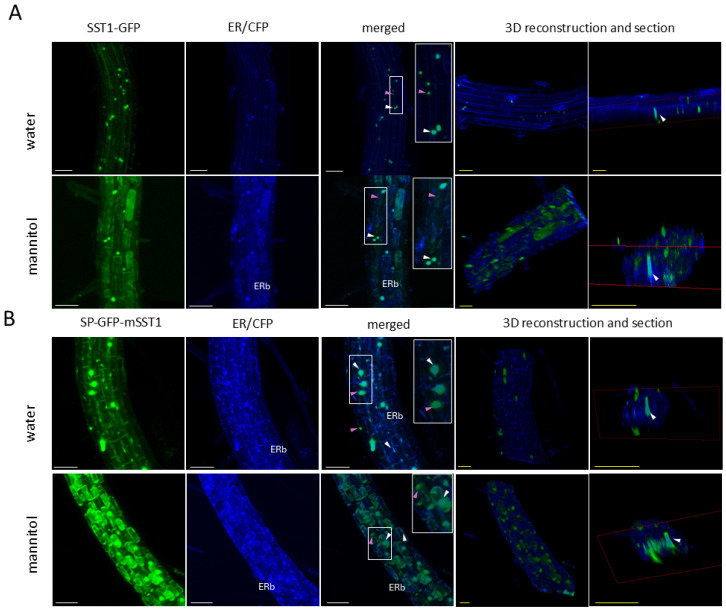
Subcellular localisation of *Arabidopsis thaliana* SST1 fused to GFP and the cyan ER marker. Representative confocal microscopy images of roots producing (**A**) the SST1 protein fused to GFP at the C-terminus or (**B**) at the N-terminus, together with an endoplasmic reticulum protein marker tagged with cyan fluorescent protein (ER/CFP). The roots were mounted in water or in 0.6 M mannitol. Left to right, columns display the following: green fluorescence from GFP fused to SST1; blue fluorescence from the ER protein marker; merged signal from the green and blue channels; 3D reconstruction with the projection of green and blue signals and one representative transverse section (red lines). The images are z-projections. White arrowheads point to overlapping green and blue fluorescence; pink arrowheads point to non-overlapping green zones. ERb: Endoplasmic reticulum fusiform bodies. White scale bars: 50 µm; yellow scale bars: 100 µm.

**Figure 15:**
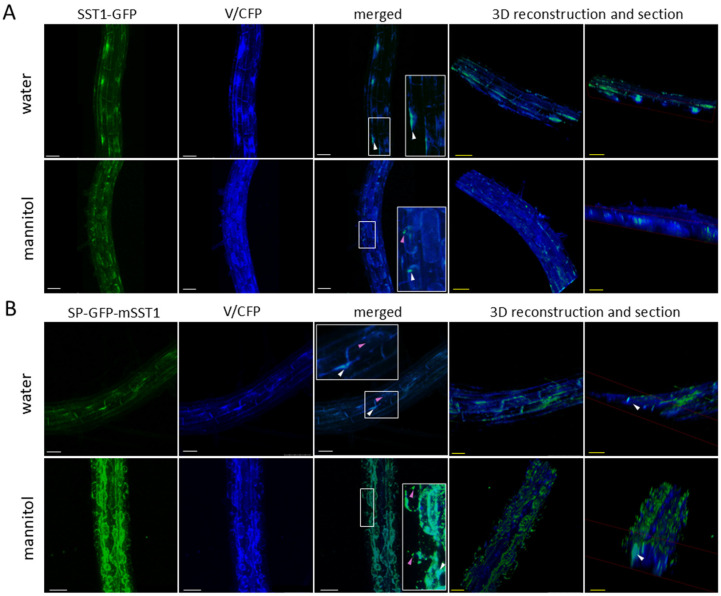
Subcellular localisation of *Arabidopsis thaliana* SST1 fused to GFP and the cyan vacuole marker. Representative confocal microscopy images of roots producing (**A**) the SST1 protein fused to GFP at the C-terminus or (**B**) at the N-terminus, together with a vacuole protein marker tagged with cyan fluorescent protein (V/CFP). The roots were mounted in water or in 0.6 M mannitol. Left to right, columns display the following: green fluorescence from GFP fused to SST1; blue fluorescence from the vacuole protein marker; merged signal from green and blue channels; 3D reconstruction with the projection of green and blue signals and one representative longitudinal or transverse section (red lines). The images are z-projections. White arrowheads point to overlapping green and blue fluorescence; pink arrowheads point to non-overlapping green zones. White scale bars: 50 µm; yellow scale bars: 100 µm.

Notably, SST1 fused to GFP sometimes occurred in dense granules within the cytoplasm or accumulated preferentially in some areas (Figure 13, arrowheads). Therefore, we analysed double-transgenic plants bearing either of the abovementioned transgenes p35S::SST-GFP or p35S::SP-GFP-mSST together with one of the three different transgenes encoding CFP-bound organelle marker proteins, namely, the ER, the vacuole, and the Golgi apparatus. We found a partial co-localisation of SST1 with the ER (Figure 14) and vacuole (Figure 15), while the protein was not detected in association with the Golgi apparatus (Figure S8).

Spherical granules of different sizes corresponding to the SST1-GFP or GFP-SST1 fusion proteins overlapped with the ER marker protein (Figure 14A,B, merged). When ER fusiform bodies were observed, they sometimes coincided with green fluorescence, especially with GFP-SST (Figure 14B), but the signal was weaker than that in the granules. Also, a scattered GFP signal, which was more evident in the mannitol-plasmolysed cytoplasm, was detected independently of blue ER areas (Figure 14, merged), indicating other localisations of SST1 within the cell. Additionally, we observed a partial coincidence of SST1-GFP or GFP-SST1 fluorescence with CFP-tagged vacuoles (Figure 15A,B, merged). Usually, the overlapping areas with the vacuole were more diffuse than those observed in the ER, and the granules with co-occurring fluorescence were smaller. Moreover, we observed a brighter GFP signal around the nuclei in water-mounted roots of SST1-GFP and GFP-SST1 (Figure 15A,B, water, white arrowheads).

The 3D reconstruction of roots merging z-planes from the green and blue channels and the subsequent digital scanning of perpendicular sections allowed us to confirm the co-localisation of GFP with the ER (Figure 14) or the vacuole-associated CFP (Figure 15) in several regions, as well as the existence of zones where fluorescence was not correlated. Also, these digital sections showed that SST1 N- or C-terminally fused to GFP coincided with both organelles, but the overlap was not complete. Nevertheless, we checked for the presence of SST1 in the Golgi apparatus and could not detect any correspondence between the GFP in the fusion proteins SST1-GFP or GFP-SST1 and the Golgi cyano-tagged protein (Figure S8). The green fluorescence in these roots resembled that observed in the previously analysed transgenic plants, with some bright spots and disseminated fluorescence within the cell, including some ER-body-like structures, as clearly observed in the mannitol-mounted p35S::SP-GFP-SST1 roots (Figure S8). Neither merged blue plus green channels nor 3D reconstruction and sectioning (Figure S8) allowed us to detect overlapping fluorescence, indicating a relationship between SST1 and the Golgi apparatus.

## 4. Discussion

Proteins belonging to PF10950 feature DUF2775, whose biological meaning remains elusive. In this family, there are two main types of proteins, the previously known ST proteins [8] and the SST proteins, which we introduce in this work. Both types have an SP followed by a zone with conserved residues, known as the non-repeat region. In addition, ST proteins have another zone consisting of highly conserved tandem repeats, which accounts for their larger size [8].

Here, we defined SST proteins as small ST proteins containing a DUF2775 domain and having neither ST-like tandem repeats nor other known functional domains. No SST sequences are found in taxonomic groups outside the plant kingdom, similar to the ST proteins [8]. Additionally, the SSTs are restricted to the most recently evolved plant species (Figure 1), and our results indicate that they appeared evolutionarily with the core eudicots, as they are absent in the most primitive basal angiosperms, basal eudicots and *Laurales* (as defined by [60,61]). Therefore, the SSTs have a broader distribution in the plant kingdom than STs, which are not found in some families of core eudicots like the *Brassicaceae* [8], indicating an earlier emergence.

The structure of *SST* genes with a small first exon and a single intron next to the 5’ end (Figure 2) resembles that of *ST* genes, although the second exon is longer in the *ST*, as it also encodes the tandem repeat region [8]. The SST proteins display an SP of variable length depending on the taxonomic family (Figure 3), but no SST has been purified yet, so the post-translational processing of the SP has not been proved. In most cases, SST size is a taxonomic family-related feature, being smaller in *Brassicaceae*, where mature proteins range from 51 to 60 amino acids (Table S1). Usually, the ST and SST proteins from a given species are clearly differentiated by their size, i.e., in *M. truncatula,* STs range from 359 to 493 amino acids [8] whilst SSTs have around 79 amino acids (Table S1). The wide range of pI in mSST (Figure 3) makes it difficult to predict SST behaviour in cellular compartments with a different pH. Remarkably, several SST proteins had a pI above 9, which indicates that these SSTs would have a positive charge in almost any subcellular localisation and could interact with negatively charged cell components.

There are well-conserved amino acids in the different SSTs; in general, mature proteins start with an R and show the YW pair in the N-terminal region (Table S1, Figure 5), both of which are characteristic of ST proteins as well [8]. By performing different sequence alignments and analysing their WebLogo representations, we identified other conserved residues among SSTs (Figure 5, Figure 6 and Figure S2). Overall, we established the SST consensus sequence as Rx_5–7_YWxxxMxxxPxPxxIxxLLx_n_FxxxFxxxxxIIYHx_m_ (Figure 5B), consisting of two major zones separated by a variable number of residues (x_n_). Nonetheless, the SST proteins have some family-associated characteristics as observed in the dendrograms, where the sequences are distributed by taxonomic families with minor exceptions (Figure 4 and Figure S1). The SST proteins of cruciferous plants are extremely conserved (Figure 6A), much more so than those of legumes (Figure 6B) and the group of *Other* families (Figure 6C). The main differences between the SSTs of different families are found in the interzone x_n_, between the two most conserved areas (Figure 5B). Also, the larger size observed in the SSTs from families other than *Brassicaceae* (Figure 3C) was due to the length of this interzone, together with the variable C-terminal region (x_m_) in the consensus sequence (Figure 6). Hence, we deduce that the distance between the conserved regions is not determinant for the protein’s functionality, but the two conserved regions must be important as they are clearly conserved irrespective of the species.

Additionally, we further explored the similarity between the SST protein sequence and the non-repeat region within the ST proteins and found that the homology is restricted to the first part of the consensus sequence of the SST before the x_n_ interzone (Figure 7 and Figure S3). This result raised a question about the DUF2775 architecture in the two kinds of proteins within PF10950. An in-depth analysis of MtSST proteins (Figure S3) revealed that the conserved region after the x_n_ interzone (DFDxxPxxxxY) partially fits with the consensus sequence of the ST canonical repeat: EFEPRPxxxxY [8]. This roughly conserved repeat resembles the first repeat in all ST proteins, which always shows a more imperfect sequence than the following ones [8]. Therefore, the N-terminal regions of SST and ST are partially conserved and adjacent to a non-canonical ST-like repeat sequence. This configuration might be the minimal functional architecture of the DUF2775 domain that defines the PF10950 [1]. However, ST proteins can contain a greater number of DUF2775 domains depending on the number of tandem repeats they have. Noteworthily, according to [16], the motif xxxxY that appears once in the SSTs (Figure 5 and Figure 6) and once per repeat in the STs [8] could be the core of a RiPP producing a Ser/Thr CPA (xxS/TxY, Tyr-phenol-O-to-C). These authors named these CPA peptides after the STs, where the amino acids Ser or Thr are typically located two positions before the Tyr residue, which is the only fully conserved amino acid in the pentapeptide. Unfortunately, to date, no Ser/Thr CPAs have been isolated as plant natural products [16]. BURP-domain peptide cyclases are involved in the processing of RiPPs [17]. Interestingly, we observed that proteins containing the BURP domain [20,62] appeared in the searches performed in this work using SST sequences, although, as defined, SST proteins lack the BURP domain. We found that these proteins displayed an N-terminal consensus sequence (Ax_6_YWx_7_PMP) [16] resembling the ST and SST common region described above. This coincidence could have a functional meaning or could be related to the subcellular localisation or to the formation of protein interactions.

To further investigate the role of SST proteins in plant physiology, we focused on a unique SST protein in *A. thaliana*, SST1, which is encoded by the gene At1g49310 (*SST1*). The genes surrounding a given one in the genome can help to infer certain features about the function or processing of the encoded protein [62]. The genes closest to *SST1* are *RABG3E*, which encodes a RAB GTPase involved in vesicle trafficking in the vacuolar system and related to protein transport in response to abiotic stress [63], and *USPL1*, which encodes a BURP domain-containing protein, which is also related to stress and might be associated with the formation of protein storage vacuoles [64,65] (Figure 8). Furthermore, *USPL1* is the main gene co-expressed with *SST1,* as predicted by ATTEDII [59]; consequently, a functional relationship might exist between these two proteins. Additionally, we found two other root-related genes highly co-expressed with *SST1*: *PSY3*, encoding a small signalling peptide mainly expressed in roots and upregulated under some abiotic stresses [66], and *PHO1*, which is involved in the transfer of phosphate from root epidermal and cortical cells to the xylem [67]. Thus, these analyses suggest that SST1 could be involved in some processes during abiotic stress in roots, which could be related to the response to cold and/or hypoxia as revealed by transcriptomic analysis [68]. This genomic co-localisation of the *BURP* and *SST*/*ST* genes has also been found in *M. truncatula* (Figure S4). For example, the genetic environment of *MtSST2* (Medtr3g116395) is enriched in BURP-encoding genes (Medtr3g116320, Medtr3g116380, and Medtr3g116419), which are also co-expressed with genes that are activated in roots. Also, *MtSST2* is located near the *MtST3* and *MtST2* genes (Medtr3g116430 and Medtr3g116440, respectively) which are upregulated in the presence of ABA and have been linked to the abiotic stress response [9]. When the precursors of RiPPs do not have a BURP domain themselves, another protein with this domain is necessary for their processing (split pathway) [16,17]. The genes encoding both proteins are usually co-clustered in the genome and are transcriptionally co-regulated [16,17], which could be the case for SST1 and USPL1 and other PF10950 proteins.

The SST1 protein seems to act preferentially in roots, as the activity of pSST1 is higher in this organ (Figure 9), and transcripts are more abundant (Figure 10), which coincides with the accumulation of legume *ST* transcripts [2,3,4,8,11]. Also, CPAs are usually isolated from roots, possibly because it is the primary accumulation organ [18]. In silico analysis of the *SST1* expression profile based on RNA-seq and microarray studies compiled in databases [68,69] also indicated this root-associated accumulation. The lack of pSST1 activity in the root apex correlates with the absence of *SST1* transcripts in this area [69,70]. Also, our results are consistent with a significant expression of the *SST1* gene in the stele, especially in the procambial cells in the root maturation zone [71]. Indeed, in this study the protein is detected in the root vascular cambium (Figure 12).

Despite the main presence of *SST1* in roots, transcripts also accumulate to some extent in above-ground organs (Figure 10). Other studies have detected transcripts in internodes, which are higher in older internodes (Figure 10) [69], similar to the accumulation of transcripts in some legumes [2,4]. Regarding the reproductive organs, it is noteworthy that there is high mRNA accumulation in mature siliques (Figure 10), which could be due to the high transcript levels found in the developing seeds [68,72]. However, no promoter activity is detected (Figure 9D) and the same occurs in flowers and floral buds (Figure 9D and Figure 10). Consistent with our results (Figure 10), a higher transcript accumulation in flowers than in flower buds has been reported [68,72]. Nevertheless, the results in the reproductive parts are not consistent across all platforms, and in some analyses no *SST1* transcripts are detected in flowers, nor during seed and silique development [70], coinciding in this case with promoter activity (Figure 9D).

Notably, the activity of pSST1 is restricted to specific points in the aerial organs, the serrated margin primordia, and the hydathodes (Figure 9(A2,B2,C2)), which explains the low accumulation of transcripts in the aerial organs of the seedlings (Figure 10). These results suggest the involvement of IAA in *SST1* activation and might be associated with the accumulation of *SST1* mRNA in meristematic areas, such as the procambium of the cauline apex [73,74] and the shoot apex meristem [71].

We raised antibodies against SST1 to localise the protein in planta. Despite specifically recognising recombinant SST1 produced in *E. coli*, no signal was detected against protein extracts from Arabidopsis, either WT or overexpressing lines (Figure 11A,C). Similarly, anti-SST1 did not recognise the protein extracted from the heterologous system of *N. bentamiana* leaves (Figure 11A,C). This lack of recognition may be due to masking caused by post-translational processing such as glycosylation or phosphorylation. However, after an in silico analysis in ScanProsite [39], no specific site of post-translational modifications was determined that could explain this. Another possible explanation is that in the tertiary structure of the protein, the epitopes remain inside the structure and are not accessible to antibodies. Finally, it is possible that the SST protein was processed by proteolytic cleavage in plants, which does not occur in bacteria, resulting in small fragments that cannot be detected by electrophoresis despite using a Tris-Tricine system suitable for small peptides [56]. Otherwise, the anti-SST1 antibodies recognise the SST1 protein fused to GFP (Figure 11B,D), so we can hypothesise that the binding of the protein to GFP avoids the post-translational changes that prevent us from detecting it in its native state.

In silico predictions regarding SST proteins’ subcellular localisation suggest extracellular protein sorting (Table S1) with their entry into the ER being determined by the SP that is encoded in the SST protein. However, additional targeting signals to other organelles are diverse and can be located at both the N- and C-terminal ends of the proteins. Furthermore, the signal for vacuolar targeting typically relies on the attainment of a specific three-dimensional conformation rather than the presence of a consensus sequence [75,76]. Our results showed that small variations at the sequence level lead to different predictions (Table S1), and dual subcellular localisation, either inside the cell or in the apoplast, has been observed for some ST proteins, such as CaST1 and CaST2 [7] or MtST2, MtST3, and MtST6 [9,10]. Consequently, it was necessary to empirically establish the subcellular localisation of SSTs, which is crucial to find the possible function of these proteins. With this aim, we studied the Arabidopsis SST1 protein, which is described as a transmembrane protein in the TAIR database [77], despite not having any other transmembrane domain apart from the SP.

The SST1 protein fused to GFP at its N- and C-terminal ends (Figure 13A,B) was detected inside the cells, in the cytoplasm but also in specific granules, unlike ST proteins which accumulate in the apoplast [7,9,10]. Each of the fusion proteins produced a very similar pattern of GFP accumulation, for which we consider two competing explanations. The first is that the SST1 protein is not proteolytically processed or is stored in an unprocessed state; hence, the protein accumulates in the same structure irrespective of the presence of GFP at the C- or the N-terminus. The second is that the protein undergoes proteolytic processing at both ends upon synthesis and that the GFP is bound to the discarded part and accumulates in granules before being degraded by the cell machinery.

One of our objectives was to determine the nature of the granules in which GFP located. We discarded the nucleus as the sequence lacked a nuclear localisation signal [78], and there were several granules within the same cell (Figure 13). To determine in which organelle GFP-bound proteins accumulated, we analysed double-transgenic Arabidopsis plants carrying SST1 plus GFP translational fusions and CFP-bound organelle marker proteins [50]. The green and cyan fluorescence partially overlapped in the plants carrying ER (Figure 14) and vacuole (Figure 15) markers, but they remained separated in the Golgi-tagged CFP plants (Figure S8). Recently, several proteins have been reported to travel through the endomembrane system bypassing the Golgi apparatus in the Unconventional Protein Secretion pathway [79]. Therefore, we propose that SST1 enters the ER and then travels to the vacuole for processing or storage. Also, the granules observed in SST1 and GFP protein fusions resemble precursor-accumulating vesicles (PAC) where storage proteins are kept before reaching the lytic vacuole, showing ER-derived membranes [80]. These proteins, initially produced in the ER, form aggregates that somehow trigger their accumulation in the PAC vesicles. Alternatively, it is possible that the SST1 protein is processed in the ER and only the remaining GFP-bound amino acid sequence is sent to the vacuole for degradation, suggesting that the aggregates may be related to autophagic processes [79]. The observed subcellular localisation of SST1 is consistent with the hypothesis that the protein would be a CPA and would undergo proteolytic processing, which typically occurs in the vacuole, to produce the core pentapeptide. However, to confirm the GFP-tagged subcellular localisation of SST1 and to properly interpret these results regarding the putative proteolytic processing, future research is required.

Considering our results and previous studies, our hypothesis about the function of PF10950 proteins, ST and SST, is that they are, respectively, multi-core or single-core precursor proteins for CPA produced by BURP-domain peptide cyclases via the split burpitide pathway (CPAB). Future work would be necessary to test this theory and determine whether the BURP proteins whose genes co-localise with the ST and SST proteins in the genome are able to cycle the xxxxY pentapeptide, since the Ser/Thr CPAs have not been isolated free from plants. If they were confirmed as RIPPs, they could produce bioactive molecules with interesting biotechnological properties.

## 5. Conclusions

This work sheds light on the unknown PF10950 family of plant proteins. The SST proteins are a new type of PF10950 proteins that, unlike the previously characterised ST proteins within this family, do not have tandem repeats and therefore are much smaller. These proteins are found in all eudicots, with the exception of the less-evolved ones, including families such as cruciferous, in which ST proteins are not found. SST proteins have an SP and two conserved regions, while the N-terminal region is conserved among all SSTs; the C-terminal region is similar to the ST consensus repeat. These two conserved SST regions may constitute the minimal functional architecture of DUF2775. Considering the Arabidopsis SST1 protein as a model, it seems clear that these proteins could perform their main function in roots, and they might be post-translationally processed, possibly in the ER and/or the vacuole. This processing is consistent with the hypothesis that PF10950 proteins could lead to CPAB cyclopeptides. Future research will focus on testing this hypothesis, by assessing the subcellular sorting of SST1 and ascertaining if the protein undergoes any processing.

## Data Availability

All the data generated or analysed during this study are included in this published article and its additional information files.

## Supplementary Materials

The following supporting information can be downloaded at https://www.mdpi.com/article/10.3390/plants14071117/s1, Figure S1: Dendrogram of mature SST sequences belonging to the group of *Other* families; Figure S2: WebLogo of mature SST protein sequences without scaled stack width; Figure S3: WebLogos of the non-repeat zone of the *Medicago truncatula* STs and SSTs; Figure S4: Genetic environment of the 4 *MtSSTs* and 6 *MtSTs* genes of *M. truncatula*; Figure S5: GUS activity in 5 d old pSST1::GUS etiolated transgenic seedlings; Figure S6: Representative agarose (2%) gel electrophoresis of sqRT-PCR products; Figure S7: Heterologous production of mSST1 protein; Figure S8: Subcellular localisation of *A. thaliana* SST1 fused to GFP and Golgi apparatus cyan marker; List S1: List of species analysed to find SST sequences; List S2: List of the 194 SST protein sequences in FASTA; List S3: List of the 194 SST mature protein sequences in FASTA; List S4: List of 151 SST mature protein sequences used in sequence comparison in FASTA; Table S1: SST genes and proteins found in different species and their main features.

## Abbreviations

The following abbreviations are used in this manuscript:

AM	Arbuscular mycorrhizal
CPA	Cyclopeptide alkaloid
CPAB	Cyclopeptide alkaloid-type burpitide
DUF	Domain of unknown function
gSST1	Promoter plus transcriptional unit without 3’ UTR of Arabidopsis SST1
mSST1	Mature AtSST1 after the processing of the signal peptide
PAC	Precursor accumulating vesicles
PERK	Proline-rich extensin-like receptor kinase
PF	Protein family
PHO1	Phosphate transporter
pSST1	Promoter of Arabidopsis *SST1* gene
PSY3	Tyrosine-sulphated protein of unknown function
RABG3E	Ras-related RAB-7A protein
RiPP	Ribosomally synthesised and post-translationally modified peptides
SP	Signal peptide
SST	Short specific tissue
SST1	The single SST of *Arabidopsis thaliana*
ST	Specific tissue
USPL1	Unknown seed protein like 1

## References

[1] Classification of Protein Families. https://www.ebi.ac.uk/interpro/entry/pfam/#table.

[2] De Vries S.C., De Vos W.M., De Harmsen M.C., Wessels J.G.H. (1985). A shoot-specific mRNA from pea: Nucleotide sequence and regulation as compared to light-induced mRNAs. Plant Mol. Biol..

[3] Williams M.E., Mundy J., Kay S.A., Chua N.-H. (1990). Differential expression of two related organ-specific genes in pea. Plant Mol. Biol..

[4] Muñoz F.J., Dopico B., Labrador E. (1997). Two growth-related organ-specific cDNAs from *Cicer arietinum* epicotyls. Plant Mol. Biol..

[5] Hernández-Nistal J., Labrador E., Martín I., Jiménez T., Dopico B. (2006). Transcriptional profiling of cell wall protein genes in chickpea embryonic axes during germination and growth. Plant Physiol. Biochem..

[6] Hernández-Nistal J., Martín I., Esteban R., Dopico B., Labrador E. (2010). Abscisic acid delays chickpea germination by inhibiting water uptake and downregulating genes encoding cell wall remodelling proteins. Plant Growth Regul..

[7] Albornos L., Cabrera J., Hernández-Nistal J., Martín I., Labrador E., Dopico B. (2014). Organ accumulation and subcellular location of *Cicer arietinum* ST1 protein. Plant Sci..

[8] Albornos L., Martín I., Iglesias R., Jiménez T., Labrador E., Dopico B. (2012). ST proteins, a new family of plant tandem repeat proteins with a DUF2775 domain mainly found in *Fabaceae* and *Asteraceae*. BMC Plant Biol..

[9] Albornos L., Martín I., Labrador E., Dopico B. (2017). Three members of *Medicago truncatula* ST family are ubiquitous during development and modulated by nutritional status (MtST1) and dehydration (MtST2 and MtST3). BMC Plant Biol..

[10] Albornos L., Martín I., Hernández-Nistal J., Labrador E., Dopico B. (2018). Three members of *Medicago truncatula* ST family (MtST4, MtST5 and MtST6) are specifically induced by hormones involved in biotic interactions. Plant Physiol. Biochem..

[11] Albornos L., Martín I., Hernández-Nistal J., Labrador E., Dopico B. (2019). Promoter activity of genes encoding the Specific Tissue protein family in the reproductive organs of *Medicago truncatula*. Biol. Plant..

[12] Albornos L., Casado-del-Castillo V., Martín I., Díaz-Mínguez J.M., Labrador E., Dopico B. (2021). Specific tissue proteins 1 and 6 are involved in root biology during normal development and under symbiotic and pathogenic interactions in *Medicago truncatula*. Planta.

[13] Wechter W.P., Levi A., Harris K.R., Davis A.R., Fei Z., Katzir N., Giovannoni J.J., Salman-Minkov A., Hernandez A., Thimmapuram J. (2008). Gene expression in developing watermelon fruit. BMC Genom..

[14] Gaude N., Bortfeld S., Duensing N., Lohse M., Krajinski F. (2012). Arbuscule-containing and non-colonized cortical cells of mycorrhizal roots undergo extensive and specific reprogramming during arbuscular mycorrhizal development. Plant J..

[15] Liu J., Maldonado-Mendoza I., Lopez-Meyer M., Cheung F., Town C.D., Harrison M.J. (2007). Arbuscular mycorrhizal symbiosis is accompanied by local and systemic alterations in gene expression and an increase in disease resistance in the shoots. Plant J..

[16] Lima S.T., Ampolini B.G., Underwood E.B., Graf T.N., Earp C.E., Khedi I.C., Pasquale M.A., Chekan J.R. (2023). A widely distributed biosynthetic cassette is responsible for diverse plant side chain cross-linked cyclopeptides. Chem. Int. Ed..

[17] Chekan J.R., Mydy L.S., Pasquale M.A., Kersten R.D. (2024). Plant peptides—Redefining an area of ribosomally synthesized and post-translationally modified peptides. Nat. Prod. Rep..

[18] Tan N.-H., Zhou J. (2006). Plant cyclopeptides. Chem. Rev..

[19] BLAST in TAIR Database. https://www.arabidopsis.org/Blast/index.jsp.

[20] Hattori J., Boutilier K.A., Campagne M.M.V., Miki B.L. (1998). A conserved BURP domain defines a novel group of plant proteins with unusual primary structures. Mol. Gen. Genet..

[21] Basic Local Alignment Search Tool (BLAST). https://blast.ncbi.nlm.nih.gov/Blast.cgi.

[22] Phytozome 13 The Plants Genomics Resource. https://phytozome-next.jgi.doe.gov/.

[23] Goodstein D.M., Shu S., Howson R., Neupane R., Hayes R.D., Fazo J., Mitros T., Dirks W., Hellsten U., Putnam N. (2012). Phytozome: A comparative platform for green plant genomics. Nucleic Acids Res..

[24] Common Tree Taxonomy Tool, NCBI. http://www.ncbi.nlm.nih.gov/Taxonomy/CommonTree/wwwcmt.cgi.

[25] Molecular Evolution, Phylogenetics and Epidemiology, FigTree v1.3.1. http://tree.bio.ed.ac.uk/software/figtree/.

[26] International Plant Names Index, IPNI. https://www.ipni.org/.

[27] Expasy Translate Tool. https://web.expasy.org/translate/.

[28] Technical University of Denmark (DTU), Department of Health Technology, SignalP-5.0. https://services.healthtech.dtu.dk/service.php?SignalP-5.0.

[29] Petersen T.N., Brunak S., von Heijne G., Nielsen H. (2011). Discriminating signal peptide from transmembrane regions. Nat. Methods.

[30] Technical University of Denmark (DTU), Department of Health Technology, SignalP-6.0. https://services.healthtech.dtu.dk/service.php?SignalP-6.0.

[31] Teufel F., Almagro-Armenteros J.J., Johansen A.R., Gíslason M.H., Pihl S.I., Tsirigos K.D., Winther O., Brunak S., Von Heijne G., Nielsen H. (2022). SignalP 6.0 predicts all five types of signal peptides using protein language models. Nat. Biotechnol..

[32] Technical University of Denmark (DTU), Department of Health Technology, TargetP-2.0. https://services.healthtech.dtu.dk/service.php?TargetP-2.0.

[33] Almagro-Armenteros J.J., Salvatore M., Winther O., Emanuelsson O., von Heijne G., Elofsson A., Nielsen H. (2019). Detecting Sequence Signals in Targeting Peptides Using Deep Learning. Life Sci. Alliance.

[34] Technical University of Denmark (DTU), Department of Health Technology, DeepLoc-2.0. https://services.healthtech.dtu.dk/service.php?DeepLoc-2.0.

[35] Thumuluri V., Almagro-Armenteros J.J., Johansen A.R., Nielsen H., Winther O. (2022). DeepLoc 2.0: Multi-label subcellular localization prediction using protein language models. Nucleic Acids Res..

[36] Technical University of Denmark (DTU), DeepTMHMM. https://dtu.biolib.com/DeepTMHMM.

[37] Hallgren J., Tsirigos K.D., Pedersen M.D., Almagro-Armenteros J.J., Marcatili P., Nielsen H., Krogh A., Winther O. (2022). DeepTMHMM predicts alpha and beta transmembrane proteins using deep neural networks. bioRxiv.

[38] Expasy, Compute pI/Mw. https://web.expasy.org/compute_pi.

[39] Prosite, ScanProsite Tool. https://prosite.expasy.org/scanprosite/.

[40] Protein Subcellular Localization Prediction WoLF PSORT. https://wolfpsort.hgc.jp/.

[41] EMBL European Bioinformatic Institute, EMBOSS. https://www.ebi.ac.uk/jdispatcher/sfc/emboss_seqret.

[42] Rice P., Longden I., Bleasby A. (2000). EMBOSS: The European Molecular Biology Open Software Suite. Trends Genet..

[43] Clustal Omega Multiple Sequence Alignment. https://www.ebi.ac.uk/jdispatcher/msa/clustalo.

[44] Sievers F., Wilm A., Dineen D., Gibson T.J., Karplus K., Li W., Lopez R., McWilliam H., Remmert M., Söding J. (2011). Fast, scalable generation of high-quality protein multiple sequence alignments using Clustal Omega. Mol. Syst. Biol..

[45] Madeira F., Pearce M., Tivey A.R.N., Basutkar P., Lee J., Edbali O., Madhusoodanan N., Kolesnikov A., Lopez R. (2022). Search and sequence analysis tools services from EMBL-EBI in 2022. Nucleic Acids Res..

[46] Waterhouse A.M., Procter J.B., Martin D.M.A., Clamp M., Barton G.J. (2009). Jalview Version 2—A multiple sequence alignment editor and analysis workbench. Bioinformatics.

[47] WebLogo 3, Generation of Sequence Logo. https://weblogo.threeplusone.com.

[48] Crooks G.E., Hon G., Chandonia J.M., Brenner S.E. (2004). WebLogo: A sequence logo generator. Genome Res..

[49] Clough S.J., Bent A.F. (1998). Floral dip: A simplified method for *Agrobacterium*-mediated transformation of *Arabidopsis thaliana*. Plant J..

[50] Nelson B.K., Cai X., Nebenführ A. (2007). A multicolored set of in vivo organelle markers for co-localization studies in Arabidopsis and other plants. Plant J..

[51] Sainsbury F., Thuenemann E.C., Lomonossoff G.P. (2009). PEAQ: Versatile expression vectors for easy and quick transient expression of heterologous proteins in plants. Plant Biotechnol. J..

[52] Izquierdo L., Martín I., Albornos L., Hernández-Nistal J., Hueso P., Dopico B., Labrador E. (2018). Overexpression of *Cicer arietinum* βIII-Gal but not βIV-Gal in arabidopsis causes a reduction of cell wall β-(1,4)-galactan compensated by an increase in homogalacturonan. J. Plant Physiol..

[53] Martín I., Jiménez T., Hernández-Nistal J., Dopico B., Labrador E. (2011). The βI-galactosidase of *Cicer arietinum* is located in thickened cell walls such as those of collenchyma, sclerenchyma and vascular tissue. Plant Biol..

[54] Zeitler B., Bernhard A., Meyer H., Sattler M., Koop H.U., Lindermayr C. (2013). Production of a de-novo designed antimicrobial peptide in *Nicotiana benthamiana*. Plant Mol. Biol..

[55] Martín I., Jiménez T., Esteban R., Dopico B., Labrador E. (2008). Immunolocalization of a cell wall β-galactosidase reveals its developmentally regulated expression in *Cicer arietinum* and its relationship to vascular tissue. J. Plant Growth Reg..

[56] Schägger H. (2006). Tricine-SDS-page. Nat. Protoc..

[57] JBrowse, the Next-Generation Genome Browser. https://jbrowse.org/jb2/.

[58] Diesh C., Stevens G.J., Xie P., Martinez T.D.J., Hershberg E.A., Leung A., Guo E., Dider S., Zhang J., Bridge C. (2023). JBrowse 2: A modular genome browser with views of synteny and structural variation. Genome Biol..

[59] Obayashi T., Hibara H., Kagaya Y., Aoki Y., Kinoshita K. (2022). ATTED-II v11: A plant gene coexpression database using a sample balancing technique by subagging of principal components. Plant Cell Physiol..

[60] Dehors J., Mareck A., Kiefer-Meyer M.-C., Menu-Bouaouiche L., Lehner A., Mollet J.-C. (2019). Evolution of cell wall polymers in tip-growing land plant gametophytes: Composition, distribution, functional aspects and their remodeling. Front. Plant Sci..

[61] Chase M.W., Christenhusz M.J.M., Fay M.F., Byng J.W., Judd W.S., Soltis D.E., Mabberley D.J., Sennikov A.N., Soltis P.S., Stevens P.F. (2016). An update of the Angiosperm Phylogeny Group classification for the orders and families of flowering plants: APG IV. Bot. J. Linnean Soc..

[62] Kersten R.D., Weng J.-K. (2018). Gene-guided discovery and engineering of branched cyclic peptides in plants. Proc. Natl. Acad. Sci. USA.

[63] Mazel A., Leshem Y., Tiwari B.S., Levine A. (2004). Induction of Salt and Osmotic Stress Tolerance by Overexpression of an Intracellular Vesicle Trafficking Protein AtRab7 (AtRabG3e). Plant Physiol..

[64] Harshavardhan V.T., Van Son L., Seiler C., Junker A., Weigelt-Fischer K., Klukas C., Altmann T., Sreenivasulu N., Bäumlein H., Kuhlmann M. (2014). AtRD22 and AtUSPL1, members of the plant-specific burp domain family involved in *Arabidopsis thaliana* drought tolerance. PLoS ONE.

[65] Ding X., Hou X., Xie K., Xiong L. (2009). Genome-wide identification of BURP domain containing genes in rice reveals a gene family with diverse structures and responses to abiotic stresses. Planta.

[66] Tost A.S., Kristensen A., Olsen L.I., Axelsen K.B., Fuglsang A.T. (2021). The PSY peptide family-expression, modification, and physiological implications. Genes.

[67] Reis R.S., Deforges J., Sokoloff T., Poirier Y. (2020). Modulation of shoot phosphate level and growth by PHOSPHATE1 upstream open reading frame. Plant Physiol..

[68] Winter D., Vinegar B., Nahal H., Ammar R., Wilson G.V., Provart N.J. (2007). An “Electronic Fluorescent Pictograph” browser for exploring and analyzing large-scale biological data sets. PLoS ONE.

[69] Klepikova A.V., Kasianov A.S., Gerasimov E.S., Logacheva M.D., Penin A.A. (2016). A high resolution map of the *Arabidopsis thaliana* developmental transcriptome based on RNA-seq profiling. Plant J..

[70] Transcriptome Variation Analysis (TraVA Database). http://travadb.org/.

[71] Brady S.M., Orlando D.A., Lee J.Y., Wang J.Y., Koch J., Dinneny J.R., Mace D., Ohler U., Benfey P.N. (2007). A high-resolution root spatiotemporal map reveals dominant expression patterns. Science.

[72] Schmid M., Davison T.S., Henz S.R., Pape U.J., Demar M., Vingron M., Schölkopf B., Weigel D., Lohmann J.U. (2005). A gene expression map of *Arabidopsis thaliana* development. Nat. Genet..

[73] Dinneny J.R., Long T.A., Wang J.Y., Jung J.W., Mace D., Pointer S., Barron C., Brady S.M., Schiefelbein J., Benfey P.N. (2008). Cell identity mediates the response of *Arabidopsis* roots to abiotic stress. Science.

[74] Tian C., Zhang X., He J., Yu H., Wang Y., Shi B., Han Y., Wang G., Feng X., Zhang C. (2014). An organ boundary-enriched gene regulatory network uncovers regulatory hierarchies underlying axillary meristem initiation. Mol. Syst. Biol..

[75] Neuhaus J.M., Rogers J.C. (1998). Sorting of proteins to vacuoles in plant cells. Plant Mol. Biol..

[76] Zhang X., Li H., Lu H., Hwang I. (2021). The trafficking machinery of lytic and protein storage vacuoles: How much is shared and how much is distinct?. J. Exp. Bot..

[77] The Arabidopsis Information Resource (TAIR). https://www.arabidopsis.org/.

[78] Lange A., Mills R.E., Lange C.J., Stewart M., Devine S.E., Corbett A.H. (2007). Classical Nuclear Localization Signals: Definition, Function, and Interaction with Importin α. J. Biol. Chem..

[79] Bellucci M., De Marchis F., Pompa A. (2018). The endoplasmic reticulum is a hub to sort proteins toward unconventional traffic pathways and endosymbiotic organelles. J. Exp. Bot..

[80] Hara-Nishimura I., Shimada T., Hatano K., Takeuchi Y., Nishimura M. (1998). Transport of storage proteins to protein storage vacuoles is mediated by large precursor-accumulating vesicles. Plant Cell.

