# Supplementary material for: A Comprehensive Analysis of Short Specific Tissue (SST) Proteins, a New Group of Proteins from PF10950 That May Give Rise to Cyclopeptide Alkaloids
Source: Plants (Basel). 2025 Apr 3;14(7):1117. doi: 10.3390/plants14071117 (PMC11991032; doi:10.3390/plants14071117)
Supplement: Supplementary file 1 [file plants-14-01117-s001.zip › Figure S1.pptx]

## Slide 1
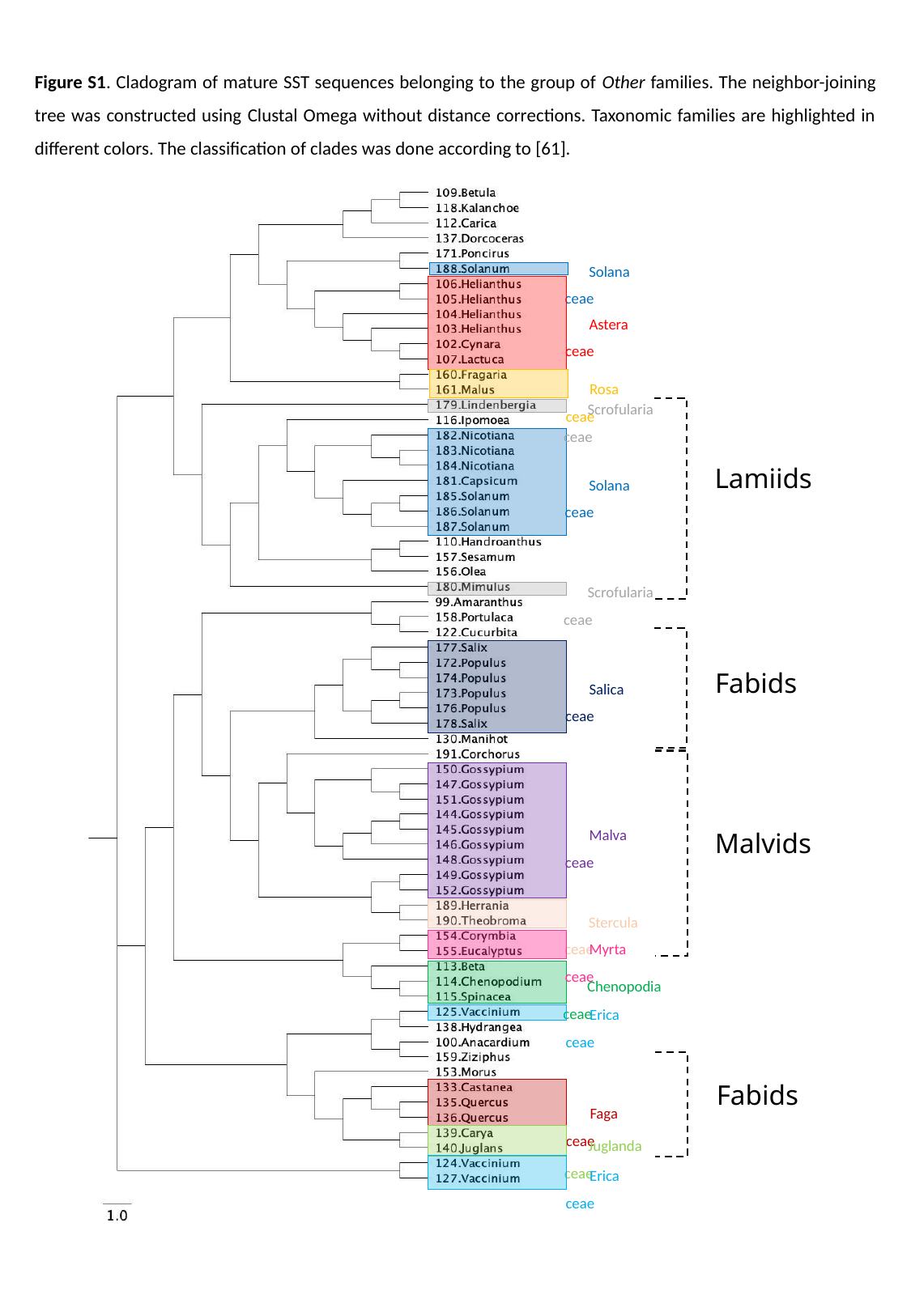

Figure S1. Cladogram of mature SST sequences belonging to the group of Other families. The neighbor-joining tree was constructed using Clustal Omega without distance corrections. Taxonomic families are highlighted in different colors. The classification of clades was done according to [61].
Solanaceae
Asteraceae
Rosaceae
Scrofulariaceae
Lamiids
Solanaceae
Scrofulariaceae
Fabids
Salicaceae
Malvaceae
Malvids
Sterculaceae
Myrtaceae
Chenopodiaceae
Ericaceae
Fabids
Fagaceae
Juglandaceae
Ericaceae
