# Supplementary material for: A Comprehensive Analysis of Short Specific Tissue (SST) Proteins, a New Group of Proteins from PF10950 That May Give Rise to Cyclopeptide Alkaloids
Source: Plants (Basel). 2025 Apr 3;14(7):1117. doi: 10.3390/plants14071117 (PMC11991032; doi:10.3390/plants14071117)

Figure S3. Separated WebLogos of the alignment of the non-repeats zone of the STs (top) and SSTs (bottom) of *Medicago truncatula* with scaled stack width. The use of WebLogo with scaled stack width give the letters of different width depending on the numbers of sequences that have a given letter. The overall height of the stack indicates the sequence conservation at that position, while the height of the symbols within the stack indicates the relative frequency of each amino acid at that position. Amino acids are colored-coded for clarity: D and E are in red; Y, W and F in green; L, R and H in blue; A, V, L, I, G and M in purple; and K, S, T, N, Q, C and P in black. Bit: a measure of conservation at a particular sequence position; the maximum conservation for a given amino acid in a sequence is 4.32 bits.

ST non-repeats zone

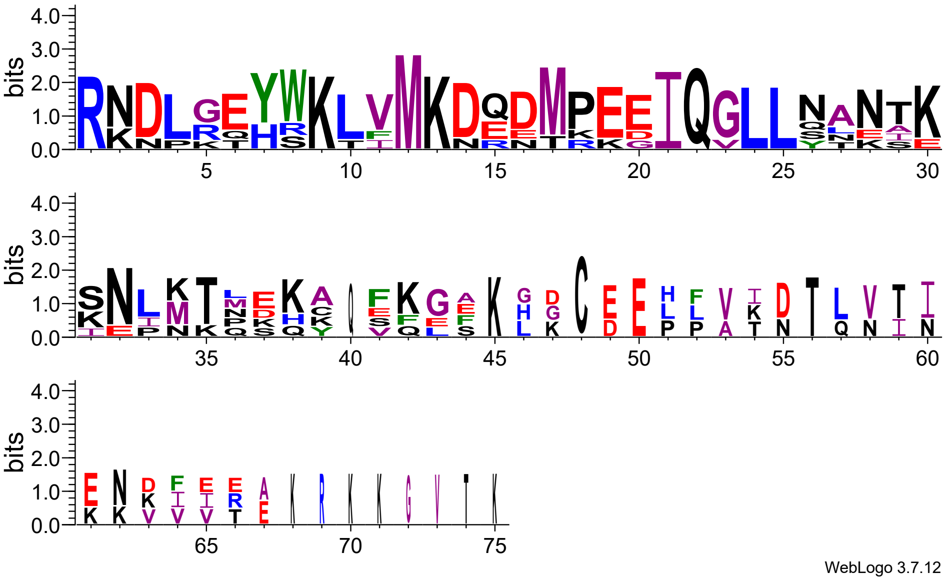

SST

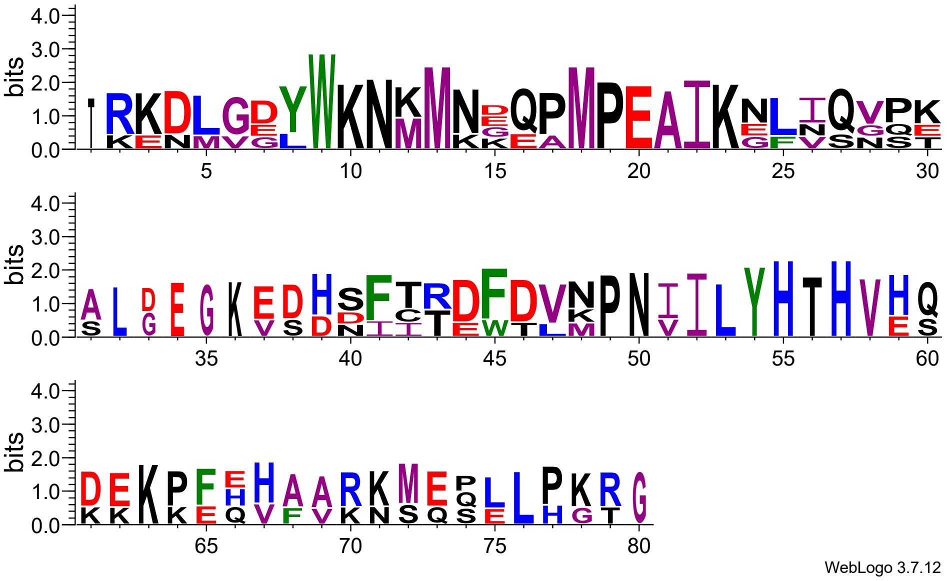

Supplement: Supplementary file 1 [file plants-14-01117-s001.zip › Figure S3.pdf]
