# Supplementary material for: A Comprehensive Analysis of Short Specific Tissue (SST) Proteins, a New Group of Proteins from PF10950 That May Give Rise to Cyclopeptide Alkaloids
Source: Plants (Basel). 2025 Apr 3;14(7):1117. doi: 10.3390/plants14071117 (PMC11991032; doi:10.3390/plants14071117)
Supplement: Supplementary file 1 [file plants-14-01117-s001.zip › Figure S4.pdf]

Figure S4. Genetic environment of the 4 *MtSSTs* and 6 *MtSTs* genes of *Medicago truncatula* as determined using the JBrowse tool in the Phytozome database. Small fragments of *M. truncatula* chromosomes containing the genes encoding the SST and ST proteins of this species are highlighted in blue. Chromosome 3 contains the genes *MtSST1*, *MtSST2*, and *MtST2-6*; chromosome 4 contains *MtSST3* and *MtST1* and chromosome 5 contains *MtSST4*.

Medicago truncatula chromosome 3: MtrSST1 (Medtr3g007880)

Medtr3g007863 F-box domain **Putative** F-box/RNI/FBD-like domain protein	Medtr3g007864 Exostosin family protein-related **Putative** Acetylglucosaminyl transferase **Related to** xyloglucan galactosyl transferases	Medtr3g007865 Nicotinamide-nucleotide adenylyltransferase / NMNAT **Putative** Role in NAD biosynthetic process	Medtr3g007870 L-type lectin-domain containing receptor kinase S.7-related. Legume lectin domain putative carbohydrate binding	**Medtr3g007880 MtrSST1**	Medtr3g007900 Extensin, proline-rich protein **Putative** Giberellin regulated protein	Medtr3g007910 Predicted transporter (major facilitator superfamily) **Putative** Sugar/inositol transporter	Medtr3g007920 Weak chloroplast movement under blue light (WEMBL) **Putative** Paramyosin	Medtr3g007930 Actin patches distal protein 1 **Putative** sucrase/ferredoxin family protein	Medtr3g007950, 70 NAD dependent epimerase/dehydratase **Putative** phenylcoumaran benzylic ether reductase-like protein

Medicago truncatula chromosome 3: MtrST5 (Medtr3g034610) and MtrST4 (Medtr3g034640)

	Medtr3g034460, 430 and 380 Leucine-rich repeat-containing protein. **Putative** hydrolase of nucleoside triphosphates, ribonuclease inhibitor disease resistance protein with LRR and NB-ARC domains	Medtr3g034540 Hypothetical protein. Small protein	**Medtr3g034610 MtrST5**	**Medtr3g034640 MtrST4**	Medtr3g034650 Leucine-rich repeat-containing protein. **Putative** ribonuclease inhibitor, disease resistance protein with LRR and NB-ARC domains	Medtr3g034660 Seed maturation family protein. **Putative** LEA Coexpressed with genes in root specific subnetwork	Medtr3g034670 Chloroplastic pheophytinase alpha-beta-hydrolase	Medtr3g034675 Hypothetical protein. Small protein This gene is not expressed	Medtr3g034680 Zinc finger protein constans-like 5

Medicago truncatula chromosome 3: MtrST6 (Medtr3g107810)

Medtr3g107750 PIF-1 like helicase	Medtr3g107755 Hypothetical protein. Small protein	Medtr3g107770 Chitinase-related **Putative** expansin endoglucanase Coexpressed with genes in root specific subnetwork	Medtr3g107800 ABC transporter B family member 11-related **Putative** Steroid-transporting ATPase	**Medtr3g107810 MtrST6**	Medtr3g107820 Hypothetical protein. **Putative** Nucleic acid binding Small protein	Medtr3g107830 BURP domain	Medtr3g107860 Hypothetical protein.	Medtr3g107870 ATP-binding cassette transporter of plant PDR (pleiotropic drug resistance) family **Putative** Monosaccharide, antifungal transporter	Medtr3g107870 Protein phosphatase 2C 22-related Coexpressed with genes in root specific subnetwork

Medicago truncatula chromosome 3: MtrSST2 (Medtr3g116395), MtrST3 (Medtr3g116430) and MtrST2 (Medtr3g116440)

Medtr3g116320<br>BURP domain<br>**Putative**<br>Nucleic-acid binding	Medtr3g116380<br>BURP domain<br>Coexpressed with genes in root specific subnetwork	**Medtr3g116395**<br>**MtrSST2**	Medtr3g116410<br>BURP domain<br>**Putative**<br>Isopropylmalate Dehydrogenase<br>Coexpressed with genes in root specific subnetwork	**Medtr3g116430**<br>**MtrST3**	**Medtr3g116440**<br>**MtrST2**	Medtr3g116450<br>Proline-rich receptor-like protein kinase perk5-related	Medtr3g116460<br>Alpha,alpha-trehalose-phosphate synthase (UDP-forming) / UDP-glucose--glucose-phosphate glucosyltransferase<br>This gene is not expressed	Medtr3g116470<br>Not Defined<br>**Putative**<br>hydroxyproline-rich glycoprotein family protein<br>This gene is not expressed	Medtr3g116480<br>Coatomer subunit gamma<br>**Related to**<br>Intracellular protein transport and clathrins

Medicago truncatula chromosome 4: MtrSST3 (Medtr4g069770) and MtrST1 (Medtr4g069810)

Medtr4g069750<br>Hypothetical protein.<br>Small protein	Medtr4g069760<br>Hypothetical protein.<br>Small protein<br>This gene is not expressed	**Medtr4g069770**<br>**MtrSST3**<br>Coexpressed with genes in root specific subnetwork	Medtr4g069800<br>ATP-dependent CLP protease proteolytic subunit 6, chloroplastic<br>Coexpressed with genes in leaf specific subnetwork	**Medtr4g069810**<br>**MtrST1**	Medtr4g069820<br>Hypothetical protein.<br>**Putative**<br>Glycosidase<br>Small protein	Medtr4g069830<br>SWI/SNF complex subunit SWI3D<br>**Related to**<br>Chromatin remodelling, DNA binding<br>Coexpressed with genes in nodule specific subnetwork	Medtr4g069830<br>Glycosyltransferase family 1<br>**Related to**<br>Sugar transferase to glycogen, fructose-6-P and lipopolysaccharides	Medtr4g069830<br>RAS-related protein RABG3F<br>**Putative**<br>Small GTPase RAB7 group<br>**Related to**<br>protein cargo in vacuoles<br>Coexpressed with genes in root specific subnetwork	

Medicago truncatula chromosome 5: MtrSST4 (Medtr5g095980)

Medtr5g095950, 40<br>Exostosin family protein-related<br>**Putative**<br>Acetylglucosaminyl transferase<br>**related to**<br>xyloglucan galactosyl transferases	Medtr5g095955<br>Hypothetical protein.<br>Small protein	Medtr5g095960<br>Nicotinamide-nucleotide adenylyltransferase / NMNAT<br>**Putative**<br>Role in NAD biosynthetic process	Medtr5g095970<br>L-type lectin-domain containing receptor kinase S.7-related. Legume lectin domain putative carbohydrate binding	**Medtr5g095980**<br>**MtrSST4**	Medtr5g095990<br>Extensin, proline-rich protein<br>**Putative**<br>Giberellin regulated protein	Medtr5g096000<br>Predicted transporter (major facilitator superfamily)<br>**Putative**<br>Sugar/inositol transporter	Medtr5g096010<br>Hypothetical protein.<br>**Putative**<br>F-box<br>Small protein	Medtr5g096020<br>Ribosomal RNA methyltransferase NOP2-related<br>Coexpressed with genes in leaf specific subnetwork	Medtr5g096020<br>Cleavage site for pathogenic type III effector avirulence factor Avr (AvrRpt-cleavage)<br>Coexpressed with genes in root specific subnetwork
