# Supplementary material for: A Comprehensive Analysis of Short Specific Tissue (SST) Proteins, a New Group of Proteins from PF10950 That May Give Rise to Cyclopeptide Alkaloids
Source: Plants (Basel). 2025 Apr 3;14(7):1117. doi: 10.3390/plants14071117 (PMC11991032; doi:10.3390/plants14071117)
Supplement: Supplementary file 1 [file plants-14-01117-s001.zip › Figure S5.pptx]

## Slide 1
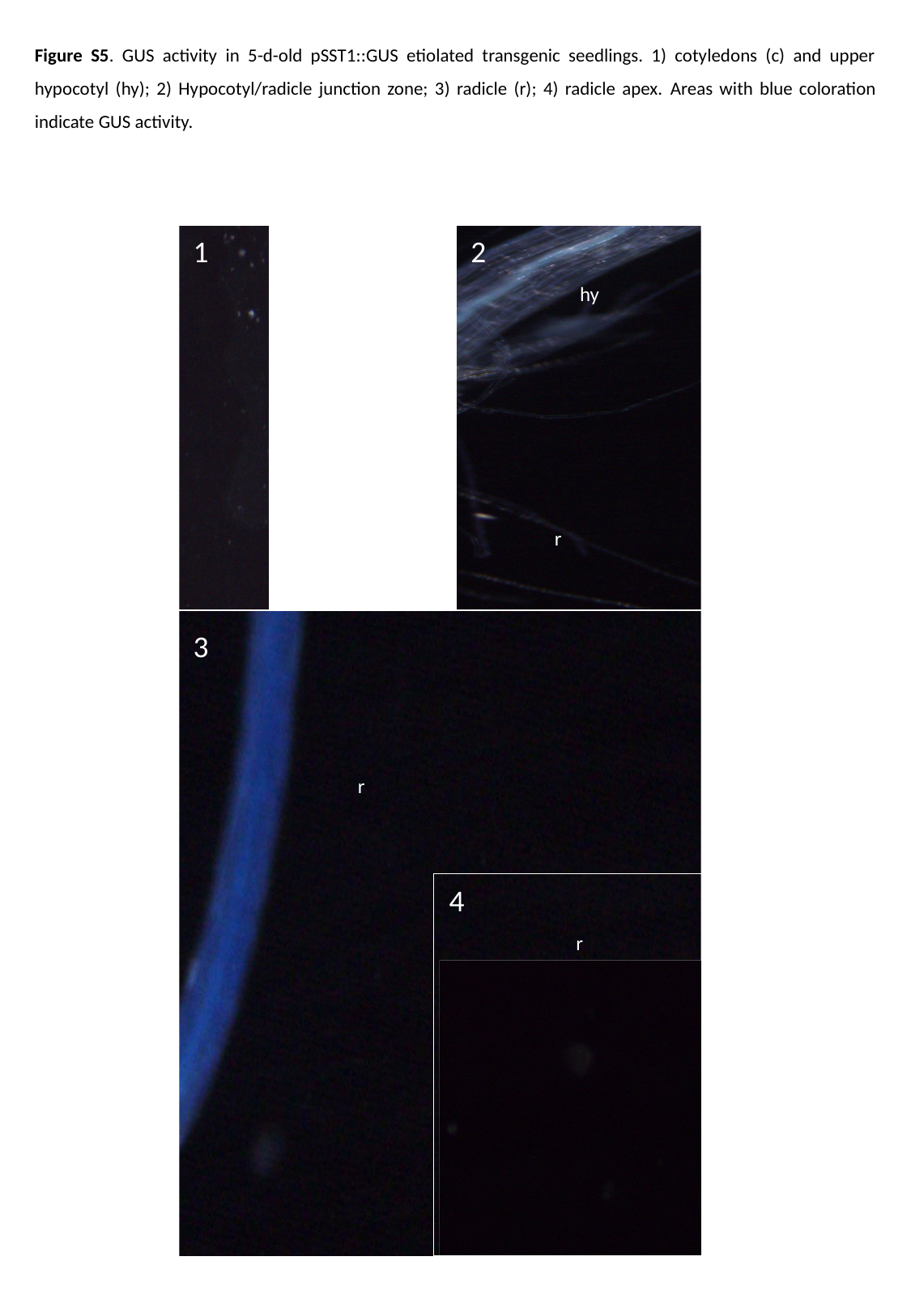

Figure S5. GUS activity in 5-d-old pSST1::GUS etiolated transgenic seedlings. 1) cotyledons (c) and upper hypocotyl (hy); 2) Hypocotyl/radicle junction zone; 3) radicle (r); 4) radicle apex. Areas with blue coloration indicate GUS activity.
1
2
c
hy
hy
r
3
r
4
r
