# Supplementary material for: A Comprehensive Analysis of Short Specific Tissue (SST) Proteins, a New Group of Proteins from PF10950 That May Give Rise to Cyclopeptide Alkaloids
Source: Plants (Basel). 2025 Apr 3;14(7):1117. doi: 10.3390/plants14071117 (PMC11991032; doi:10.3390/plants14071117)

Figure S6. Representative agarose (2%) gel electrophoresis of sqRT-PCR products. In the upper part of the gel, *SST1* amplicons obtained with the intron-spanning primer pair SST1.F/SST1.R giving an amplicon of 130 bp (210 bp with the intron). In the lower part of the gel, *ACT2* amplicons obtained with the intron-spanning primer pair Act2.F/Act2.R giving an amplicon of 200 bp (271 bp with the intron). Et5 and Et10, etiolated 5- and 10-d-old seedlings, respectively; R5 and A5, roots and aerial part of 5-d-old green seedlings; R10 and A10, roots and aerial part of 10-d-old green seedlings; R17, roots (R) of 17-d-old plants; L1, L2 and L3 first second and third pair of leaves of 17-d-old plants, respectively; IN1 and IN3 first and third internode of 32-d-old plants; FB, flower buds (FB); F, flowers; DS, developing siliques; MS, mature siliques of arabidopsis plants. M, markers hyperladder 1000 pb from Bioline. The lack of a band of 271 pb in the *ACT2* amplicon indicated that gDNA was properly eliminated using an extra DNase treatment. The only amplified 200 pb band was used as internal control. *SST1* 130 pb amplicon correspond to the mature RNA which is the one quantified and shown in figure 10. The upper band that appears in some organs around 200 pb is considered unprocessed RNA.

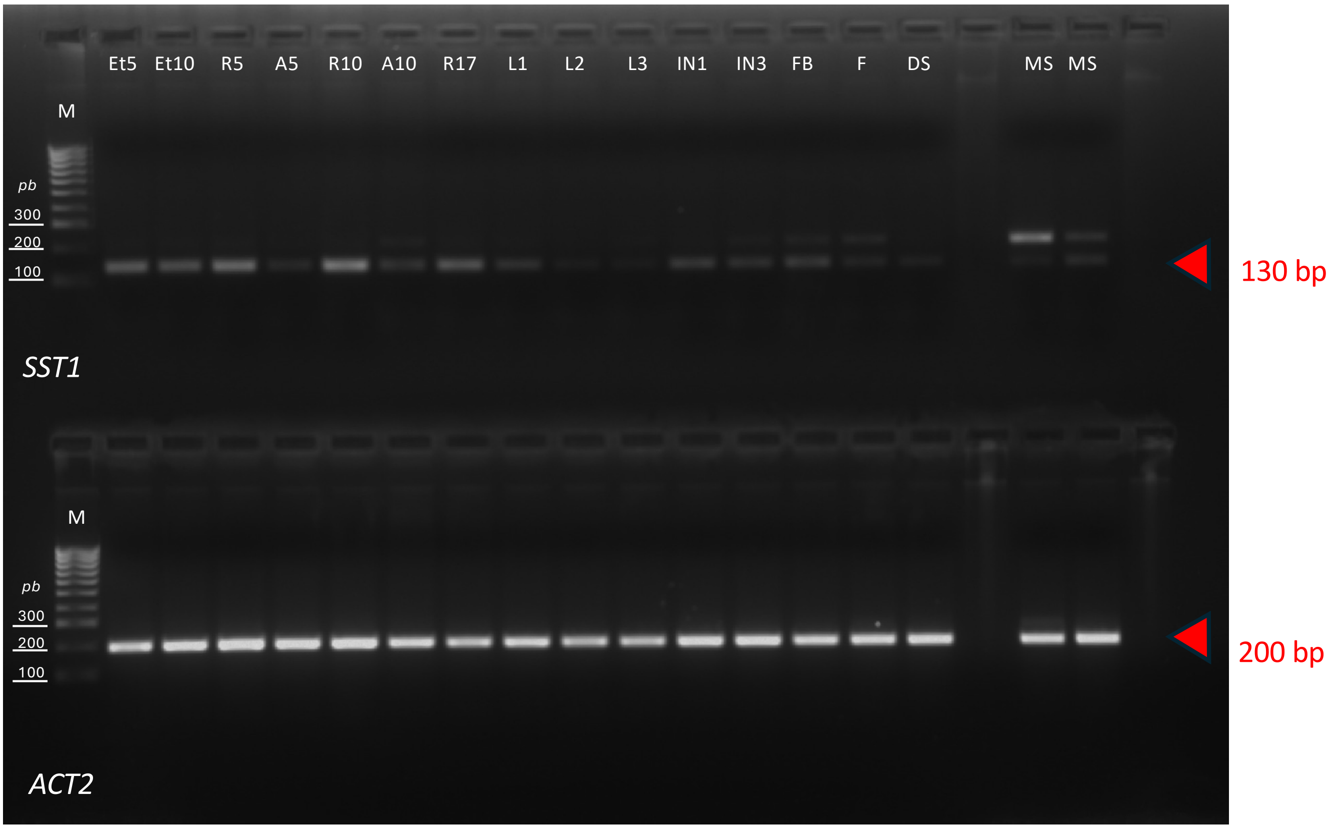

Supplement: Supplementary file 1 [file plants-14-01117-s001.zip › Figure S6.pdf]
