# Supplementary material for: A Comprehensive Analysis of Short Specific Tissue (SST) Proteins, a New Group of Proteins from PF10950 That May Give Rise to Cyclopeptide Alkaloids
Source: Plants (Basel). 2025 Apr 3;14(7):1117. doi: 10.3390/plants14071117 (PMC11991032; doi:10.3390/plants14071117)

Figure S7. Heterologous production of mSST1 protein in *Escherichia coli*. A) SDS-PAGE of protein extracts from *E. coli* strain BL21 in the untransformed control (C) and in the transformed colony 9 at 0, 1, 2, 3, 4 and 5 h (subscript from 0 to 5) after the IPTG induction. Red arrows indicate the extra band corresponding to the mature SST1 protein

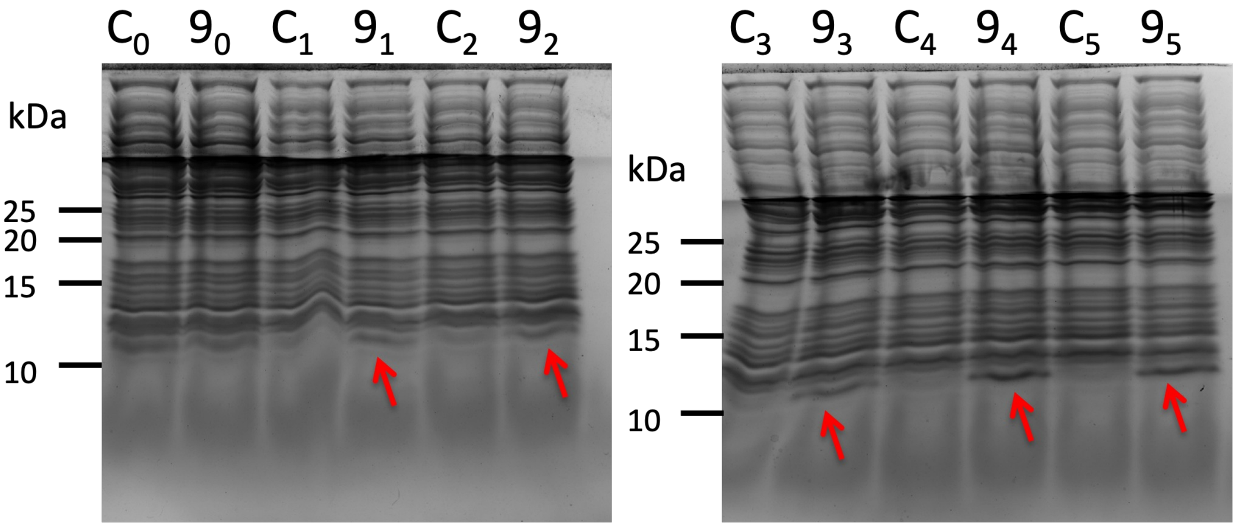

Supplement: Supplementary file 1 [file plants-14-01117-s001.zip › Figure S7.pdf]
