# Supplementary material for: A Comprehensive Analysis of Short Specific Tissue (SST) Proteins, a New Group of Proteins from PF10950 That May Give Rise to Cyclopeptide Alkaloids
Source: Plants (Basel). 2025 Apr 3;14(7):1117. doi: 10.3390/plants14071117 (PMC11991032; doi:10.3390/plants14071117)

Figure S8. Subcellular location of *Arabidopsis thaliana* SST1 fused to GFP and Golgi apparatus cyan marker. Representative confocal microscopy images of roots producing (A) the SST1 protein fused to GFP at the C-terminus or (B) at the N-terminus, together with a Golgi apparatus protein marker tagged with cyan fluorescent protein (G/CFP). The roots were mounted in water or in mannitol 0.6 M. Left to right columns display: green fluorescence from GFP fused to SST1; blue fluorescence from the Golgi protein marker; merged signal from the green and blue channels; 3D reconstruction with the projection of green and blue signals and one representative longitudinal or transverse section. The images are z-projections. White bars: 50 µm; yellow bars: 100 µm.

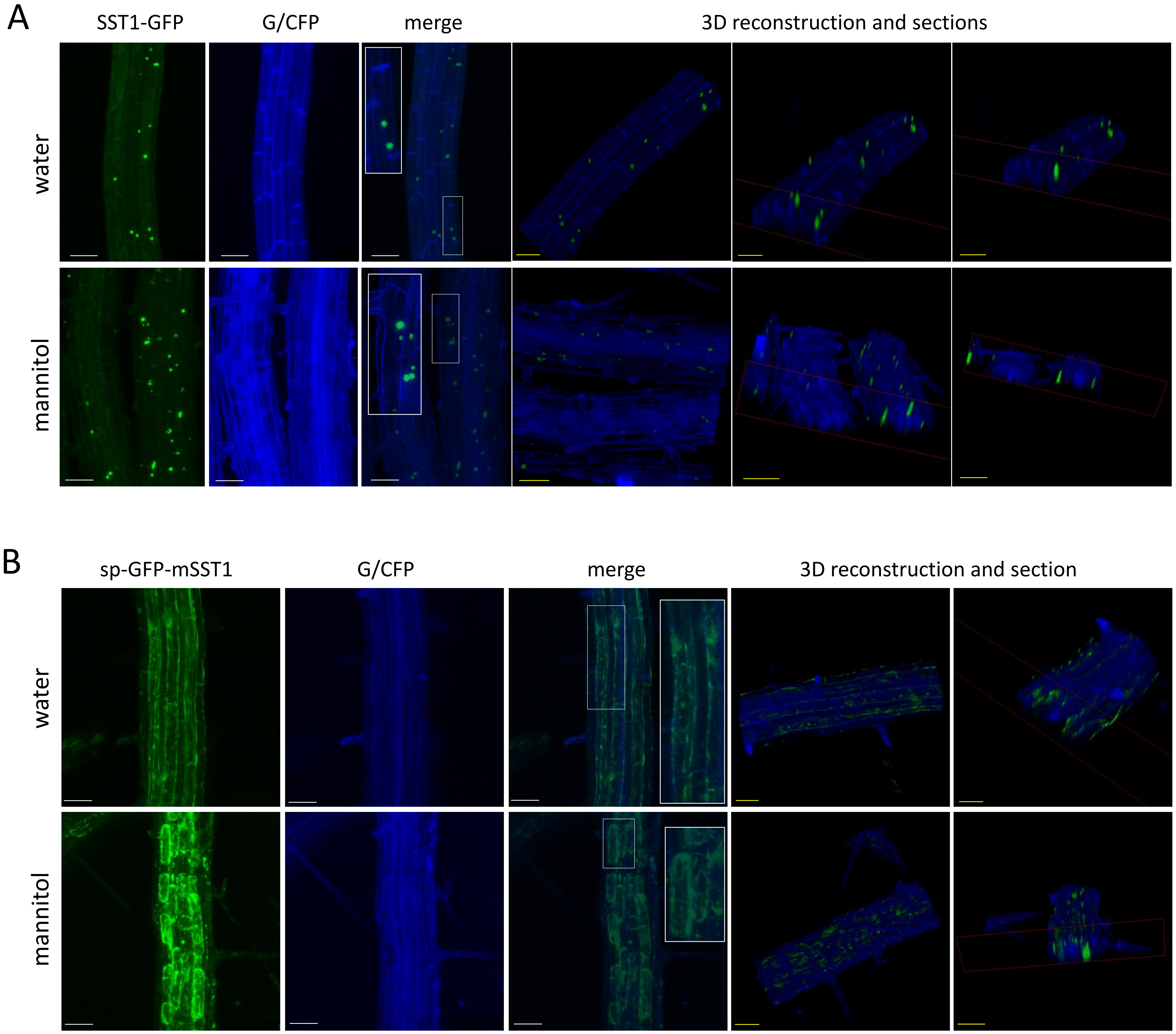

Supplement: Supplementary file 1 [file plants-14-01117-s001.zip › Figure S8.pdf]
