# Supplementary material for: A Comprehensive Analysis of Short Specific Tissue (SST) Proteins, a New Group of Proteins from PF10950 That May Give Rise to Cyclopeptide Alkaloids
Source: Plants (Basel). 2025 Apr 3;14(7):1117. doi: 10.3390/plants14071117 (PMC11991032; doi:10.3390/plants14071117)
Supplement: Supplementary file 1 [file plants-14-01117-s001.zip › List S1.pdf]

**List S1**. List of species analyzed in this research. The species have been grouped by the existence of SST genes on them and the source of the sequence. The species are included in taxonomic groups and sorted alphabetically. Parentheses, when present, indicate the version of genomic data in the database.

**Species with *SST* genes from Phytozome database (phytozome v13, June 2022)**

- **Tracheophyte:**
  - **Core-eudicots:** *Alyssum linifolium (v1.1), Amaranthus hypochondriacus (v2.1), Anacardium occidentale (v0.9), Arabidopsis halleri (v1.1), Arabidopsis lyrata (v2.1), Arabidopsis thaliana (TAIR10), Arachis hypogaea (v1.0), Beta vulgaris (EL10_1.0), Betula platyphylla (v1.1), Boechera stricta (v1.2), Brassica oleracea capitata (v1.0), Brassica rapa (FPsc v1.3), Cakile maritima (v1.1), Capsella grandiflora (v1.1), Capsella rubella (v1.1), Caulanthus amplexicaulis (v1.1), Carya illinoinensis (v1.1), Chenopodium quinoa (v1.0), Cleome violacea (v2.1), Carica papaya (ASGPBV0.4), Castanea dentata (v1.1), Cicer arietinum (v1.0), Citrus clementina (v1.0), Citrus sinensis (v1.1), Coffea arabica (v0.5), Corymbia citriodora (v2.1), Crambe hispanica (v1.1), Cucumis sativus (v1.0), Daucus carota (v2.0), Descurainia sophioides (v1.1), Diptychocarpus strictus (v2.1), Eruca vesicaria (v1.1), Eucalyptus grandis (v2.0), Euclidium syriacum (v1.1), Eutrema salsugineum (v1.0), Fragaria vesca (v4.0.a2), Glycine max (Wm82.a2.v1), Glycine soja (v1.1), Gossypium barbadense (v1.1), Gossypium darwinii (v1.1), Gossypium hirsutum (v2.1), Gossypium mustelinum (v1.1), Gossypium raimondii (v2.1), Gossypium tomentosum (v1.1), Helianthus annuus (r1.2), Hydrangea quercifolia (v1.1), Iberis amara (v1.1), Isatis tinctoria (v1.1), Kalanchoe fedtschenkoi (v1.1), Kalanchoe laxiflora (v1.1), Lactuca sativa (V8), Lepidium sativum (v1.1), Lunaria annua (v1.1), Lindenbergia philippensis (v1.1), Linum usitatissimum (v1.0), Lotus japonicus (Lj1.0v1), Lupinus albus (v1), Malcolmia maritima (v1.1), Malus domestica (v1.1), Manihot esculenta (v8.1), Medicago truncatula (Mt4.0v1), Mimulus guttatus (TOLv5.0), Myagrum perfoliatum (v2.1), Phaseolus acutifolius (WLD v.2), Phaseolus lunatus (V1), Phaseolus vulgaris (v2.1), Olea europaea (v1.0), Poncirus trifoliata (v1.3.1), Populus deltoides (WV94 v2.1), Populus trichocarpa (v4.1), Portulaca amilis (v1.0), Prunus persica (v2.1), Quercus rubra (v2.1), Ricinus comunis (v0.1), Rorippa islandica v1.1, Salix purpurea (v5.1), Schrenkiella parvula (v2.2), Sinapis alba (v3.1), Solanum lycopersicum (ITAG2.4), Solanum tuberosum (v6.1), Spinacia oleracea (Spov3), Stanleya pinnata (v1.1), Thlaspi arvense (v1.1), Theobroma cacao (v2.1), Trifolium pratense (v2), Vaccinium darrowii (v1.2), Vigna unguiculata (v2.1), Vitis vinifera (v2.1).*

**Species with *SST* genes from sources other than phytozome**

- **Tracheophyte:**
  - **core-eudicots:** *Arachis ipaensis, Cajanus cajan, Camelina sativa, Capsicum annuum, Coffea canephora, Corchorus olitorius, Cucurbita maxima, Cynara cardunculus, Dorcoceras hygrometricum, Gossypium arboreum, Handroanthus impetiginosus, Helianthus annuus, Herrania umbratica, Ipomoea nil, Jatropa curcas, Juglans regia, Lupinus angustifolius, Morus notabilis, Nicotiana attenuata, Nicotiana sylvestris,*

*Nicotiana tabacum, Parasponia andersonii, Phaseolus coccineus, Populus euphratica, Prunus avium, Punica granatum, Quercus suber, Raphanus sativus, Sesamum indicum, Solanum pennelli, Trema orientalis, Vigna angularis, Ziziphus jujuba*

**Species without *SST* genes from Phytozome database (phytozome v13, June 2022)**

- **Rhodophyte**: *Porphyra umbilicalis*
- **Chlorophyte**: *Botryococcus braunii*, *Chlamydomonas reinhardtii*, *Chromochloris zofingiensis, Coccomyxa subellipsoidea*, *Dunaliella salina*, *Micromonas pusilla*, *Micromonas sp*, *Ostreococcus lucimarinus*, *Volvox carteri*
- **Embryophyte**: *Ceratodon purpureus, Marchantia polymorpha, Physcomitrium patens, Sphagnum fallax, Sphagnum magellanicum*
- **Tracheophyte:**
  - **Fern:** *Ceratopteris richardii*
  - **Moss**: S*elaginella moellendorffii*
  - **Conifer:** *Thuja plicata*
  - **Basal angiosperm:** *Amborella trichopoda (v1.0) (Amborellaceae), Nymphaea colorata (v1.2) (Nymphaeaceae )*
  - **Basal eudicot:** *Aquilegia coerulea (v3.1) (Ranunculaceae*
  - **Monocots**: *Acorus americanus, Ananas comosus, Asparagus officinalis, Brachypodium distachyon, Brachypodium hybridum, Brachypodium mexicanum, Brachypodium stacei, Brachypodium sylvaticum, Chasmanthium laxum, Dioscorea alata, Eleusine coracana, Hordeum vulgare, Joinvillea ascendens, Miscanthus sinensis, Musa acuminata, Oropetium thomaeum, Oryza sativa, Panicum virgatum, Panicum hallii Paspalum vaginatum, Pharus latifolius, Setaria italica, Setaria viridis, Sorghum bicolor, Spirodela polyrhiza, Thinopyrum intermedium, Triticum aestivum, Urochloa fusca, Zea mays, Zostera marina*
  - **Ancient angiosperm:** *Cinnamomum kanehirae (v3) (Lauraceae)*
