# Supplementary material for: A Comprehensive Analysis of Short Specific Tissue (SST) Proteins, a New Group of Proteins from PF10950 That May Give Rise to Cyclopeptide Alkaloids
Source: Plants (Basel). 2025 Apr 3;14(7):1117. doi: 10.3390/plants14071117 (PMC11991032; doi:10.3390/plants14071117)
Supplement: Supplementary file 1 [file plants-14-01117-s001.zip › List S2.pdf]

List S2. List of the 194 SST protein sequences in FASTA format. The sequence numbers are the same as in Table S1.

```
>1.Alyssum linifolium SST1
MQRQRYLFVFLTVLFSFLLLVNLSEGRSGGVAEEYWKKMMKNEPLPEPIKELLNNPFRNT
QEIFIQNFDPKSVAIIYRSPKE
>2.Alyssum linifolium SST2
MKKQGDLVVFLIVLFSFLLLVNPSEGRPARGVAEEYWRKMMKNEPFPEPIKELLNNPFRT
AQERFIPNFDTKVLVLIYHNPHHD
>3.Alyssum linifolium SST3
MKKQGDLIVFLIVLFSFLLLVNPSEGRPARGVAEEYWRKMMKNEPFPEPIKELLNNPFRT
AQERFIPNFDTKVLVLIYHNPHHD
>4.Alyssum linifolium SST4
MQRQRYLFVLLTVLFSFLLLVNRSEGRSEGGVAEEYWKKMMKNEPLPEPIKELLNNPFRN
TQEIFIQNFDPKSVAIIYRSPKE
>5.Arabidopsis halleri SST1
MKQQRYLVVFIVLFSFLLFVNLSEGRTGGVAEEYWKKMMKNEPLPEPIKELLNNPFRTGQ
ERFIQNFDTKSVVIIYHNPNE
>6.Arabidopsis lyrata SST1
MKQQHYLVVFIVLFSFLLFVNLSEGRTGGVAEEYWKKMMKNEPLPEPIKELLNNPFRTGQ
ERFIQNFDTKSVVIIYHNPNE
>7.Arabidopsis lyrata SST2
MKQQRYLVVFIVLFSFLLFVNLSEGRTGGVAEEYWKKMMKNEPLPEPIKELLNNPFRTGQ
ERFIQNFDTKSVVLIYHNPNE
>8.Arabidopsis thaliana SST1
MKQQQRYLVVFIVLLSFLLFVNLSEGRTGGVAEEYWKKMMKNEPLPEPIKELLNNPFRTA
QERFIQNFDTKSVVIIYHNPNE
>9.Boechera stricta SST1
MNQQRYSVVFLIVLFIFLLFVNLSEGESGRGVAEEYWKKMMKNEPLPEPIKELLSNPFRT
AQEMFIQDFDTKSVVLLYRNPNK
>10.Boechera stricta SST2
MKQQRYLVVVFFILFSFLLFVNLSEGRSGGGGVAEEYWKKMMKNEPLPEPIKELLNNPFR
TAQERFIQSFDTKSVVIIYHRPNE
>11.Brassica oleracea SST1
MKKQRYLVFFFVLFSFLLFVNLSEARSGGVAEEYWKKIMKNEPLPEPIKELLNNPFRTGE
ERFVKDFKTKSIVIIYHNPNV
>12.Brassica oleracea SST2
MKQQHFLVAFFVVLFSFLLFVYLSEGKSEVAEDYWKKMMKSEPLPEPIKDILNNPFRTGQ
ERFAKNFNTKSVVIIYHNPNV
>13.Brassica rapa SST1
MKQQHYLVVVFFVLFSFLLFVNLSEGRSGGVAEEYWKKMMKDEPLPEPIKDLLNNPFRTG
QERFVKDFNTKSIVIIYHNPNV
>14.Brassica rapa SST2
MKKQRYLVFVFVLFSFLLFVNLSEGRSGGVAEEYWKKIMKNEPLPEPIKELLNNPFRTGE
ERFVKDFKTKSIVIIYHNPNV
>15.Brassica rapa SST3
MKQQRFLVAFFVVLFSFLLFVYLSEGKSEVAEDYWKKMMKSEPLPEPIKDILNNPFRTGQ
ERFAKNFNTKSVVIIYHNPNV
>16.Cakile maritima SST1
MNQQRYLAVFFFVLFSFLLFVNLGEGRSGGVAEEYWKKIMKNEPLPEPIKELLNNPFRTG
EDRFVKDFKTKSIVIIYHNPNV
>17.Cakile maritima SST2
MRQQRYLVVFFFVLFSFLLFVNLSEGRSGGVAEEYWKKMMKNEPLPEPIKELLKNPFRTG
EERFVKDFNTKLVVIIYHNPNV
>18.Cakile maritima SST3
MKQQHFLVAFFVVLFIFLMFVNLSEGKSGVAEEYWKKMMKNKPLPEPIKDFLNNPFRTGP
ERFVKNFNTKSVVIIYHNPNV
>19.Camelina sativa SST1
MKQQRYLVVVFFILFSFLLFVNLSEGRSGGGGVAEEYWKKMMKNEPLPEPIKELLYNPFR
TAEERFIQSFDTKSVVIIYHNPKE
```

>20.Capsella grandiflora SST1
MKQQRYLVVLFFILFSFLLFVNLSEGRSGGGVAEQYWKKMMKNEPLPEPIKELLYNPFRT
VDERFIQSFDTKSVVIIYHNPNE
>21.Capsella grandiflora SST2
MKQQHYLVVFLIVLFTFLLFVNTRSGRSGGRDGVAEEYWKKMMKNEPLPEPIKELLKNPF
RTAQEMFIQDFNTKSVVLIYHNPHE
>22.Capsella rubella SST1
MKQQRYLVVLFFILFSFLLFVNLSEGRSGGGVAEQYWKKMMKNEPLPEPIKELLYNPFRT
VDERFIQSFDTKSVVIIYHNPNE
>23.Capsella rubella SST2
MKQQHYLVVFLIVLFTFLLFVNTRSGRSGGRDGVAEEYWKKMMKNEPLPEPIKELLKNPF
RTAQEMFIQDFNTKSVVLIYHNPHE
>24.Caulanthus amplexicaulis SST1
MKQQRYLVVFFFVLFSFLLFVNLSEGRSGGVAEEYWKKMMKNEPLPEPIKELLNNPFRTG
QERFVKNFNTKSIVIIYHNPNV
>25.Caulanthus amplexicaulis SST2
MKQQRYLVVFFFVLFSFLLFVNLSEGRSGGVAEEYWKKVMKNEPLPEPIKELLNNPFRIG
QERFVKDFNTKSIVIIYHNPNV
>26.Cleome violacea SST1
MKRLLVVFFIFSFFLLVNLNEGRREAEDYWKKTMKDEPLPEPIKDLLNNPFRTDQKFIHN
FDTKSLALIYHKN
>27.Cleome violacea SST2
MKRFSFSVFFVLLSIFLLFGVLTQARMEPEEYWKKAMAEAELPEPIKELMNNPFRSKHEK
FINNFGTKATVLIYHNPNP
>28.Crambe hispanica SST1
MKQQRYLVVLFFVLFSFLLFVNLSEGRSGGVAEEYWKKIMKNEPLPEPIKELLNNPFRTG
EERFVRDFKTKSIVIIYHNPNV
>29.Crambe hispanica SST2
MKQQHFLVAFFVVLFSLLLFVNLSEGKSGVAEEYWKKMMKNEPLPEPIKDFLNNPFRAGQ
ERFAKNFNTKSIVIIYHNPNI
>30.Descurainia sophioides SST1
MQRQRYLFVFLTVLFSFLLLVNLSEGRSEGGVAEEYWKKMMKNEPLPEPIKELLNNPFRN
TQEIFIQNFDPKSVAIIYRSPKE
>31.Descurainia sophioides SST2
MKKQGDLVVFLIVLFSFLLLVNPSEGRPARGVAEEYWRKMMKNEPFPEPIKELLNNPFRT
AQERFIPNFDTKVLVLIYHNPHHD
>32.Diptychocarpus strictus SST1
MKQQRYVVVVFILFSFLLFVNMIEGRSGGGGVAEKYWKKVMKNEPLPEPIKELLNNPFRT
AKERFIKNFNTKSVVIIYHNPNE
>33.Euclidium syriacum SST1
MKQQRYVVVVFFILFSFLLFVNLIEGRPGEGVAEKYWKKVMKNEPLPEPIKELLNNPFRT
AQERFIKNFKTKSIVIIYHNPNE
>34.Eutrema salsugineum SST1
MKQQRYLVVFFFVLFSFLLFVNLSEGRSGGVAEEYWKKVMKNEPLPEPIKELLNNPFRTA
KERFVKNFNTKSIVIIYHNPNV
>35.Eruca vesicaria SST1
MKQQFCLVAFFVFSFLLFVNLSEGKSGVPEDYWKKMMKKEPLPEPITDILNNPFRKGQER
FVKNFNTKSIVIIYHNPNA
>36.Eruca vesicaria SST2
MKQQRYLVLFFFVLFSFLLFANLSEGRSGGVAEEYWKKIMKNEPLPEPIKELLNNPFRTG
EDRFVKDFKTKSIVIIYHNPNV
>37.Iberis amara SST1
MMNQQRYLVVFFFVLFIFLMFVNPSEGRSGGGVAEEYWKKMMKNDPLPEPIKELLNNPFR
TEKERFITNFNTKSILIIYHDPLV
>38.Iberis amara SST2
MKQQHYIVVVFFVLFSFLLFVNLREGRSGREVIEEYWKKMMKNEPLPEPIKQILNNPFRT
AQEKFVTNFDTHSVLIIYHNPIE
>39.Isatis tinctoria SST1
MKQQRYLVVFFFVLFSFLLFVNLSEGRSGGVAEEYWKKMMKNEPLPEPIKELLNNPFRTG
DERFVRDFNTKSIVIIYHNPNV
>40.Lepidium sativum SST1

MKQQRYLVVFFVVVFSFLMFVNLSEGRSGREVIEEYWKKTMKNEPLPEPIKVLLNNPFRT
GDERFIQSFDTKSVVIIYHSPKE
>41.Lunaria annua SST1
MKQQQRYLFVVFFVLFSFLLIVNLSEGRSGGGGVAEEYWKKMMKNEPLPEPIKELLHNPF
RTEQEMFITNFDTKSVVIIYHNPNE
>42.Malcolmia maritima SST1
MMKQQGYLVVFLIVLFSFLLFVDLSEGRPRGGVQEEYWKKMMKNEPLPEPIKELLYNPFR
TGQGLFTKDFDTKSAVLIYHNPNE
>43.Malcolmia maritima SST2
MKQQRYLVVVFFVLFSFLLFVNMSEGRSGGVAEKYWKKIMKNEPLPEPIKELLYNPFRTG
DERFIQSFDTKSVVIIYHTPNE
>44.Myagrum perfoliatum SST1
MKQQRYLVVFFFVLFSFLLFVNLSEGRSGGVAEEYWKKMMKNEPLPEPIKELLNNPFRTG
EERFVKDFNTKSIVIIYHNPNV
>45.Raphanus sativus SST1
MKKQRYLVFFFVIFSFLLFVNLSEGRSGGVAEEYWKKIMKNEPLPEPIKELLNNPFRTGE
ERFVKDFKTKSIVIIYHNPNV
>46.Rorippa islandica SST1
MKQQHYLVVVFFVLFTFLLFVNLSEGRPGGVAEEYWKKMMKNEPLPEPIKELLNNPFRTA
QEKFIQNFDTKSVVIIYHNPKE
>47.Schrenkiella parvula SST1
MKQQRYLVVFFFVLFSFLLFVNLSEERSGGVAEEYWKKMMKNEPLPEIIKELLNNPFRTG
RERFVKNFNTKSIVIIYHKPNV
>48.Sinapis alba SST1
MRKQRYLVFFFFVLFSFLLFVNLSEGRSGGVAEEYWKKIMKNEPLPEPIKELLNNPFRTG
EERFIKDFKTKSIVIIYHNPNV
>49.Sinapis alba SST2
MKQQRYLVVFFFVLFSLLLFVNQSEGRLGGVAEEYWKKMMKDEPLPEPIKDLLNNPFRTG
QERFVKDFKTKSIVIIYHNPNV
>50.Sinapis alba SST3
MKQQHFLVAFFVVLFCFLLFVNLSEGKSGVAEEYWKKMMKSEPLPEPIQDLLNNPFRTGQ
ERFVKNFNTKSIVIIYHNPNV
>51.Sinapis alba SST4
MKQQHFLVAFFVVLFCFLLFVNLSEGKSGVAEEYWKKMMKSEPLPEPIQDLLNNPFRTGQ
ERFVKNFNTKSIVIIYHNPNV
>52.Stanleya pinnata SST1
MKQQRYLVVFFFVLFSFLLFVNLSEGRSGGVAEEYWKKMMKNEPLPEPIKDLLNNPFRTG
QERFVKNFNTKSIVIIYHNPNV
>53.Stanleya pinnata SST2
MKQQRYLVVFFFVLFSFLLFVNLSEGRSGGVAEEYWKKVMKNEPLPEPIKELLNNPFRIG
QERFVKDFNTKSIVIIYHNPNV
>54.Thlaspi arvense SST1
MKQQRYLVVFFFVLFSFLLFVNLSEGRSGGGVAEEYWKKMMKNEPLPEPIKELLDNPFRT
ARERFVKNFNTKSIVIIYHNPNV
>55.Arachis hipogea SST1
MKSSTFALFLVFSFLLVANNVCHGRKDQGEYWKDMMDKQTMPEAIKDLLVEDPQVSSSHA
RKNKDQFRKDFDIKPNVILYHSHVGPKKQKA
>56.Arachis ipaensis SST1
MKPFFAFFIVFSLLLGANLSYGRKELVGEYWKKMIKGQSMPEAIKEILVVEDPQISSSDS
SRTKDNFIRDFDIKPNVILYHSHKQKQKHNHKNPFLNNNLEEPEFQETENK
>57.Cajanus cajan SST1
MKSNFAVIVVFSLLLVANFGCARKDLGAYWKNMMKGQAMPQAIKDLVEASDAVDAGTKNR
FVRDFDVKPNVILYHTHVKSSKQKQKLFLNNNQD
>58.Cicer arietinum SST1
MKSIFALCIVFSFLLVADLSYARKDLVDYWKNMMNDQPMPETIKELVQNPQVTDAGNDKF
IRNFDIRPNVILYHTHVDSNKKQHVFVNNSEQDHFHGITRKHG
>59.Cicer arietinum SST2
MVGNLSYGRKDVGDYWMNMMNEQAMPEAIKNLVQVPIVSNKRKEDRFITDFDVHPNIILY
HTHVHQKQP
>60.Cicer arietinum SST3
MVGNLSYARKDVGEYWKNKMNEQAMPEAIKNLVQVQKQDHFVRDFDVHPNIILYHTHVHE

KQEKPLAP
>61.Glycine max SST1
MKSNFAVFVVFSLLLIANLSCARKDLGWYWKNVMKEQPMPQAIKDLVEDSQASAAGKKDR
FIRDFDVKPNVILYHTHVVPMKQKHKHKQNPFVKNQD
>62.Glycine max SST2
MKSNFAVFVVFSLLLVANLSCARKDLGGYWKNMMKEQPMPQAIKDLVEDSQASDTGKKDL
FTRDFDVKPNVILYHTHVVSMKQKQKPFLQN
>63.Glycine max SST3
MILAFTNLMRTPYLSKKMKSFFAFFVVFSLLVVNLSYARKDMGDYWKNMMNGQPMPEAIK
DLLVQDPQVSDAMKDHFIRDFDIKPNVILYHTHVVPHKHKQKIQQAMAKKLEPEFQGTER
HG
>64.Glycine max SST4
MCVDHFLPIKRDAQRLKVLAFTNLMRTPYLSKKMKSFAFFVVFSLLVVNLSYARKDMGDY
WKNMMNGQPMPEAIKDLLVQDPQVSDAVKDHFIRDFDIKPNTILYHTHVVPNKQKQKQNI
QQAMAKKLEPKFQGTERHG
>65.Glycine max SST5
MKPISALFVILSLLLVANINLSHARKDLGDYWKKMMNDQPMPEAIKENCSSSTSIRWKNG
SFYKGLQYKA
>66.Glycine max SST6
MKPIFALFVVLSLLLVANINLSHARKDLGDYWKKMMNDEPMPEAIKDLIQDQQVQDATAD
HFIRYFDMKPNIILYHTHVVSKKQQQQKAFDHKFKPVSRNGKSWLNKP
>67.Glycine max SST7
MNPISALFVILSLLLVSNINLSHARKDLGDYWKKMMNDQPMPEAIKENCSSSTSIRCKNG
SLYKGLQYKA
>68.Glycine soja SST1
MKSNFAVFVVFSLLLVANLSCARKDLGGYWKNMMKEQPMPQAIKDLVEDSQASDTGKKDL
FTRDFDVKPNVILYHTHVVSMKQKQKPFLQN
>69.Glycine soja SST2
MKSNFAVFVVFSLLLIANLSCARKDLGWYWKNVMKEQPMPQAIKDLVEDSQASAAGKKDR
FIRDFDVKPNVILYHTHVVPMKQKHKHKQNPFVKNQD
>70.Glycine soja SST3
MILAFTNLMRTPYLSKKMKSFFAFFVVFSLLVVNLSYARKDMGDYWKNMMNGQPMPEAIK
DLLVQDPQVSDAMKDHFIRDFDIKPNVILYHTHVVPHKHKQKIQQAMAKKLEPEFQGTER
HG
>71.Glycine soja SST4
MKPVFALFVVLSLLLVANINLSHARKDLGDYWKKMMNDEPMPEAIKDLIQDQQVQDATAD
HFIRYFDMKPNIILYHTHVVSKKQQQQKAFDHKFKPVSRNGKSWLNKP
>72.Glycine soja SST5
MKPISALFVILSLLLVANINLSHARKDLGDYWKKMMNDQPMPEAIKENCSSSTSIRCNNG
SLYKGLQYKA
>73.Glycine soja SST6
MKPISALFVILSLLLVANINLSHARKDLGDYWKKMMNDQPMPEAIKENCSSSTSIRCKNG
SFYKGLQYKA
>74.Glycine soja SST7
MCVDHFLPIKRDAQRLKVLAFTNLMRTPYLSKKMKSFAFFVVFSLLVVNLSYARKDMGDY
WKNMMNGQPMPEAIKDLLVQDPQVSDAVKDHFIRDFDIKPNTILYHTHVVPNKQKQKQNI
QQAMAKKLEPKFQGTERHG
>75.Lotus japonicus SST1
MKSIFAVFLVISLLMATNTNFSYARKDLGDYWKDMMNGQPMPEAIKDLLVEDPQVSDAAG
KDHFTRDFDIRPNVILYHTHVESKKQNQKQKKQLFVKNFQLPEFEGITGSHG
>76.Lotus japonicus SST2
MKSISAMFVVFSVLLIVNLSHGRKDMGGYWKNVMNGQPMPEVVKDLIQDPHASDAGKDHF
IRDFDIKPNNVSIFHTHVVPTSRSRRHLTRNLN
>77.Lotus japonicus SST3
MKSIFAVFAVFSILLIANCSCATRDLGDYWKNMMKGQAMPEAIKELVQDPQASYAGKDRF
IRDFDIRPNVILYHTHVGSSTKQKQNTFAKN
>78.Lotus japonicus SST4
MSHLTWSPHLSKEMKSISAMLVVFSILLVVNLSYARKDMGEYWKNVMNDQPMPEVVKDLI
EDPQVSDAGKDHFIRDFDIKPNNATIFHTHVVPTSRSRRHLTRNLN
>79.Lupinus albus SST1
MSSIFVMSLVFSLLLVANLSCARKDVEDYWKNKMKGQPMPEPIKDLTYDLVAASDAGKSR

FIRDFDIKPNVILYHTHVVSKEQKQKNPFVKKIETKFKKPEVMVVEQTVKKD
>80.Lupinus albus SST2
MKSIFALFLVFSLLLVANLSYAIKHEGEYWKNIMKDQPMPETIKDLLVQDPQVLDSGKDH
FIRDFDIRPNVILYHTHVVSKKQKQHPFVNNFEPEFQE
>81.Lupinus angustifolia SST1
MMKSIFALFLVFSFLLVTNLSYARQHQGEYWKNIMKDQPMPETIKDLLVQDPQAYTEKYH
FIRDFDIRPNVILYHTHVVSKKQKQHPFVKNFEPEFQEIGTRV
>82.Medicago truncatula SST1
MKSIQALFLVFSLLLVANMSYARKDLGDLWKNKMNKQPMPEAIKNFIQVPKALGEGKEDH
SFTTDFDVNPNIILYHTHVHQDEKPFEHAARKMESLLPKRG
>83.Medicago truncatula SST2
MKSILVVFLLFSLLLVTNLSCATKENMVEYWKNMMKGEAMPEAIKGLNSGSTSNICREWT
LM
>84.Medicago truncatula SST3
MKSIFASFILFSLLLVADLSYARKDLGGYWKNMMNDQPMPEAIKELVQNQEVSDDFIRDF
DVKPNVILYHTHVESKKKKEHVFVKNSQQELHGT
>85.Medicago truncatula SST4
MKSIQALFLVFSLLLVANLSYARKDLGDYWKNKMNEQPMPEAIKNLIQVPKALDEGKEDH
SFTTDFDVNPNIILYHTHVHQDEKPFQHAARKMEPLLPKRG
>86.Phaseolus acutifolius SST1
MKSNFAIFVVFSLLLVGSFSCARKDMRGYWKDMMKEQPMPQAIKDLIEDSEVSEAGKGRF
VRDFDVKPNVILYHTHVVPMKQRQKNQD
>87.Phaseolus acutifolius SST2
MRTPHVSKKMKSYFAFFLIFSLLLVVNLSYARKDMGDYWKNMMNGQPMPEAIKDLLVQDP
QVSDTVKDHFIRDFDIRPNVILYHTHIPPNKRKQHAMAKKIEEFHGTGN
>88.Phaseolus coccineus SST1
MKSNFAIFVVFSLLLLVGSCSCARKDMRGYWKDMMKEQPMPEAIKDLIEDSEEVSEAGKG
RFVRDFDVKPNVILYHTHVVPMKQRQKNKD
>89.Phaseolus lunatus SST1
MKSNFAVFVVFSLLLVGSFSCARKDMRGYWKDMMKEQPMPQAIKDLIEDSEASEAGKGRF
VRDFDVKPNVILYHTHVVPRKQRQKKQD
>90.Phaseolus lunatus SST2
MRTPYVSKKMKSYFAFFLIFSLLLVVNLSYARKDMGDYWKNMMNGQPMPEAIKDLLVQDP
QVSDTVKDHFIRDFDIRPNVILYHTHIPPNKRKQHAMAKKIKEFHGTGRHA
>91.Phaseolus vulgaris SST1
MKSNFAIFVVFSLLLLVGSCSCARKDMRGYWKDMMKEQAMPEAIKDLIEDSEEVSEAGKG
RFVRDFDVKPNVILYHTHVVPMKQRQKNKD
>92.Phaseolus vulgaris SST2
MRTSHASKKMKSYFAFFLIFSLLLVVNLSYARKDMGDYWKNMMNGQPMPEAIKDLLVQDP
QVSDTVKDHFIRDFDIRPNVILYHTHIPPNKRKQHAMAKKIEEFHG
>93.Trifolium pratense SST1
MKSPNFTKEMKSIFALFILFSLLVADVSYARKDLDDFWKNMMNDQPMPEAIKELVQNTKV
IDSRKDNFIRDFDVKPNLILYHTHVESKKQKRKQHIFVKKSEQEEFHGTQKHG
>94.Trifolium pratense SST2
MKSTLVVFLLFSLLLVANLSFARKDPGEYWKNMMKGEAMPEAIKELIQDPQAIYAGKDGF
MRDFDVKPNAILYHTHVMSMEQTRRC
>95.Trifolium pratense SST3
MSLVSFKNKYTKESKLLRNPQIMKSISIQVLFLVFSLLMVANMSHGRKDLGDYWKNKMNE
QPMPEAIKNLIQVPKALSSNEEKDNSFNRDFDVHPNVILYHTHVHEEKKPFDESAVRKME
SLLPNKG
>96.Vigna angularis SST1
MRTSYLSKKMKSNFAFLVIFSLLLVVNLSYARKYMGDYWKNMMNGQPMPEAIKDLLVQDP
QESDAAAVKDHFIRDFDIRPNIILYHTHDMSRKQKQHAMAKKIEELQEPGRHG
>97.Vigna unguiculata SST1
MKSNVAVFVVFSLLLVGSLSSARKDLDGYWKETMKEQPMPEAIKDLIEDSQVSETGKDRF
IRDFDVKPNVILYHTHVVSMKQTQKNQD
>98.Amaranthus hypocondriacus SST1
MKFPPHYFFLFFIILLMFNNEISARKGPEEYWKEIMKDQALPEAINDIIDPKRVNPSEKN
IILDSF
>99.Amaranthus hypocondriacus SST2
MEGYHNLKIMLVVLLFVFLLGNPIDGRREPGEYENDMMMKEKVIPEAIKDFILYDKLYDA

HKLAQAIKDISEDFDSTPNATIYHDDADQE
>100.Anacardium occidentale SST1
MKSTFAFVFFFSLLLFGCSLSYARKEPGDYWKSLMKEQPMPKAIKDLLHQDSEGRKIDHF
AKDFDVNPNVIIYHSHSEPKQERQEEKSLVNQIKSQRG
>101.Daucus carota SST1
MHSKLALLVSLALLFIASTSYARPSPRQYWHVTKGEAMPMFQDFDTMNYDAGLLYHGSAL
AEDKFVQDSVHEKLDFGDDRNDSYLKRKSSDESG
>102.Cynara cardunculus SST1
MKSLSTFVFLFSLVLLARMNDARKGPEEYWRSIMKDEPMPKAIQDVLSEDSTDKENNRDR
FTRDFDTKPNLIIYHSHVMYNQKDHELASSKIN
>103.Helianthus annuus SST3
MRSLFAILFIFSLSLLTLASVNDARMGPEEYWRSVMKDEPMPETLQNVLVHDSSSLEDKE
KKKDRFIRNFDTKPNLIIYHSHVMYNQKGHELASSKLN
>104.Helianthus annuus SST2
MMKLKSLSTLITFFSLLLILSRIEARSDPREYWRSVMKDEPMPKTIQDVLPLEDGMKVNK
DIFTRNFNLKPNLIIYRSHVVYSEKNHHIVSSSSSSFDELN
>105.Helianthus annuus SST3
MRSLTVFLFLFLVLLNSSLYDARKDPEEIWRSVMKDEPMPKTIQDALSQDSTRSNKEVNM
KDQSVSVFDTQPNHKMFQRDFDTKPNSMTFLYPKPTL
>106.Helianthus annuus SST4
MRSLSVFLVFFSLILLASIHEARKDPKDWRNAMKYEPIPPDALSQDSTTLNDKENNKDQF
VRDFDKQPNLKMFAKGFNPKPSSLVSNGCPPKQD
>107.Lactuca sativa SST1
MRSLSTGLFFFSLILLASLHDAREGPKEYWRSVMKDEPMPKAIQDVLIQDSARSNNNKDR
FTRNFDTKPNLIIYHSHVIYNQKDHELASSKMN
>108.Lactuca sativa SST2
MFCAGSIADDLGLPFIIVRSSSSASSSDSDSESDDEAQAALELQTLETKLSTRSSEAAAQ
LASLHDAREGPKEYWRSVMKDEPMPKAIQDVLIQDSARSNNNKDRFTRNFDTKPNLIIYH
SHVIYNQKDHELASSKMN
>109.Betula platyphylla SST1
MSCYKVVIYHLARRNLGHTEREEKKKKRPWFCLLNIKMRTVFAFLTLLSLLLVLFAGRDV
VARKDLGGYWKNVMDSQPMPEAIQGRIYPQTGKGVHFAKDFDPVSLVDSNIVWVPPPGDH
LIHPEARKEDHLPRSSSRPKAENSPISKAKP
>110.Handroanthus impetiginosus SST1
MRAFSTFFLLSLMLFVYVCDARKDPGDYWKSIMNGEPMPKAITDLIHRYNLESDSNMKMD
YFIKNFNTKANVIIYHSHHKVHSDHKPKVSNMKLV
>111.Trema orientalis SST1
MKSISAFVVLCSILLLGSLSYARKDAGDYWKSIMKNQPMPDAIKDLFHDEGLPSLPPGSA
KRDRFVKDFDVRPNVIIYHSSHSQPEGVLNDHMPSVHEFQTKHHQQLQQVINKISRD
>112.Carica papaya SST1
MTLSFFLLLLLLLLLIGSSSVDGRKEAGGEYWGSVMKGQAMPEVVEALTYPNSPTTTQPQ
QTQPKRESRNFEDAWR
>113.Beta vulgaris SST
MKFPCVFFIFFFILLMFSNEISARKNPEEYWKKVMKDQPIPEVINGIIDEEIMAKSYEKET
FWKHFKRDFDVNSNVIIYHSHQENNHLSPST
>114.Chenopodium quinoa SST1
MKFACVLFLSFFMLLLFSNEIGARNSPEDYWKKVMKDQPIPEAINGIIDQKMANASEKNS
LWSHFKRDFDVTSNVIIYHPHQVNNHRSPSD
>115.Spinacea oleracea SST1
MKFPCVIFISFFTLLMFANEIGARNSPEDYWKKIMKDQPIPEAIGGIIDQEMANPSEKKP
FWSHFKRDFDVTSNVIIYHPHQENTMRSPSA
>116.Ipomoea nil SST1
MKTLSPFILFAFLVSTGFFAAEARRDPGEYWDAMMNGDPMPKAITDLLLINQDPSSSSSS
PNDRFIRDFDTKPNLIIYHSHVDVYPKKHEVVAKDVQQKKT
>117.Kalanchoe fedtschenkoi SST1
MAAAAQFQLPSLNAMHWRLFLAIFTLISVCEAAGGRKELSSYWRNVMKDEDMPEAIKGLV
VAGDGQELQREFRPRPNLIIYHNGPDHRQKHWRLEDVKGLQQQDSATEPGV
>118.Kalanchoe fedtschenkoi SST2
MHDQTTHHITMKQPCFSLLSISIILLSLLVALAAWNANARKDSGDYWKGVMKDEPMPAAL
EALILPIPNTSSSQSSLVKKPDCRHEDPTASHVKGKTFGYDIEPRPSVTVRPQVQQRIK
>119.Kalanchoe fedtschenkoi SST3

MHRRLLFALFTLFCASELYLKQVCEAVDGRRELSSYWRDVMKDEDMPEAIQGLVLGDGQK
MQETSFDREFQARPNLIIYHSGPDQVEPQDPKGLEQGGGMMEPGV
>120.Kalanchoe laxiflora SST1
MQRRLLFALFTLFCASELYLKQVCEAVDGRRELSSYWRDVMKDEDMPEAIQGLVLGDGQK
MQETSFDREFQARPNLIIYHSGPDQVEPQDPKGLEQGGGMMEPGV
>121.Cucumis sativus SST1
MKLSIAFFFLLSLLLLGDLTHGRSRKEEGEYWKKIMKEEALPEMLKELLIEADDPSSMVE
YNNNKQQKEHFLTNFDPHPNAIIYHAHAHAHNSNPTALPNLSP
>122.Cucurbita maxima SST1
MKLSAAFFLLSLLLLANVSHARKEKGEYWKKVMKDEAIPEMLKELLFDDDSLVSSDAQSE
RFMNNFDTHPNAIIYHSHGATHDHPGHKTKLTAP
>123.Vaccinium darrowii SST1
MRSPSAAIFLLALLLLVTNFGNARKDPAANYWKRVMKGEPMPKAIEEVLVHDHGAEISES
KEKKRWDPSQINMAHFMRDFDTSPNVIIYHSHNNMGRQTTPEKSPAKIIQAAGGEEISE
>124.Vaccinium darrowii SST2
MRSLSAAILLLSLLFLVAKFGDARKDPIAADYWKSMMRGSPIPRAIEEVLVHDQDGETSV
SKQKEKKLWDPSQTNMAHFRRDFETTHNVLIYHSHNNMGHGTTP
>125.Vaccinium darrowii SST3
MRALSAFFLLSLLFLFSSSCDARKDPADYWKSIMKGEPMPEKIQRLVFHQDPSSLSKEKI
KKWCSSSAIINMEHFRKDFDTSPNLIIYHSGAEPRN
>126.Vaccinium darrowii SST4
MKREGGNTMKKFSSSYDARKDPADYWKSIMKGDPMPEKIQQLVFLQDPSSLSEEKIKKCC
SSSDSINTDHFRKDLDTSPNLISYHSRAEPRN
>127.Vaccinium darrowii SST5
MRSLSAAAILLLSLLFLVAKFGDARKDPIAADYWKSMMKGKPMPKAIEEVLVHDHDGETS
VSKQKEKKMLDPSQINMAHFKMDFETTRNELIYHSLNKP
>128.Jatropha curcas SST1
MIKPTFILLLFLFVNLNHGRELPEDYWKSIMKEERMPEAIRDLLVEGPEASQPAGAKRIK
HFVKDFDTRSIAVIYRKLSEPEKITTEDYKKKGDEYSFVELNKPEMEFHTYHRDHKLKI
>129.Manihot sculenta SST1
MKLTLGVGFFLLLSVLSFCNLNYARNLPEDYWKSIMKDQKIPEAIRGMFVEDPASSSSSS
SGGNVKSHFVKDFDTRTIAVIYRSYGDNKKMNSPHVEESRDDKIKGEKPLVDQHKNPETE
VPVDSRNHKGKV
>130.Manihot sculenta SST2
MKPTHAFFLVFLLISIVNLNYARKVPEDYWKVVMKDQPIPEAIKNLFVEEDEEAAASANK
KNHFVSDFDTRAVAVIYRSHGDINKKMNMLR
>131.Manihot sculenta SST3
MKSFSDALLPIFFFLLIFSITDARKDLGEYWREVMKDQLLPEPIQELLQASPASSASHEK
NDCRIISKERSH
>132.Ricinus comunis SST1
MKHMFTLFLLLSVFLQFFNLNYARKLPAEDYWKSVMREQPMPKAIKDLFVQDPEAASLSS
TGSNKKTHFVKNFDTRSIAVIYRRQDESKKARKEFKEDKMKEEKPFSEHSKTGMEVPT
>133.Castanea dentata SST1
MKSILVLAIFFLFSLLLFVNPNDARKDTRDYWKSIMKDQPIPEAIKELFHRDPPYLFDAT
KKDHFVLDFDARPNAIIYHAKEEKPNIKDFEPNYHGSELKEE
>134. Quercus rubra SST1
MDSVDLLVCFLLLSYNGVEAARGLYQTKLEIQKQFTNLKGSTIENTHSVNGNDAKKDPEF
YWKSIMENQPMPEAIKELFTKDPLYLSDARKNNHFVKDFDTRHSAIIYHARDELKE
>135. Quercus rubra SST2
MKSILVLAIFFLFSLLLFVNPNDARKDTGDYWKSIMKDQPIPEAIKELFHQDPPYLFDAT
KKDHFVLDFDARPNAIIYHAKEEKPNVKDFEPNYHGSELKEE
>136.Quercus suber SST1
MKSILVLAIFVLFSLLLFVNPNDARKDTGNYWKSIMKDQPIPEAIKELFHQDPPYLFDAT
KKDHFVLDFDARPNAIIYHAKEEKPNVKDFEPNYHGSELKEE
>137.Dorcoceras hygrometricum SST1
MGMRALSAFFLLSILSFAYIADARKDGGNYWKSKMKGEPMPKAIQDLFNQNTSSEMRTTD
RFVRNFEAKRTFIVYHSPTGVHPDEP
>138.Hydrangea quercifolia SST1
MRPLSAFSALLLLFLLISLVDARTDPGEYWKNIMKGETMPKAIQDLLHHQDLTGKGIDKE
LFIKDFDTKTSGIIYHSHVEPKEVKTFFKHVTEPEAEKSANLVKPKANGY
>139.Carya illinoinensis SST1

MKTISAFLLLFSLFLLVDLNDARKDPEDYWKSIMKDQPIPEAIKGLLRRDLPYASDAREK
DHFLKDFDVTPNAIIYHAHVEDKKEKKPCVEDFEQKSYTELNLFEG
>140.Juglans regia SST1
MKSISAFLLLFSLFLLVLVNLSDARRDPGDYWKSIMKDQPIPEAIKGLLHRDLPYASDAR
ENDHFLKDFDVTPNAIIYHAHVEDKDEKKPACVEDFEQKSHTELNLIQG
>141.Linum usitatissimum SST1
MKPCSFTLSCLLLSLLLATATVSEAIRGDPPAAEEYWKKVMKNEPLPSSIKELFNDAAVS
SSSGDEGKKLRFVKDFDTTTNAIIYHAAAAEEQTVLP
>142.Linum usitatissimum SST2
MSEAIRGDPPAAEEYWKKVMKNEPLPSSIKELFNDAVVSSSDFDGKKLRFVEDFDTTTSA
IIYHAATAEEQTALP
>143.Punica granatum SST1
MMKPFCAFIIPFSLLLLLSFTHARKDPAGDYWKKIMKDQPMPEAIRDLIVRPRSKDNHHF
VKDFDVRRSVIIYPARVDPKDNMSSDTEDLKSGHNDASSTQHVNLKVPDQEQKNHENGA
>144.Gossypium arboreum SST1
MKTFIAILIFCFLLLFANLNHARKEPGEYWRSVMKDQRMPEAIKGLLHEDETGSGSGAEM
KMKQFVKDFDSRHSLIIYHNSPESKQEDTTHAKDVKHTKDQKQDKSDRKN
>145.Gossypium barbadense SST1
MKTFIAILIFCFLLLFANLNHARKEPGEYWRSVMKDQPMPEAIKGLLHEDETGSGSGAEM
KMKQFVKDFDSRHSLIIYHNSPESKQEDTTHAKDVKHTKDQKQDKSDRKN
>146.Gossypium darwinii SST1
MKTFIAILIFCFLLLFANLNHARKEPGEYWRSVMKDQPMPEAIKGLLHEDETGSGSGAEM
KMKQFVKDFDSRHSLIIYHNSPESKQEDTTHAKDVKHTKDQKQDKSDRKN
>147.Gossypium hirsutum SST1
MKTFIAILFFCFLLLFANLNHARKEPGEYWRSVMKDQPMPEAIKGLLHEDETGSGSGAEM
KMKQFVKDFDSRHSLIIYHNSPESKQEDTTHAKDVKHTKDQKQDKPDRKN
>148.Gossypium mustelinum SST1
MKTFIAILIFCFLLLFANLNHARKEPGEYWRSVMKDQPMPEAIKGLLHEDETGSGSGAEM
KMKQFVKDFDSRHSLIIYHNSPESKQEDTTHAKDVKHTKDQKQDKSDRKN
>149.Gossypium raimondii SST1
MKTLFFFFIFCSFLLYGNLNHARKEPRDYWKSVMKDQPIPEAIQGLLHQDEASAMDSENF
VKDFDSRHSFIIYHSNLKHKEEEDKTYVKDLKNQKEHKSDKKNQTEKY
>150.Gossypium raimondii SST2
MKTSIAILIFCFLLLFANLNHARKEPGEYWRSVMKDQPMPEAIKGLLHEDETGSGSGAEM
KMKQFVKDFDSRHSLIIYHNSPESKQEDTTHAKDVKHTKDQKQDKPERKN
>151.Gossypium tomentosum SST1
MKTFIAILFFCFLLLFANLNHARKEPGEYWRSVMKDQPMPEAIKGLLHEDETGSGSGAEM
KMKQFVKDFDSRHSLIIYHNSPESKQEDTTHAKDVKHTKDQKQDKPDRKN
>152.Gossypium tomentosum SST2
MKTLFFFFIFCSFLLYGNLNHARKEPRDYWKSVMKEQPIPEAIQGLLHQDEASAMDSENF
VKDFDSRHSFIIYHSNLKHKEEEDKTYVKDLKNQKQHKSEKKNQTEKP
>153.Morus notabilis SST1
MKSIFAFFVLFSILLVGNISYARKDAGEYWNSIMKDQPIPEAIRDLFYDQDLPSDLTGPT
KHDRFVRDFDVQPNVIIYHSHAQPQGEDNHKPSVHNHHHEELEETH
>154.Corymbia citriodora SST1
MKPIFAFIFLSSLLLFAEVNDARKSPGDYWKKIMKDQPMPEPIRDLIRLRNEENAIKFVR
DFDTGPNVIIYHSHGDAKEKKHCRENVEEGEDERLKMRDDQDQKEMTSA
>155.Eucalyptus grandis SST1
MKPIFAFIFLLSILLFAEVNDARKSPGDYWKKIMKDQPMPESIRDLIRPGNEKNTNDFMR
DFDMRPNVIIYHSHGDAKEKKHCGENAEKGEDERLKMPDNQDQKKMTSA
>156.Olea europaea SST1
MSLKMNVRTPSTIFLLSLLLLSSVIDARKDPGDYWKSMMNDEPMPKAITDLIHHDTAEES
NHFLRNFDTKPNVIIYHSHVQSAKMKPSPLEG
>157.Sesamum indicum SST1
MKMRSLPLSPLFLLLLSLLLFACVATDARKEPGAYWKSVMNGDEMPKAITDLLHHDHQSS
SATDLNTERDRFIRNFDTKANVIIYHSHDDHHHDHNSKDQRRPMWMV
>158.Portulaca amilis SST1
MKSSLALSVFVLVILQFGSEIEARIGPEEYWKRVMKDEKLPEAITSLLNQDSADPLSQEN
LFHGYFKREFYVDNPDNAIIYHPNHHDTQIPTWSAKRLDSALGKLDPQ
>159.Ziziphus jujuba SST1
MKSLLPFLCILLSLLLFTNLSFARKDIGDYWKSIMKEQPMPEAIRDLFHQEDVPSLPGSR

KMDRFARDFDIRPNLIIYHAHPKPVEGKTETEDEEEFKVIKQINRG
>160.Fragaria vesca SST1
MKYSTSCFLLLLFIFLFSEITYARKDMGDYWKSVMNDQPMPEALKDLFSHQDEDVPSFSA
SKNKDHRFVRDFDIRPNVIIYHSAHHHHADHQPEEMMHEMQPKAYIQTVNHG
>161.Malus domestica SST1
MKFSISCLLLILSSLLLLSQNSYARKDSGGYWKSVMNDQPMPEAIKGLFVHHEQEDQVPS
KEKSHFVRDFDMRPNVIIYHGAHHHHQDQPAEKKPFFQETSYIQTVNHG
>162.Malus domestica SST2
MKFSISCFLLILSSILLMSQNSYARKDSGDYWKSVMNDQPMPEAIKDLFVHQDHEHQEPS
KEKSHFVGDFDMRPNAIIYHGAHHHQVPSTENRHFVRDFDMRPNVIIYHGVHHHHQDQPA
ENKPFFKKASYIQTVNHG
>163.Prunus avium SST1
MKFSISCLLLILSSILLLSQQSNARKDLGGYWKSVMNDQPIPEAIGDLYFHQDHEDHLPS
LPGSREKDHFVRDFDIRPNAIIYHGAHHHHQQQQPAEDKPMHDMEPKEETSYIETVTHG
>164.Prunus persica SST1
MKFSISCLLLILSSILLLSQQSNARKDLGGYWKSVMNDQPIPEAIRDLYFHQDHEDHLPS
LPGSREKDHFVRDFDIRHNAIIYHGAHHHHHQQQQPAEDKPMHDMEPKEETSYIETVTHG
>165.Coffea arabica SST1
MRPYSAFLVLFLLVSFTFGIDARRCPGDYWKSVMNEEPIPEVLSDILHQDTTSEPCEKET
VDTDRLARDFDMRSSVIIYHRDADSKRVKTFDAEGVKMRDNIDSAQSESRQKVLRAE
>166.Coffea arabica SST2
MRSCLVILLFFSVVSLVCISEAARHGPEDYWKKIMQDEQMPKALTDLFHEDSSRSDDHHH
RRHSDINVQKQPETLNMKRFLTNFDTAPTLIIYQNKVVIH
>167.Coffea canephora SST1
MRPYSAFLVLFLLVSFTFGIDARRCPGDYWKSVMNEEPIPEVLSDILHQDTTSEPCEKET
VDTDRLARDFDMRSSVIIYHRDADSKRVKTFDAEGVKMRDNIDSAQSESRQKVLRAE
>168.Citrus clementina SST1
MKSIFASSIIISLLLFASLGYARKEPGDYWKSIMKDKPMPKALKDLFPQADERMVKMDHF
VKDFDRKSSSVSIIYHRRSEPETEKQKHQEDKSLKTEPKFPSAYSQEAKENSNQLHN
>169.Citrus sinensis SST1
MKSIFASSIIISLLLFASLGYARKEPGDYWKSIMKDKPMPKALKDLFPQADERMVKMDHF
VKDFDRKSSSVSIIYHRRSEPETEKQKHQEDKSLKTEPKFPSAYTQKAKENSNQLHN
>170.Poncirus trifoliata SST1
MKSIFASSIIISLLLFASLGYARKEPGDYWKSIMKDKPMPKALKDLFPQADERMVKMDHF
VKDFDRKSSSVSIIYHRRSEPETEKQKHQEDKSLKAEPKFPSAYTQKAKENSNQLHN
>171.Poncirus trifoliata SST2
MRRLFVLCTLSVHLLLASTIDARKDLEEYWRIVMKGQPMPESIQDLLAVDRAASNTNENA
DQKSNPLILSDNAQPSEVESLAKNFNPRPNVLAYSFCW
>172.Populus deltoides SST1
MKSFLAVLVLVSFLSFVELRDARKEPREYYWKSMTKDQPMPEAIKDLFVRDPAGAGKLNH
FVKDFDTRHSAIIYHSRDGKDELKETNPTNARDHEEDKAHAP
>173.Populus deltoides SST2
MKSFLFFLVLLSFLSFVELNHARKEPRENYWKSMTKDQPIPGAIRDLFVQDPAAGADKMN
HFVKDFDTKHNAIIYHSHEKDKLKEKKSMNPTNTWDHEKEKE
>174.Populus euphratica SST1
MKSFLAVLVLVSFLSFVELRDARKEPREYYWKSMMKDQPMPEAIKDLFVQDPAGAGKLGH
FVKDFDTRHSAIIYHSHDEKDELKETNPTNARDHEEDKAYAP
>175.Populus trichocarpa SST1
MAEISQGRRSLATIQSTSIKIKFVELRDARKEPREYYWKSMTKDQPMPEAIKDLFVQDPA
GAGKLNHFVKDFDTRHSAIIYHSHDGKDELKETNPTNARDHEEDKAYAP
>176.Populus trichocarpa SST2
MVEISLSLRDLATIQDKVRVAKSKCRILYMKSFLFFLVLLSFLSFVELNHARKEPRENYW
KSMTKDQPIPGAIRDLFVQDPAAGADKMNHFVKDFDTKHNAIIYHSHEKDKLKEKKSMNP
TNTWDHEKEKE
>177.Salix purpurea SST1
MKSFLAVLVLFSFLSFVELSDARKEPREYYWKSMMKDEPMPEAIKELFVEDPAGAGKMSH
FVKDFDTRHSAIIYHSHAEKDRLKERKSTNARDHDGDAQ
>178. Salix purpurea SST2
MCRILYMKSFLFFLLLLSFLSFAELNHARKEASEYYWKSMTKDQPIPEAIRDLLVRDPAG
SDKMNHFVKDFDTKHSAIIYHSPEKDKLKEKNP
>179.Lindenbergia philippensis SST1

MRSSSAFFILLSVLLVVNQINARNNPGENYWKKLMGKEPIPDAIKGLFSTVDSSHKFVKD
FETKENVIIYHKHFDVAGKNKKPSVGK
>180.Mimulus guttatus SST1
MEMKHLSTFFLLSLVFACVTEARPSPGEYWKSRMNGEAMPKALMDVVSDSDAKTSRFVKD
FNTKPNVIIYHSNYHANKHAQAPKPC
>181.Capsicum annun SST1
MRSLSIFLLLLSLTLLGYVTSDGPEEYWNSKMNGDPMPKALRGLLNDQYQNFPTERNKRS
HKFLRDFDMKANIIIYHNDVDIYPKRPRPTAEEAERRKTVDP
>182.Nicotiana attenuata SST1
MRSLSIFFLFLSLTWFGDATSDPEEYWKSVMNGDPMPKALTDLLHNQYQDFPVERNKDRF
LRDFDLKPNIIIYHNDVDIYPKRPRPTATDDNFSDTKEAERKEPVNPEN
>183.Nicotiana sylvestris SST1
MSSVYFNMRSLSIFFLLFSLTWFGYARSDPEEYWKSVMNGDPMPKALTDLLHNQYQDFPV
ERNKDRFLRDFDLKPNIIIYHNDVDIYPKRPRPTAKDDIFSENKEAERREPVNPGN
>184.Nicotiana tabacum SST1
MSSVYFNMRSLSIFFLLFSLTWFGYARSDPEEYWKSVMNGDPMPKALTDLLHNQYPDFPV
ERNKDRFLRDFDLKPNIIIYHNDVDIYPKRPRPTAKDDIFSENKEAERREPVNPGN
>185.Solanum lycopersicum SST1
MRSLSIFFLLLSLSLLGYVISDGPEEYWKSKMNGDPMPKALKELLNDQYQDFPIERNKFV
RNFDLKANIIIYHNDVDIYPKRSRPTP
>186.Solanum pennelli SST1
MRSLSIFFLLLSLSLLGYVISDGPEEYWKSKMNGDPMPKALKELLNDQYQDFPIERNKFV
RNFDLKANIIIYHNDVDIYPKRSRPTP
>187.Solanum tuberosum SST1
MKSLSIFFLLLSLSLLGYVISDGPEEYWKSKMNGDPMPKALKDLLNDQYQDFPIERNKFV
RNFDLKANIIIYHNDVDIYPKRSRPTP
>188.Solanum tuberosum SST2
MKSHIALILLFSLALYTDARKDPGEYWRDVMKDEPMPKAIQHLMPQPDKEKIDSHKSSFE
PIPNASSFIE
>189.Herrania umbratica SST1
MKTFFAIFIFCFLLLSANPNHARKEPGDYWKSVMKDQPMPEAIKGLLHQDPASALGSEKN
MKHFVTDFDTRHSAIIYHSGPQSKVEDNPQVKDLKDQKQQKSDKKN
>190.Theobroma cacao SST1
MKTFFAVFIFCFLLLSANPNHARKEPGDYWKSVMKDQPMPEAIKGLLHQDPASALGSEKN
MKHFVKDFDTKHSVIIYDSGPQSKVEDNPHVKDLKDQKQQKSDKKN
>191.Corchorus olitorius SST1
MKTLILAIFLFCFLLLSANLNYARKEPGEYYWKSVMKDQPMPEAIKGLFHDQDPASSSSA
LGSDKKMNTFVKDFDSRHSVIIYHTSPVSEKEESKHSVKDLKP
>192.Parasponia andersonii SST1
MKSITAFVVLCSILLLGNLSYARKDAGDYWKSIMKNQPMPDAIKDLFHEEGLPSLPPGSA
KRDRFVKDFDVRPNVIIYHSSHSQPEGVLDDHMPSVHEFQTKHHQQLQQVINQIRRD
>193.Vitis vinifera SST1
MSQLVSKQMKFTFPFFISFLLLLFSGLIDGRKDMGDYWKSIMKGQPMPEAIKGFIHQDPA
SFSSKARKMDHFVRDFDARPNNIIYHGHVERKGEKPLGVEMKPELKKEKFFVEPVNLRVN
FHKHGHDQKEYNN
>194.Vitis vinifera SST2
MASSLAFFTIFSLLLVGNAIGGRKEPGVYWRDVMKDEPMPKAIQGLLPEDQSSSLLSKKP
NCQTIPGARNGGDIVKGFEPKKEKVFWVYDDEDAKLTEEKSFVKDLEPRTNVSA
