# Supplementary material for: A Comprehensive Analysis of Short Specific Tissue (SST) Proteins, a New Group of Proteins from PF10950 That May Give Rise to Cyclopeptide Alkaloids
Source: Plants (Basel). 2025 Apr 3;14(7):1117. doi: 10.3390/plants14071117 (PMC11991032; doi:10.3390/plants14071117)
Supplement: Supplementary file 1 [file plants-14-01117-s001.zip › List S3.pdf]

List S3. List of 194 SST mature protein sequences in FASTA format. The sequence numbers are the same as in Table S1. It must be considered that some mature proteins have been determined by comparison with the canonical sequence because prediction services do not determine the existence of a signal peptide.

>1.Alyssum linifolium SST1
RSGGVAEEYWKKMMKNEPLPEPIKELLNNPFRNTQEIFIQNFDPKSVAIIYRSPKE
>2.Alyssum linifolium SST2
RPARGVAEEYWRKMMKNEPFPEPIKELLNNPFRTAQERFIPNFDTKVLVLIYHNPHHD
>3.Alyssum linifolium SST3
RPARGVAEEYWRKMMKNEPFPEPIKELLNNPFRTAQERFIPNFDTKVLVLIYHNPHHD
>4.Alyssum linifolium SST4
RSEGGVAEEYWKKMMKNEPLPEPIKELLNNPFRNTQEIFIQNFDPKSVAIIYRSPKE
>5.Arabidopsis halleri SST1
RTGGVAEEYWKKMMKNEPLPEPIKELLNNPFRTGQERFIQNFDTKSVVIIYHNPNE
>6.Arabidopsis lyrata SST1
RTGGVAEEYWKKMMKNEPLPEPIKELLNNPFRTGQERFIQNFDTKSVVIIYHNPNE
>7.Arabidopsis lyrata SST2
RTGGVAEEYWKKMMKNEPLPEPIKELLNNPFRTGQERFIQNFDTKSVVLIYHNPNE
>8.Arabidopsis thaliana SST1
RTGGVAEEYWKKMMKNEPLPEPIKELLNNPFRTAQERFIQNFDTKSVVIIYHNPNE
>9.Boechera stricta SST1
ESGRGVAEEYWKKMMKNEPLPEPIKELLSNPFRTAQEMFIQDFDTKSVVLLYRNPNK
>10.Boechera stricta SST2
RSGGGGVAEEYWKKMMKNEPLPEPIKELLNNPFRTAQERFIQSFDTKSVVIIYHRPNE
>11.Brassica oleracea SST1
RSGGVAEEYWKKIMKNEPLPEPIKELLNNPFRTGEERFVKDFKTKSIVIIYHNPNV
>12.Brassica oleracea SST2
KSEVAEDYWKKMMKSEPLPEPIKDILNNPFRTGQERFAKNFNTKSVVIIYHNPNV
>13.Brassica rapa SST1
RSGGVAEEYWKKMMKDEPLPEPIKDLLNNPFRTGQERFVKDFNTKSIVIIYHNPNV
>14.Brassica rapa SST2
RSGGVAEEYWKKIMKNEPLPEPIKELLNNPFRTGEERFVKDFKTKSIVIIYHNPNV
>15.Brassica rapa SST3
KSEVAEDYWKKMMKSEPLPEPIKDILNNPFRTGQERFAKNFNTKSVVIIYHNPNV
>16.Cakile maritima SST1
RSGGVAEEYWKKIMKNEPLPEPIKELLNNPFRTGEDRFVKDFKTKSIVIIYHNPNV
>17.Cakile maritima SST2
RSGGVAEEYWKKMMKNEPLPEPIKELLKNPFRTGEERFVKDFNTKLVVIIYHNPNV
>18.Cakile maritima SST3
KSGVAEEYWKKMMKNKPLPEPIKDFLNNPFRTGPERFVKNFNTKSVVIIYHNPNV
>19.Camelina sativa SST1
RSGGGGVAEEYWKKMMKNEPLPEPIKELLYNPFRTAEERFIQSFDTKSVVIIYHNPKE
>20.Capsella grandiflora SST1
RSGGGVAEQYWKKMMKNEPLPEPIKELLYNPFRTVDERFIQSFDTKSVVIIYHNPNE
>21.Capsella grandiflora SST2
GRSGGRDGVAEEYWKKMMKNEPLPEPIKELLKNPFRTAQEMFIQDFNTKSVVLIYHNPHE
>22.Capsella rubella SST1
RSGGGVAEQYWKKMMKNEPLPEPIKELLYNPFRTVDERFIQSFDTKSVVIIYHNPNE
>23.Capsella rubella SST2
GRSGGRDGVAEEYWKKMMKNEPLPEPIKELLKNPFRTAQEMFIQDFNTKSVVLIYHNPHE
>24.Caulanthus amplexicaulis SST1
RSGGVAEEYWKKMMKNEPLPEPIKELLNNPFRTGQERFVKNFNTKSIVIIYHNPNV
>25.Caulanthus amplexicaulis SST2
RSGGVAEEYWKKVMKNEPLPEPIKELLNNPFRIGQERFVKDFNTKSIVIIYHNPNV
>26.Cleome violacea SST1
RREAEDYWKKTMKDEPLPEPIKDLLNNPFRTDQKFIHNFDTKSLALIYHKN

>27.Cleome violacea SST2
RMEPEEYWKKAMAEAELPEPIKELMNNPFRSKHEKFINNFGTKATVLIYHNPNP
>28.Crambe hispanica SST1
RSGGVAEEYWKKIMKNEPLPEPIKELLNNPFRTGEERFVRDFKTKSIVIIYHNPNV
>29.Crambe hispanica SST2
KSGVAEEYWKKMMKNEPLPEPIKDFLNNPFRAGQERFAKNFNTKSIVIIYHNPNI
>30.Descurainia sophioides SST1
RSEGGVAEEYWKKMMKNEPLPEPIKELLNNPFRNTQEIFIQNFDPKSVAIIYRSPKE
>31.Descurainia sophioides SST2
RPARGVAEEYWRKMMKNEPFPEPIKELLNNPFRTAQERFIPNFDTKVLVLIYHNPHHD
>32.Diptychocarpus strictus SST1
RSGGGGVAEKYWKKVMKNEPLPEPIKELLNNPFRTAKERFIKNFNTKSVVIIYHNPNE
>33.Euclidium syriacum SST1
RPGEGVAEKYWKKVMKNEPLPEPIKELLNNPFRTAQERFIKNFKTKSIVIIYHNPNE
>34.Eutrema salsugineum SST1
RSGGVAEEYWKKVMKNEPLPEPIKELLNNPFRTAKERFVKNFNTKSIVIIYHNPNV
>35.Eruca vesicaria SST1
KSGVPEDYWKKMMKKEPLPEPITDILNNPFRKGQERFVKNFNTKSIVIIYHNPNA
>36.Eruca vesicaria SST2
RSGGVAEEYWKKIMKNEPLPEPIKELLNNPFRTGEDRFVKDFKTKSIVIIYHNPNV
>37.Iberis amara SST1
RSGGGVAEEYWKKMMKNDPLPEPIKELLNNPFRTEKERFITNFNTKSILIIYHDPLV
>38.Iberis amara SST2
RSGREVIEEYWKKMMKNEPLPEPIKQILNNPFRTAQEKFVTNFDTHSVLIIYHNPIE
>39.Isatis tinctoria SST1
RSGGVAEEYWKKMMKNEPLPEPIKELLNNPFRTGDERFVRDFNTKSIVIIYHNPNV
>40.Lepidium sativum SST1
RSGREVIEEYWKKTMKNEPLPEPIKVLLNNPFRTGDERFIQSFDTKSVVIIYHSPKE
>41.Lunaria annua SST1
RSGGGGVAEEYWKKMMKNEPLPEPIKELLHNPFRTEQEMFITNFDTKSVVIIYHNPNE
>42.Malcolmia maritima SST1
RPRGGVQEEYWKKMMKNEPLPEPIKELLYNPFRTGQGLFTKDFDTKSAVLIYHNPNE
>43.Malcolmia maritima SST2
RSGGVAEKYWKKIMKNEPLPEPIKELLYNPFRTGDERFIQSFDTKSVVIIYHTPNE
>44.Myagrum perfoliatum SST1
RSGGVAEEYWKKMMKNEPLPEPIKELLNNPFRTGEERFVKDFNTKSIVIIYHNPNV
>45.Raphanus sativus SST1
RSGGVAEEYWKKIMKNEPLPEPIKELLNNPFRTGEERFVKDFKTKSIVIIYHNPNV
>46.Rorippa islandica SST1
RPGGVAEEYWKKMMKNEPLPEPIKELLNNPFRTAQEKFIQNFDTKSVVIIYHNPKE
>47.Schrenkiella parvula SST1
RSGGVAEEYWKKMMKNEPLPEIIKELLNNPFRTGRERFVKNFNTKSIVIIYHKPNV
>48.Sinapis alba SST1
RSGGVAEEYWKKIMKNEPLPEPIKELLNNPFRTGEERFIKDFKTKSIVIIYHNPNV
>49.Sinapis alba SST2
RLGGVAEEYWKKMMKDEPLPEPIKDLLNNPFRTGQERFVKDFKTKSIVIIYHNPNV
>50.Sinapis alba SST3
KSGVAEEYWKKMMKSEPLPEPIQDLLNNPFRTGQERFVKNFNTKSIVIIYHNPNV
>51.Sinapis alba SST4
KSGVAEEYWKKMMKSEPLPEPIQDLLNNPFRTGQERFVKNFNTKSIVIIYHNPNV
>52.Stanleya pinnata SST1
RSGGVAEEYWKKMMKNEPLPEPIKDLLNNPFRTGQERFVKNFNTKSIVIIYHNPNV
>53.Stanleya pinnata SST2
RSGGVAEEYWKKVMKNEPLPEPIKELLNNPFRIGQERFVKDFNTKSIVIIYHNPNV
>54.Thlaspi arvense SST1
RSGGGVAEEYWKKMMKNEPLPEPIKELLDNPFRTARERFVKNFNTKSIVIIYHNPNV
>55.Arachis hipogea SST1
RKDQGEYWKDMMDKQTMPEAIKDLLVEDPQVSSSHARKNKDQFRKDFDIKPNVILYHSHV
GPKKQKA
>56.Arachis ipaensis SST1
RKELVGEYWKKMIKGQSMPEAIKEILVVEDPQISSSDSSRTKDNFIRDFDIKPNVILYHS

HKQKQKHNHKNPFLNNNLEEPEFQETENK
>57.Cajanus cajan SST1
RKDLGAYWKNMMKGQAMPQAIKDLVEASDAVDAGTKNRFVRDFDVKPNVILYHTHVKSSK
QKQKLFLNNNQD
>58.Cicer arietinum SST1
RKDLVDYWKNMMNDQPMPETIKELVQNPQVTDAGNDKFIRNFDIRPNVILYHTHVDSNKK
QHVFVNNSEQDHFHGITRKHG
>59.Cicer arietinum SST2
RKDVGDYWMNMMNEQAMPEAIKNLVQVPIVSNKRKEDRFITDFDVHPNIILYHTHVHQKQ
P
>60.Cicer arietinum SST3
RKDVGEYWKNKMNEQAMPEAIKNLVQVQKQDHFVRDFDVHPNIILYHTHVHEKQEKPLAP
>61.Glycine max SST1
RKDLGWYWKNVMKEQPMPQAIKDLVEDSQASAAGKKDRFIRDFDVKPNVILYHTHVVPMK
QKHKHKQNPFVKNQD
>62.Glycine max SST2
RKDLGGYWKNMMKEQPMPQAIKDLVEDSQASDTGKKDLFTRDFDVKPNVILYHTHVVSMK
QKQKPFLQN
>63.Glycine max SST3
RKDMGDYWKNMMNGQPMPEAIKDLLVQDPQVSDAMKDHFIRDFDIKPNVILYHTHVVPHK
HKQKIQQAMAKKLEPEFQGTERHG
>64.Glycine max SST4
RKDMGDYWKNMMNGQPMPEAIKDLLVQDPQVSDAVKDHFIRDFDIKPNTILYHTHVVPNK
QKQKQNIQQAMAKKLEPKFQGTERHG
>65.Glycine max SST5
RKDLGDYWKKMMNDQPMPEAIKENCSSSTSIRWKNGSFYKGLQYKA
>66.Glycine max SST6
RKDLGDYWKKMMNDEPMPEAIKDLIQDQQVQDATADHFIRYFDMKPNIILYHTHVVSKKQ
QQQKAFDHKFKPVSRNGKSWLNKP
>67.Glycine max SST7
RKDLGDYWKKMMNDQPMPEAIKENCSSSTSIRCKNGSLYKGLQYKA
>68.Glycine soja SST1
RKDLGGYWKNMMKEQPMPQAIKDLVEDSQASDTGKKDLFTRDFDVKPNVILYHTHVVSMK
QKQKPFLQN
>69.Glycine soja SST2
RKDLGWYWKNVMKEQPMPQAIKDLVEDSQASAAGKKDRFIRDFDVKPNVILYHTHVVPMK
QKHKHKQNPFVKNQD
>70.Glycine soja SST3
RKDMGDYWKNMMNGQPMPEAIKDLLVQDPQVSDAMKDHFIRDFDIKPNVILYHTHVVPHK
HKQKIQQAMAKKLEPEFQGTERHG
>71.Glycine soja SST4
RKDLGDYWKKMMNDEPMPEAIKDLIQDQQVQDATADHFIRYFDMKPNIILYHTHVVSKKQ
QQQKAFDHKFKPVSRNGKSWLNKP
>72.Glycine soja SST5
RKDLGDYWKKMMNDQPMPEAIKENCSSSTSIRCNNGSLYKGLQYKA
>73.Glycine soja SST6
RKDLGDYWKKMMNDQPMPEAIKENCSSSTSIRCKNGSFYKGLQYKA
>74.Glycine soja SST7
RKDMGDYWKNMMNGQPMPEAIKDLLVQDPQVSDAVKDHFIRDFDIKPNTILYHTHVVPNK
QKQKQNIQQAMAKKLEPKFQGTERHG
>75.Lotus japonicus SST1
RKDLGDYWKDMMNGQPMPEAIKDLLVEDPQVSDAAGKDHFTRDFDIRPNVILYHTHVESK
KQNQKQKKQLFVKNFQLPEFEGITGSHG
>76.Lotus japonicus SST2
RKDMGGYWKNVMNGQPMPEVVKDLIQDPHASDAGKDHFIRDFDIKPNNVSIFHTHVVPTS
RSRRHLTRNLN
>77.Lotus japonicus SST3
ATRDLGDYWKNMMKGQAMPEAIKELVQDPQASYAGKDRFIRDFDIRPNVILYHTHVGSST
KQKQNTFAKN
>78.Lotus japonicus SST4
RKDMGEYWKNVMNDQPMPEVVKDLIEDPQVSDAGKDHFIRDFDIKPNNATIFHTHVVPTS

RSRRHLTRNLN
>79.Lupinus albus SST1
RKDVEDYWKNKMKGQPMPEPIKDLTYDLVAASDAGKSRFIRDFDIKPNVILYHTHVVSKE
QKQKNPFVKKIETKFKKPEVMVVEQTVKKD
>80.Lupinus albus SST2
IKHEGEYWKNIMKDQPMPETIKDLLVQDPQVLDSGKDHFIRDFDIRPNVILYHTHVVSKK
QKQHPFVNNFEPEFQE
>81.Lupinus angustifolia SST1
RQHQGEYWKNIMKDQPMPETIKDLLVQDPQAYTEKYHFIRDFDIRPNVILYHTHVVSKKQ
KQHPFVKNFEPEFQEIGTRV
>82.Medicago truncatula SST1
RKDLGDLWKNKMNKQPMPEAIKNFIQVPKALGEGKEDHSFTTDFDVNPNIILYHTHVHQD
EKPFEHAARKMESLLPKRG
>83.Medicago truncatula SST2
TKENMVEYWKNMMKGEAMPEAIKGLNSGSTSNICREWTLM
>84.Medicago truncatula SST3
RKDLGGYWKNMMNDQPMPEAIKELVQNQEVSDDFIRDFDVKPNVILYHTHVESKKKKEHV
FVKNSQQELHGT
>85.Medicago truncatula SST4
RKDLGDYWKNKMNEQPMPEAIKNLIQVPKALDEGKEDHSFTTDFDVNPNIILYHTHVHQD
EKPFQHAARKMEPLLPKRG
>86.Phaseolus acutifolius SST1
RKDMRGYWKDMMKEQPMPQAIKDLIEDSEVSEAGKGRFVRDFDVKPNVILYHTHVVPMKQ
RQKNQD
>87.Phaseolus acutifolius SST2
RKDMGDYWKNMMNGQPMPEAIKDLLVQDPQVSDTVKDHFIRDFDIRPNVILYHTHIPPNK
RKQHAMAKKIEEFHGTGN
>88.Phaseolus coccineus SST1
ARKDMRGYWKDMMKEQPMPEAIKDLIEDSEEVSEAGKGRFVRDFDVKPNVILYHTHVVPM
KQRQKNKD
>89.Phaseolus lunatus SST1
RKDMRGYWKDMMKEQPMPQAIKDLIEDSEASEAGKGRFVRDFDVKPNVILYHTHVVPRKQ
RQKKQD
>90.Phaseolus lunatus SST2
RKDMGDYWKNMMNGQPMPEAIKDLLVQDPQVSDTVKDHFIRDFDIRPNVILYHTHIPPNK
RKQHAMAKKIKEFHGTGRHA
>91.Phaseolus vulgaris SST1
ARKDMRGYWKDMMKEQAMPEAIKDLIEDSEEVSEAGKGRFVRDFDVKPNVILYHTHVVPM
KQRQKNKD
>92.Phaseolus vulgaris SST2
RKDMGDYWKNMMNGQPMPEAIKDLLVQDPQVSDTVKDHFIRDFDIRPNVILYHTHIPPNK
RKQHAMAKKIEEFHG
>93.Trifolium pratense SST1
RKDLDDFWKNMMNDQPMPEAIKELVQNTKVIDSRKDNFIRDFDVKPNLILYHTHVESKKQ
KRKQHIFVKKSEQEEFHGTQKHG
>94.Trifolium pratense SST2
RKDPGEYWKNMMKGEAMPEAIKELIQDPQAIYAGKDGFMRDFDVKPNAILYHTHVMSMEQ
TRRC
>95.Trifolium pratense SST3
RKDLGDYWKNKMNEQPMPEAIKNLIQVPKALSSNEEKDNSFNRDFDVHPNVILYHTHVHE
EKKPFDESAVRKMESLLPNKG
>96.Vigna angularis SST1
RKYMGDYWKNMMNGQPMPEAIKDLLVQDPQESDAAAVKDHFIRDFDIRPNIILYHTHDMS
RKQKQHAMAKKIEELQEPGRHG
>97.Vigna unguiculata SST1
RKDLDGYWKETMKEQPMPEAIKDLIEDSQVSETGKDRFIRDFDVKPNVILYHTHVVSMKQ
TQKNQD
>98.Amaranthus hypocondriacus SST1
RKGPEEYWKEIMKDQALPEAINDIIDPKRVNPSEKNIILDSF
>99.Amaranthus hypocondriacus SST2
RREPGEYENDMMMKEKVIPEAIKDFILYDKLYDAHKLAQAIKDISEDFDSTPNATIYHDD

ADQE
>100.Anacardium occidentale SST1
RKEPGDYWKSLMKEQPMPKAIKDLLHQDSEGRKIDHFAKDFDVNPNVIIYHSHSEPKQER
QEEKSLVNQIKSQRG
>101.Daucus carota SST1
RPSPRQYWHVTKGEAMPMFQDFDTMNYDAGLLYHGSALAEDKFVQDSVHEKLDFGDDRND
SYLKRKSSDESG
>102.Cynara cardunculus SST1
RKGPEEYWRSIMKDEPMPKAIQDVLSEDSTDKENNRDRFTRDFDTKPNLIIYHSHVMYNQ
KDHELASSKIN
>103.Helianthus annuus SST3
RMGPEEYWRSVMKDEPMPETLQNVLVHDSSSLEDKEKKKDRFIRNFDTKPNLIIYHSHVM
YNQKGHELASSKLN
>104.Helianthus annuus SST2
RSDPREYWRSVMKDEPMPKTIQDVLPLEDGMKVNKDIFTRNFNLKPNLIIYRSHVVYSEK
NHHIVSSSSSSFDELN
>105.Helianthus annuus SST3
RKDPEEIWRSVMKDEPMPKTIQDALSQDSTRSNKEVNMKDQSVSVFDTQPNHKMFQRDFD
TKPNSMTFLYPKPTL
>106.Helianthus annuus SST4
RKDPKDWRNAMKYEPIPPDALSQDSTTLNDKENNKDQFVRDFDKQPNLKMFAKGFNPKPS
SLVSNGCPPKQD
>107.Lactuca sativa SST1
REGPKEYWRSVMKDEPMPKAIQDVLIQDSARSNNNKDRFTRNFDTKPNLIIYHSHVIYNQ
KDHELASSKMN
>108.Lactuca sativa SST2
REGPKEYWRSVMKDEPMPKAIQDVLIQDSARSNNNKDRFTRNFDTKPNLIIYHSHVIYNQ
KDHELASSKMN
>109.Betula platyphylla SST1
RKDLGGYWKNVMDSQPMPEAIQGRIYPQTGKGVHFAKDFDPVSLVDSNIVWVPPPGDHLI
HPEARKEDHLPRSSSRPKAENSPISKAKP
>110.Handroanthus impetiginosus SST1
RKDPGDYWKSIMNGEPMPKAITDLIHRYNLESDSNMKMDYFIKNFNTKANVIIYHSHHKV
HSDHKPKVSNMKLV
>111.Trema orientalis SST1
RKDAGDYWKSIMKNQPMPDAIKDLFHDEGLPSLPPGSAKRDRFVKDFDVRPNVIIYHSSH
SQPEGVLNDHMPSVHEFQTKHHQQLQQVINKISRD
>112.Carica papaya SST1
RKEAGGEYWGSVMKGQAMPEVVEALTYPNSPTTTQPQQTQPKRESRNFEDAWR
>113.Beta vulgaris SST1
RKNPEEYWKKVMKDQPIPEVINGIIDEEIMAKSYEKETFWKHFKRDFDVNSNVIIYHSHQ
ENNHLSPST
>114.Chenopodium quinoa SST1
RNSPEDYWKKVMKDQPIPEAINGIIDQKMANASEKNSLWSHFKRDFDVTSNVIIYHPHQV
NNHRSPSD
>115.Spinacea oleracea SST1
RNSPEDYWKKIMKDQPIPEAIGGIIDQEMANPSEKKPFWSHFKRDFDVTSNVIIYHPHQE
NTMRSPSA
>116.Ipomoea nil SST1
RRDPGEYWDAMMNGDPMPKAITDLLLINQDPSSSSSSPNDRFIRDFDTKPNLIIYHSHVD
VYPKKHEVVAKDVQQKKT
>117.Kalanchoe fedtschenkoi SST1
AGGRKELSSYWRNVMKDEDMPEAIKGLVVAGDGQELQREFRPRPNLIIYHNGPDHRQKHW
RLEDVKGLQQQDSATEPGV
>118.Kalanchoe fedtschenkoi SST2
RKDSGDYWKGVMKDEPMPAALEALILPIPNTSSSQSSLVKKPDCRHEDPTASHVKGKTFG
YDIEPRPSVTVRPQVQQRIK
>119.Kalanchoe fedtschenkoi SST3
VDGRRELSSYWRDVMKDEDMPEAIQGLVLGDGQKMQETSFDREFQARPNLIIYHSGPDQV
EPQDPKGLEQGGGMMEPGV
>120.Kalanchoe laxiflora SST1

VDGRRELSSYWRDVMKDEDMPEAIQGLVLGDGQKMQETSFDREFQARPNLIIYHSGPDQV
EPQDPKGLEQGGGMMEPGV
>121.Cucumis sativus SST1
RSRKEEGEYWKKIMKEEALPEMLKELLIEADDPSSMVEYNNNKQQKEHFLTNFDPHPNAI
IYHAHAHAHNSNPTALPNLSP
>122.Cucurbita maxima SST1
RKEKGEYWKKVMKDEAIPEMLKELLFDDDSLVSSDAQSERFMNNFDTHPNAIIYHSHGAT
HDHPGHKTKLTAP
>123.Vaccinium darrowii SST1
RKDPAANYWKRVMKGEPMPKAIEEVLVHDHGAEISESKEKKRWDPSQINMAHFMRDFDTS
PNVIIYHSHNNMGRQTTPEKSPAKIIQAAGGEEISE
>124.Vaccinium darrowii SST2
RKDPIAADYWKSMMRGSPIPRAIEEVLVHDQDGETSVSKQKEKKLWDPSQTNMAHFRRDF
ETTHNVLIYHSHNNMGHGTTP
>125.Vaccinium darrowii SST3
RKDPADYWKSIMKGEPMPEKIQRLVFHQDPSSLSKEKIKKWCSSSAIINMEHFRKDFDTS
PNLIIYHSGAEPRN
>126.Vaccinium darrowii SST4
RKDPADYWKSIMKGDPMPEKIQQLVFLQDPSSLSEEKIKKCCSSSDSINTDHFRKDLDTS
PNLISYHSRAEPRN
>127.Vaccinium darrowii SST5
RKDPIAADYWKSMMKGKPMPKAIEEVLVHDHDGETSVSKQKEKKMLDPSQINMAHFKMDF
ETTRNELIYHSLNKP
>128.Jatropha curcas SST1
RELPEDYWKSIMKEERMPEAIRDLLVEGPEASQPAGAKRIKHFVKDFDTRSIAVIYRKLS
EPEKITTEDYKKKGDEYSFVELNKPEMEFHTYHRDHKLKI
>129.Manihot sculenta SST1
RNLPEDYWKSIMKDQKIPEAIRGMFVEDPASSSSSSSGGNVKSHFVKDFDTRTIAVIYRS
YGDNKKMNSPHVEESRDDKIKGEKPLVDQHKNPETEVPVDSRNHKGKV
>130.Manihot sculenta SST2
RKVPEDYWKVVMKDQPIPEAIKNLFVEEDEEAAASANKKNHFVSDFDTRAVAVIYRSHGD
INKKMNMLR
>131.Manihot sculenta SST3
RKDLGEYWREVMKDQLLPEPIQELLQASPASSASHEKNDCRIISKERSH
>132.Ricinus comunis SST1
RKLPAEDYWKSVMREQPMPKAIKDLFVQDPEAASLSSTGSNKKTHFVKNFDTRSIAVIYR
RQDESKKARKEFKEDKMKEEKPFSEHSKTGMEVPT
>133.Castanea dentata SST1
RKDTRDYWKSIMKDQPIPEAIKELFHRDPPYLFDATKKDHFVLDFDARPNAIIYHAKEEK
PNIKDFEPNYHGSELKEE
>134. Quercus rubra SST1
ARGLYQTKLEIQKQFTNLKGSTIENTHSVNGNDAKKDPEFYWKSIMENQPMPEAIKELFT
KDPLYLSDARKNNHFVKDFDTRHSAIIYHARDELKE
>135. Quercus rubra SST2
RKDTGDYWKSIMKDQPIPEAIKELFHQDPPYLFDATKKDHFVLDFDARPNAIIYHAKEEK
PNVKDFEPNYHGSELKEE
>136.Quercus suber SST1
RKDTGNYWKSIMKDQPIPEAIKELFHQDPPYLFDATKKDHFVLDFDARPNAIIYHAKEEK
PNVKDFEPNYHGSELKEE
>137.Dorcoceras hygrometricum SST1
RKDGGNYWKSKMKGEPMPKAIQDLFNQNTSSEMRTTDRFVRNFEAKRTFIVYHSPTGVHP
DEP
>138.Hydrangea quercifolia SST1
RTDPGEYWKNIMKGETMPKAIQDLLHHQDLTGKGIDKELFIKDFDTKTSGIIYHSHVEPK
EVKTFFKHVTEPEAEKSANLVKPKANGY
>139.Carya illinoinensis SST1
RKDPEDYWKSIMKDQPIPEAIKGLLRRDLPYASDAREKDHFLKDFDVTPNAIIYHAHVED
KKEKKPCVEDFEQKSYTELNLFEG
>140.Juglans regia SST1
RRDPGDYWKSIMKDQPIPEAIKGLLHRDLPYASDARENDHFLKDFDVTPNAIIYHAHVED
KDEKKPACVEDFEQKSHTELNLIQG

>141.Linum usitatissimum SST1
IRGDPPAAEEYWKKVMKNEPLPSSIKELFNDAAVSSSSGDEGKKLRFVKDFDTTTNAIIY
HAAAAEEQTVLP
>142.Linum usitatissimum SST2
RGDPPAAEEYWKKVMKNEPLPSSIKELFNDAVVSSSDFDGKKLRFVEDFDTTTSAIIYHA
ATAEEQTALP
>143.Punica granatum SST1
RKDPAGDYWKKIMKDQPMPEAIRDLIVRPRSKDNHHFVKDFDVRRSVIIYPARVDPKDNM
SSDTEDLKSGHNDASSTQHVNLKVPDQEQKNHENGA
>144.Gossypium arboreum SST1
RKEPGEYWRSVMKDQRMPEAIKGLLHEDETGSGSGAEMKMKQFVKDFDSRHSLIIYHNSP
ESKQEDTTHAKDVKHTKDQKQDKSDRKN
>145.Gossypium barbadense SST1
RKEPGEYWRSVMKDQPMPEAIKGLLHEDETGSGSGAEMKMKQFVKDFDSRHSLIIYHNSP
ESKQEDTTHAKDVKHTKDQKQDKSDRKN
>146.Gossypium darwinii SST1
RKEPGEYWRSVMKDQPMPEAIKGLLHEDETGSGSGAEMKMKQFVKDFDSRHSLIIYHNSP
ESKQEDTTHAKDVKHTKDQKQDKSDRKN
>147.Gossypium hirsutum SST1
RKEPGEYWRSVMKDQPMPEAIKGLLHEDETGSGSGAEMKMKQFVKDFDSRHSLIIYHNSP
ESKQEDTTHAKDVKHTKDQKQDKPDRKN
>148.Gossypium mustelinum SST1
RKEPGEYWRSVMKDQPMPEAIKGLLHEDETGSGSGAEMKMKQFVKDFDSRHSLIIYHNSP
ESKQEDTTHAKDVKHTKDQKQDKSDRKN
>149.Gossypium raimondii SST1
RKEPRDYWKSVMKDQPIPEAIQGLLHQDEASAMDSENFVKDFDSRHSFIIYHSNLKHKEE
EDKTYVKDLKNQKEHKSDKKNQTEKY
>150.Gossypium raimondii SST2
RKEPGEYWRSVMKDQPMPEAIKGLLHEDETGSGSGAEMKMKQFVKDFDSRHSLIIYHNSP
ESKQEDTTHAKDVKHTKDQKQDKPERKN
>151.Gossypium tomentosum SST1
RKEPGEYWRSVMKDQPMPEAIKGLLHEDETGSGSGAEMKMKQFVKDFDSRHSLIIYHNSP
ESKQEDTTHAKDVKHTKDQKQDKPDRKN
>152.Gossypium tomentosum SST2
RKEPRDYWKSVMKEQPIPEAIQGLLHQDEASAMDSENFVKDFDSRHSFIIYHSNLKHKEE
EDKTYVKDLKNQKQHKSEKKNQTEKP
>153.Morus notabilis SST1
RKDAGEYWNSIMKDQPIPEAIRDLFYDQDLPSDLTGPTKHDRFVRDFDVQPNVIIYHSHA
QPQGEDNHKPSVHNHHHEELEETH
>154.Corymbia citriodora SST1
RKSPGDYWKKIMKDQPMPEPIRDLIRLRNEENAIKFVRDFDTGPNVIIYHSHGDAKEKKH
CRENVEEGEDERLKMRDDQDQKEMTSA
>155.Eucalyptus grandis SST1
RKSPGDYWKKIMKDQPMPESIRDLIRPGNEKNTNDFMRDFDMRPNVIIYHSHGDAKEKKH
CGENAEKGEDERLKMPDNQDQKKMTSA
>156.Olea europaea SST1
RKDPGDYWKSMMNDEPMPKAITDLIHHDTAEESNHFLRNFDTKPNVIIYHSHVQSAKMKP
SPLEG
>157.Sesamum indicum SST1
RKEPGAYWKSVMNGDEMPKAITDLLHHDHQSSSATDLNTERDRFIRNFDTKANVIIYHSH
DDHHHDHNSKDQRRPMWMV
>158.Portulaca amilis SST1
RIGPEEYWKRVMKDEKLPEAITSLLNQDSADPLSQENLFHGYFKREFYVDNPDNAIIYHP
NHHDTQIPTWSAKRLDSALGKLDPQ
>159.Ziziphus jujuba SST1
RKDIGDYWKSIMKEQPMPEAIRDLFHQEDVPSLPGSRKMDRFARDFDIRPNLIIYHAHPK
PVEGKTETEDEEEFKVIKQINRG
>160.Fragaria vesca SST1
RKDMGDYWKSVMNDQPMPEALKDLFSHQDEDVPSFSASKNKDHRFVRDFDIRPNVIIYHS
AHHHHADHQPEEMMHEMQPKAYIQTVNHG
>161.Malus domestica SST1

RKDSGGYWKSVMNDQPMPEAIKGLFVHHEQEDQVPSKEKSHFVRDFDMRPNVIIYHGAHH
HHQDQPAEKKPFFQETSYIQTVNHG
>162.Malus domestica SST2
RKDSGDYWKSVMNDQPMPEAIKDLFVHQDHEHQEPSKEKSHFVGDFDMRPNAIIYHGAHH
HQVPSTENRHFVRDFDMRPNVIIYHGVHHHHQDQPAENKPFFKKASYIQTVNHG
>163.Prunus avium SST1
RKDLGGYWKSVMNDQPIPEAIGDLYFHQDHEDHLPSLPGSREKDHFVRDFDIRPNAIIYH
GAHHHHQQQQQPAEDKPMHDMEPKEETSYIETVTHG
>164.Prunus persica SST1
RKDLGGYWKSVMNDQPIPEAIRDLYFHQDHEDHLPSLPGSREKDHFVRDFDIRHNAIIYH
GAHHHHHQQQQPAEDKPMHDMEPKEETSYIETVTHG
>165.Coffea arabica SST1
RRCPGDYWKSVMNEEPIPEVLSDILHQDTTSEPCEKETVDTDRLARDFDMRSSVIIYHRD
ADSKRVKTFDAEGVKMRDNIDSAQSESRQKVLRAE
>166.Coffea arabica SST2
ARHGPEDYWKKIMQDEQMPKALTDLFHEDSSRSDDHHHRRHSDINVQKQPETLNMKRFLT
NFDTAPTLIIYQNKVVIH
>167.Coffea canephora SST1
RRCPGDYWKSVMNEEPIPEVLSDILHQDTTSEPCEKETVDTDRLARDFDMRSSVIIYHRD
ADSKRVKTFDAEGVKMRDNIDSAQSESRQKVLRAE
>168.Citrus clementina SST1
RKEPGDYWKSIMKDKPMPKALKDLFPQADERMVKMDHFVKDFDRKSSSVSIIYHRRSEPE
TEKQKHQEDKSLKTEPKFPSAYSQEAKENSNQLHN
>169.Citrus sinensis SST1
RKEPGDYWKSIMKDKPMPKALKDLFPQADERMVKMDHFVKDFDRKSSSVSIIYHRRSEPE
TEKQKHQEDKSLKTEPKFPSAYTQKAKENSNQLHN
>170.Poncirus trifoliata SST1
RKEPGDYWKSIMKDKPMPKALKDLFPQADERMVKMDHFVKDFDRKSSSVSIIYHRRSEPE
TEKQKHQEDKSLKAEPKFPSAYTQKAKENSNQLHN
>171.Poncirus trifoliata SST2
RKDLEEYWRIVMKGQPMPESIQDLLAVDRAASNTNENADQKSNPLILSDNAQPSEVESLA
KNFNPRPNVLAYSFCW
>172.Populus deltoides SST1
RKEPREYYWKSMTKDQPMPEAIKDLFVRDPAGAGKLNHFVKDFDTRHSAIIYHSRDGKDE
LKETNPTNARDHEEDKAHAP
>173.Populus deltoides SST2
RKEPRENYWKSMTKDQPIPGAIRDLFVQDPAAGADKMNHFVKDFDTKHNAIIYHSHEKDK
LKEKKSMNPTNTWDHEKEKE
>174.Populus euphratica SST1
RKEPREYYWKSMMKDQPMPEAIKDLFVQDPAGAGKLGHFVKDFDTRHSAIIYHSHDEKDE
LKETNPTNARDHEEDKAYAP
>175.Populus trichocarpa SST1
RKEPREYYWKSMTKDQPMPEAIKDLFVQDPAGAGKLNHFVKDFDTRHSAIIYHSHDGKDE
LKETNPTNARDHEEDKAYAP
>176.Populus trichocarpa SST2
RKEPRENYWKSMTKDQPIPGAIRDLFVQDPAAGADKMNHFVKDFDTKHNAIIYHSHEKDK
LKEKKSMNPTNTWDHEKEKE
>177.Salix purpurea SST1
ARKEPREYYWKSMMKDEPMPEAIKELFVEDPAGAGKMSHFVKDFDTRHSAIIYHSHAEKD
RLKERKSTNARDHDGDAQ
>178. Salix purpurea SST2
RKEASEYYWKSMTKDQPIPEAIRDLLVRDPAGSDKMNHFVKDFDTKHSAIIYHSPEKDKL
KEKNP
>179.Lindenbergia philippensis SST1
RNNPGENYWKKLMGKEPIPDAIKGLFSTVDSSHKFVKDFETKENVIIYHKHFDVAGKNKK
PSVGK
>180.Mimulus guttatus SST1
RPSPGEYWKSRMNGEAMPKALMDVVSDSDAKTSRFVKDFNTKPNVIIYHSNYHANKHAQA
PKPC
>181.Capsicum annun SST1
DGPEEYWNSKMNGDPMPKALRGLLNDQYQNFPTERNKRSHKFLRDFDMKANIIIYHNDVD

IYPKRPRPTAEEAERRKTVDP
>182.Nicotiana attenuata SST1
TSDPEEYWKSVMNGDPMPKALTDLLHNQYQDFPVERNKDRFLRDFDLKPNIIIYHNDVDI
YPKRPRPTATDDNFSDTKEAERKEPVNPEN
>183.Nicotiana sylvestris SST1
RSDPEEYWKSVMNGDPMPKALTDLLHNQYQDFPVERNKDRFLRDFDLKPNIIIYHNDVDI
YPKRPRPTAKDDIFSENKEAERREPVNPGN
>184.Nicotiana tabacum SST1
RSDPEEYWKSVMNGDPMPKALTDLLHNQYPDFPVERNKDRFLRDFDLKPNIIIYHNDVDI
YPKRPRPTAKDDIFSENKEAERREPVNPGN
>185.Solanum lycopersicum SST1
DGPEEYWKSKMNGDPMPKALKELLNDQYQDFPIERNKFVRNFDLKANIIIYHNDVDIYPK
RSRPTP
>186.Solanum pennelli SST1
DGPEEYWKSKMNGDPMPKALKELLNDQYQDFPIERNKFVRNFDLKANIIIYHNDVDIYPK
RSRPTP
>187.Solanum tuberosum SST1
DGPEEYWKSKMNGDPMPKALKDLLNDQYQDFPIERNKFVRNFDLKANIIIYHNDVDIYPK
RSRPTP
>188.Solanum tuberosum SST2
RKDPGEYWRDVMKDEPMPKAIQHLMPQPDKEKIDSHKSSFEPIPNASSFIE
>189.Herrania umbratica SST1
RKEPGDYWKSVMKDQPMPEAIKGLLHQDPASALGSEKNMKHFVTDFDTRHSAIIYHSGPQ
SKVEDNPQVKDLKDQKQQKSDKKN
>190.Theobroma cacao SST1
RKEPGDYWKSVMKDQPMPEAIKGLLHQDPASALGSEKNMKHFVKDFDTKHSVIIYDSGPQ
SKVEDNPHVKDLKDQKQQKSDKKN
>191.Corchorus olitorius SST1
RKEPGEYYWKSVMKDQPMPEAIKGLFHDQDPASSSSALGSDKKMNTFVKDFDSRHSVIIY
HTSPVSEKEESKHSVKDLKP
>192.Parasponia andersonii SST1
RKDAGDYWKSIMKNQPMPDAIKDLFHEEGLPSLPPGSAKRDRFVKDFDVRPNVIIYHSSH
SQPEGVLDDHMPSVHEFQTKHHQQLQQVINQIRRD
>193.Vitis vinifera SST1
RKDMGDYWKSIMKGQPMPEAIKGFIHQDPASFSSKARKMDHFVRDFDARPNNIIYHGHVE
RKGEKPLGVEMKPELKKEKFFVEPVNLRVNFHKHGHDQKEYNN
>194.Vitis vinifera SST2
RKEPGVYWRDVMKDEPMPKAIQGLLPEDQSSSLLSKKPNCQTIPGARNGGDIVKGFEPKK
EKVFWVYDDEDAKLTEEKSFVKDLEPRTNVSA
