# Supplementary material for: A Comprehensive Analysis of Short Specific Tissue (SST) Proteins, a New Group of Proteins from PF10950 That May Give Rise to Cyclopeptide Alkaloids
Source: Plants (Basel). 2025 Apr 3;14(7):1117. doi: 10.3390/plants14071117 (PMC11991032; doi:10.3390/plants14071117)
Supplement: Supplementary file 1 [file plants-14-01117-s001.zip › List S4.pdf]

List S4. List of 151 SST mature protein sequences used in sequence comparison in FASTA format. The sequence numbers are the same as in Table S1. Please note that except in the WebLogo analysis of Figure S2A, the sequence number 109 from *Betula platyphylla* has been removed.

```
>1.Alyssum linifolium SST1
RSGGVAEEYWKKMMKNEPLPEPIKELLNNPFRNTQEIFIQNFDPKSVAIIYRSPKE
>2.Alyssum linifolium SST2
RPARGVAEEYWRKMMKNEPFPEPIKELLNNPFRTAQERFIPNFDTKVLVLIYHNPHHD
>3.Alyssum linifolium SST3
RPARGVAEEYWRKMMKNEPFPEPIKELLNNPFRTAQERFIPNFDTKVLVLIYHNPHHD
>4.Alyssum linifolium SST4
RSEGGVAEEYWKKMMKNEPLPEPIKELLNNPFRNTQEIFIQNFDPKSVAIIYRSPKE
>5.Arabidopsis halleri SST1
RTGGVAEEYWKKMMKNEPLPEPIKELLNNPFRTGQERFIQNFDTKSVVIIYHNPNE
>6.Arabidopsis lyrata SST1
RTGGVAEEYWKKMMKNEPLPEPIKELLNNPFRTGQERFIQNFDTKSVVIIYHNPNE
>7.Arabidopsis lyrata SST2
RTGGVAEEYWKKMMKNEPLPEPIKELLNNPFRTGQERFIQNFDTKSVVLIYHNPNE
>8.Arabidopsis thaliana SST1
RTGGVAEEYWKKMMKNEPLPEPIKELLNNPFRTAQERFIQNFDTKSVVIIYHNPNE
>9.Boechera stricta SST1
ESGRGVAEEYWKKMMKNEPLPEPIKELLSNPFRTAQEMFIQDFDTKSVVLLYRNPNK
>10.Boechera stricta SST2
RSGGGGVAEEYWKKMMKNEPLPEPIKELLNNPFRTAQERFIQSFDTKSVVIIYHRPNE
>11.Brassica oleracea SST1
RSGGVAEEYWKKIMKNEPLPEPIKELLNNPFRTGEERFVKDFKTKSIVIIYHNPNV
>12.Brassica oleracea SST2
KSEVAEDYWKKMMKSEPLPEPIKDILNNPFRTGQERFAKNFNTKSVVIIYHNPNV
>13.Brassica rapa SST1
RSGGVAEEYWKKMMKDEPLPEPIKDLLNNPFRTGQERFVKDFNTKSIVIIYHNPNV
>14.Brassica rapa SST2
RSGGVAEEYWKKIMKNEPLPEPIKELLNNPFRTGEERFVKDFKTKSIVIIYHNPNV
>15.Brassica rapa SST3
KSEVAEDYWKKMMKSEPLPEPIKDILNNPFRTGQERFAKNFNTKSVVIIYHNPNV
>16.Cakile maritima SST1
RSGGVAEEYWKKIMKNEPLPEPIKELLNNPFRTGEDRFVKDFKTKSIVIIYHNPNV
>17.Cakile maritima SST2
RSGGVAEEYWKKMMKNEPLPEPIKELLKNPFRTGEERFVKDFNTKLVVIIYHNPNV
>18.Cakile maritima SST3
KSGVAEEYWKKMMKNKPLPEPIKDFLNNPFRTGPERFVKNFNTKSVVIIYHNPNV
>19.Camelina sativa SST1
RSGGGGVAEEYWKKMMKNEPLPEPIKELLYNPFRTAEERFIQSFDTKSVVIIYHNPKE
>20.Capsella grandiflora SST1
RSGGGVAEQYWKKMMKNEPLPEPIKELLYNPFRTVDERFIQSFDTKSVVIIYHNPNE
>22.Capsella rubella SST1
RSGGGVAEQYWKKMMKNEPLPEPIKELLYNPFRTVDERFIQSFDTKSVVIIYHNPNE
>24.Caulanthus amplexicaulis SST1
RSGGVAEEYWKKMMKNEPLPEPIKELLNNPFRTGQERFVKNFNTKSIVIIYHNPNV
>25.Caulanthus amplexicaulis SST2
RSGGVAEEYWKKVMKNEPLPEPIKELLNNPFRIGQERFVKDFNTKSIVIIYHNPNV
>26.Cleome violacea SST1
RREAEDYWKKTMKDEPLPEPIKDLLNNPFRTDQKFIHNFDTKSLALIYHKN
>27.Cleome violacea SST2
RMEPEEYWKKAMAEAELPEPIKELMNNPFRSKHEKFINNFGTKATVLIYHNPNP
>28.Crambe hispanica SST1
RSGGVAEEYWKKIMKNEPLPEPIKELLNNPFRTGEERFVRDFKTKSIVIIYHNPNV
>29.Crambe hispanica SST2
KSGVAEEYWKKMMKNEPLPEPIKDFLNNPFRAGQERFAKNFNTKSIVIIYHNPNI
>30.Descurainia sophioides SST1
```

RSEGGVAEEYWKKMMKNEPLPEPIKELLNNPFRNTQEIFIQNFDPKSVAIIYRSPKE
>31.Descurainia sophioides SST2
RPARGVAEEYWRKMMKNEPFPEPIKELLNNPFRTAQERFIPNFDTKVLVLIYHNPHHD
>32.Diptychocarpus strictus SST1
RSGGGGVAEKYWKKVMKNEPLPEPIKELLNNPFRTAKERFIKNFNTKSVVIIYHNPNE
>33.Euclidium syriacum SST1
RPGEGVAEKYWKKVMKNEPLPEPIKELLNNPFRTAQERFIKNFKTKSIVIIYHNPNE
>34.Eutrema salsugineum SST1
RSGGVAEEYWKKVMKNEPLPEPIKELLNNPFRTAKERFVKNFNTKSIVIIYHNPNV
>35.Eruca vesicaria SST1
KSGVPEDYWKKMMKKEPLPEPITDILNNPFRKGQERFVKNFNTKSIVIIYHNPNA
>36.Eruca vesicaria SST2
RSGGVAEEYWKKIMKNEPLPEPIKELLNNPFRTGEDRFVKDFKTKSIVIIYHNPNV
>37.Iberis amara SST1
RSGGGVAEEYWKKMMKNDPLPEPIKELLNNPFRTEKERFITNFNTKSILIIYHDPLV
>38.Iberis amara SST2
RSGREVIEEYWKKMMKNEPLPEPIKQILNNPFRTAQEKFVTNFDTHSVLIIYHNPIE
>39.Isatis tinctoria SST1
RSGGVAEEYWKKMMKNEPLPEPIKELLNNPFRTGDERFVRDFNTKSIVIIYHNPNV
>40.Lepidium sativum SST1
RSGREVIEEYWKKTMKNEPLPEPIKVLLNNPFRTGDERFIQSFDTKSVVIIYHSPKE
>41.Lunaria annua SST1
RSGGGGVAEEYWKKMMKNEPLPEPIKELLHNPFRTEQEMFITNFDTKSVVIIYHNPNE
>42.Malcolmia maritima SST1
RPRGGVQEEYWKKMMKNEPLPEPIKELLYNPFRTGQGLFTKDFDTKSAVLIYHNPNE
>43.Malcolmia maritima SST2
RSGGVAEKYWKKIMKNEPLPEPIKELLYNPFRTGDERFIQSFDTKSVVIIYHTPNE
>44.Myagrum perfoliatum SST1
RSGGVAEEYWKKMMKNEPLPEPIKELLNNPFRTGEERFVKDFNTKSIVIIYHNPNV
>45.Raphanus sativus SST1
RSGGVAEEYWKKIMKNEPLPEPIKELLNNPFRTGEERFVKDFKTKSIVIIYHNPNV
>46.Rorippa islandica SST1
RPGGVAEEYWKKMMKNEPLPEPIKELLNNPFRTAQEKFIQNFDTKSVVIIYHNPKE
>47.Schrenkiella parvula SST1
RSGGVAEEYWKKMMKNEPLPEIIKELLNNPFRTGRERFVKNFNTKSIVIIYHKPNV
>48.Sinapis alba SST1
RSGGVAEEYWKKIMKNEPLPEPIKELLNNPFRTGEERFIKDFKTKSIVIIYHNPNV
>49.Sinapis alba SST2
RLGGVAEEYWKKMMKDEPLPEPIKDLLNNPFRTGQERFVKDFKTKSIVIIYHNPNV
>50.Sinapis alba SST3
KSGVAEEYWKKMMKSEPLPEPIQDLLNNPFRTGQERFVKNFNTKSIVIIYHNPNV
>51.Sinapis alba SST4
KSGVAEEYWKKMMKSEPLPEPIQDLLNNPFRTGQERFVKNFNTKSIVIIYHNPNV
>52.Stanleya pinnata SST1
RSGGVAEEYWKKMMKNEPLPEPIKDLLNNPFRTGQERFVKNFNTKSIVIIYHNPNV
>53.Stanleya pinnata SST2
RSGGVAEEYWKKVMKNEPLPEPIKELLNNPFRIGQERFVKDFNTKSIVIIYHNPNV
>54.Thlaspi arvense SST1
RSGGGVAEEYWKKMMKNEPLPEPIKELLDNPFRTARERFVKNFNTKSIVIIYHNPNV
>55.Arachis hipogea SST1
RKDQGEYWKDMMDKQTMPEAIKDLLVEDPQVSSSHARKNKDQFRKDFDIKPNVILYHSHV
GPKKQKA
>56.Arachis ipaensis SST1
RKELVGEYWKKMIKGQSMPEAIKEILVVEDPQISSSDSSRTKDNFIRDFDIKPNVILYHS
HKQKQKHNHKNPFLNNNLEEPEFQETENK
>57.Cajanus cajan SST1
RKDLGAYWKNMMKGQAMPQAIKDLVEASDAVDAGTKNRFVRDFDVKPNVILYHTHVKSSK
QKQKLFLNNNQD
>58.Cicer arietinum SST1
RKDLVDYWKNMMNDQPMPETIKELVQNPQVTDAGNDKFIRNFDIRPNVILYHTHVDSNKK
QHVFVNNSEQDHFHGITRKHG

>61.Glycine max SST1
RKDLGWYWKNVMKEQPMPQAIKDLVEDSQASAAGKKDRFIRDFDVKPNVILYHTHVVPMK
QKHKHKQNPFVKNQD
>62.Glycine max SST2
RKDLGGYWKNMMKEQPMPQAIKDLVEDSQASDTGKKDLFTRDFDVKPNVILYHTHVVSMK
QKQKPFLQN
>63.Glycine max SST3
RKDMGDYWKNMMNGQPMPEAIKDLLVQDPQVSDAMKDHFIRDFDIKPNVILYHTHVVPHK
HKQKIQQAMAKKLEPEFQGTERHG
>64.Glycine max SST4
RKDMGDYWKNMMNGQPMPEAIKDLLVQDPQVSDAVKDHFIRDFDIKPNTILYHTHVVPNK
QKQKQNIQQAMAKKLEPKFQGTERHG
>66.Glycine max SST6
RKDLGDYWKKMMNDEPMPEAIKDLIQDQQVQDATADHFIRYFDMKPNIILYHTHVVSKKQ
QQQKAFDHKFKPVSRNGKSWLNKP
>68.Glycine soja SST1
RKDLGGYWKNMMKEQPMPQAIKDLVEDSQASDTGKKDLFTRDFDVKPNVILYHTHVVSMK
QKQKPFLQN
>69.Glycine soja SST2
RKDLGWYWKNVMKEQPMPQAIKDLVEDSQASAAGKKDRFIRDFDVKPNVILYHTHVVPMK
QKHKHKQNPFVKNQD
>70.Glycine soja SST3
RKDMGDYWKNMMNGQPMPEAIKDLLVQDPQVSDAMKDHFIRDFDIKPNVILYHTHVVPHK
HKQKIQQAMAKKLEPEFQGTERHG
>71.Glycine soja SST4
RKDLGDYWKKMMNDEPMPEAIKDLIQDQQVQDATADHFIRYFDMKPNIILYHTHVVSKKQ
QQQKAFDHKFKPVSRNGKSWLNKP
>74.Glycine soja SST7
RKDMGDYWKNMMNGQPMPEAIKDLLVQDPQVSDAVKDHFIRDFDIKPNTILYHTHVVPNK
QKQKQNIQQAMAKKLEPKFQGTERHG
>75.Lotus japonicus SST1
RKDLGDYWKDMMNGQPMPEAIKDLLVEDPQVSDAAGKDHFTRDFDIRPNVILYHTHVESK
KQNQKQKKQLFVKNFQLPEFEGITGSHG
>76.Lotus japonicus SST2
RKDMGGYWKNVMNGQPMPEVVKDLIQDPHASDAGKDHFIRDFDIKPNNVSIFHTHVVPTS
RSRRHLTRNLN
>78.Lotus japonicus SST4
RKDMGEYWKNVMNDQPMPEVVKDLIEDPQVSDAGKDHFIRDFDIKPNNATIFHTHVVPTS
RSRRHLTRNLN
>79.Lupinus albus SST1
RKDVEDYWKNKMKGQPMPEPIKDLTYDLVAASDAGKSRFIRDFDIKPNVILYHTHVVSKE
QKQKNPFVKKIETKFKKPEVMVVEQTVKKD
>80.Lupinus albus SST2
IKHEGEYWKNIMKDQPMPETIKDLLVQDPQVLDSGKDHFIRDFDIRPNVILYHTHVVSKK
QKQHPFVNNFEPEFQE
>81.Lupinus angustifolia SST1
RQHQGEYWKNIMKDQPMPETIKDLLVQDPQAYTEKYHFIRDFDIRPNVILYHTHVVSKKQ
KQHPFVKNFEPEFQEIGTRV
>82.Medicago truncatula SST1
RKDLGDLWKNKMNKQPMPEAIKNFIQVPKALGEGKEDHSFTTDFDVNPNIILYHTHVHQD
EKPFEHAARKMESLLPKRG
>84.Medicago truncatula SST3
RKDLGGYWKNMMNDQPMPEAIKELVQNQEVSDDFIRDFDVKPNVILYHTHVESKKKKEHV
FVKNSQQELHGT
>85.Medicago truncatula SST4
RKDLGDYWKNKMNEQPMPEAIKNLIQVPKALDEGKEDHSFTTDFDVNPNIILYHTHVHQD
EKPFQHAARKMEPLLPKRG
>86.Phaseolus acutifolius SST1
RKDMRGYWKDMMKEQPMPQAIKDLIEDSEVSEAGKGRFVRDFDVKPNVILYHTHVVPMKQ
RQKNQD
>87.Phaseolus acutifolius SST2

RKDMGDYWKNMMNGQPMPEAIKDLLVQDPQVSDTVKDHFIRDFDIRPNVILYHTHIPPNK
RKQHAMAKKIEEFHGTGN
>89.Phaseolus lunatus SST1
RKDMRGYWKDMMKEQPMPQAIKDLIEDSEASEAGKGRFVRDFDVKPNVILYHTHVVPRKQ
RQKKQD
>90.Phaseolus lunatus SST2
RKDMGDYWKNMMNGQPMPEAIKDLLVQDPQVSDTVKDHFIRDFDIRPNVILYHTHIPPNK
RKQHAMAKKIKEFHGTGRHA
>92.Phaseolus vulgaris SST2
RKDMGDYWKNMMNGQPMPEAIKDLLVQDPQVSDTVKDHFIRDFDIRPNVILYHTHIPPNK
RKQHAMAKKIEEFHG
>93.Trifolium pratense SST1
RKDLDDFWKNMMNDQPMPEAIKELVQNTKVIDSRKDNFIRDFDVKPNLILYHTHVESKKQ
KRKQHIFVKKSEQEEFHGTQKHG
>94.Trifolium pratense SST2
RKDPGEYWKNMMKGEAMPEAIKELIQDPQAIYAGKDGFMRDFDVKPNAILYHTHVMSMEQ
TRRC
>95.Trifolium pratense SST3
RKDLGDYWKNKMNEQPMPEAIKNLIQVPKALSSNEEKDNSFNRDFDVHPNVILYHTHVHE
EKKPFDESAVRKMESLLPNKG
>96.Vigna angularis SST1
RKYMGDYWKNMMNGQPMPEAIKDLLVQDPQESDAAAVKDHFIRDFDIRPNIILYHTHDMS
RKQKQHAMAKKIEELQEPGRHG
>97.Vigna unguiculata SST1
RKDLDGYWKETMKEQPMPEAIKDLIEDSQVSETGKDRFIRDFDVKPNVILYHTHVVSMKQ
TQKNQD
>99.Amaranthus hypocondriacus SST2
RREPGEYENDMMMKEKVIPEAIKDFILYDKLYDAHKLAQAIKDISEDFDSTPNATIYHDD
ADQE
>100.Anacardium occidentale SST1
RKEPGDYWKSLMKEQPMPKAIKDLLHQDSEGRKIDHFAKDFDVNPNVIIYHSHSEPKQER
QEEKSLVNQIKSQRG
>102.Cynara cardunculus SST1
RKGPEEYWRSIMKDEPMPKAIQDVLSEDSTDKENNRDRFTRDFDTKPNLIIYHSHVMYNQ
KDHELASSKIN
>103.Helianthus annuus SST3
RMGPEEYWRSVMKDEPMPETLQNVLVHDSSSLEDKEKKKDRFIRNFDTKPNLIIYHSHVM
YNQKGHELASSKLN
>104.Helianthus annuus SST2
RSDPREYWRSVMKDEPMPKTIQDVLPLEDGMKVNKDIFTRNFNLKPNLIIYRSHVVYSEK
NHHIVSSSSSSFDELN
>105.Helianthus annuus SST3
RKDPEEIWRSVMKDEPMPKTIQDALSQDSTRSNKEVNMKDQSVSVFDTQPNHKMFQRDFD
TKPNSMTFLYPKPTL
>106.Helianthus annuus SST4
RKDPKDWRNAMKYEPIPPDALSQDSTTLNDKENNKDQFVRDFDKQPNLKMFAKGFNPKPS
SLVSNGCPPKQD
>107.Lactuca sativa SST1
REGPKEYWRSVMKDEPMPKAIQDVLIQDSARSNNNKDRFTRNFDTKPNLIIYHSHVIYNQ
KDHELASSKMN
>109.Betula platyphylla SST1
RKDLGGYWKNVMDSQPMPEAIQGRIYPQTGKGVHFAKDFDPVSLVDSNIVWVPPPGDHLI
HPEARKEDHLPRSSSRPKAENSPISKAKP
>110.Handroanthus impetiginosus SST1
RKDPGDYWKSIMNGEPMPKAITDLIHRYNLESDSNMKMDYFIKNFNTKANVIIYHSHHKV
HSDHKPKVSNMKLV
>112.Carica papaya SST1
RKEAGGEYWGSVMKGQAMPEVVEALTYPNSPTTTQPQQTQPKRESRNFEDAWR
>113.Beta vulgaris SST1
RKNPEEYWKKVMKDQPIPEVINGIIDEEIMAKSYEKETFWKHFKRDFDVNSNVIIYHSHQ
ENNHLSPST

>114.Chenopodium quinoa SST1
RNSPEDYWKKVMKDQPIPEAINGIIDQKMANASEKNSLWSHFKRDFDVTSNVIIYHPHQV
NNHRSPSD
>115.Spinacea oleracea SST1
RNSPEDYWKKIMKDQPIPEAIGGIIDQEMANPSEKKPFWSHFKRDFDVTSNVIIYHPHQE
NTMRSPSA
>116.Ipomoea nil SST1
RRDPGEYWDAMMNGDPMPKAITDLLLINQDPSSSSSSPNDRFIRDFDTKPNLIIYHSHVD
VYPKKHEVVAKDVQQKKT
>118.Kalanchoe fedtschenkoi SST2
RKDSGDYWKGVMKDEPMPAALEALILPIPNTSSSQSSLVKKPDCRHEDPTASHVKGKTFG
YDIEPRPSVTVRPQVQQRIK
>122.Cucurbita maxima SST1
RKEKGEYWKKVMKDEAIPEMLKELLFDDDSLVSSDAQSERFMNNFDTHPNAIIYHSHGAT
HDHPGHKTKLTAP
>124.Vaccinium darrowii SST2
RKDPIAADYWKSMMRGSPIPRAIEEVLVHDQDGETSVSKQKEKKLWDPSQTNMAHFRRDF
ETTHNVLIYHSHNNMGHGTTP
>125.Vaccinium darrowii SST3
RKDPADYWKSIMKGEPMPEKIQRLVFHQDPSSLSKEKIKKWCSSSAIINMEHFRKDFDTS
PNLIIYHSGAEPRN
>127.Vaccinium darrowii SST5
RKDPIAADYWKSMMKGKPMPKAIEEVLVHDHDGETSVSKQKEKKMLDPSQINMAHFKMDF
ETTRNELIYHSLNKP
>130.Manihot sculenta SST2
RKVPEDYWKVVMKDQPIPEAIKNLFVEEDEEAAASANKKNHFVSDFDTRAVAVIYRSHGD
INKKMNMLR
>133.Castanea dentata SST1
RKDTRDYWKSIMKDQPIPEAIKELFHRDPPYLFDATKKDHFVLDFDARPNAIIYHAKEEK
PNIKDFEPNYHGSELKEE
>135.Quercus rubra SST2
RKDTGDYWKSIMKDQPIPEAIKELFHQDPPYLFDATKKDHFVLDFDARPNAIIYHAKEEK
PNVKDFEPNYHGSELKEE
>136.Quercus suber SST1
RKDTGNYWKSIMKDQPIPEAIKELFHQDPPYLFDATKKDHFVLDFDARPNAIIYHAKEEK
PNVKDFEPNYHGSELKEE
>137.Dorcoceras hygrometricum SST1
RKDGGNYWKSKMKGEPMPKAIQDLFNQNTSSEMRTTDRFVRNFEAKRTFIVYHSPTGVHP
DEP
>138.Hydrangea quercifolia SST1
RTDPGEYWKNIMKGETMPKAIQDLLHHQDLTGKGIDKELFIKDFDTKTSGIIYHSHVEPK
EVKTFFKHVTEPEAEKSANLVKPKANGY
>139.Carya illinoinensis SST1
RKDPEDYWKSIMKDQPIPEAIKGLLRRDLPYASDAREKDHFLKDFDVTPNAIIYHAHVED
KKEKKPCVEDFEQKSYTELNLFEG
>140.Juglans regia SST1
RRDPGDYWKSIMKDQPIPEAIKGLLHRDLPYASDARENDHFLKDFDVTPNAIIYHAHVED
KDEKKPACVEDFEQKSHTELNLIQG
>144.Gossypium arboreum SST1
RKEPGEYWRSVMKDQRMPEAIKGLLHEDETGSGSGAEMKMKQFVKDFDSRHSLIIYHNSP
ESKQEDTTHAKDVKHTKDQKQDKSDRKN
>145.Gossypium barbadense SST1
RKEPGEYWRSVMKDQPMPEAIKGLLHEDETGSGSGAEMKMKQFVKDFDSRHSLIIYHNSP
ESKQEDTTHAKDVKHTKDQKQDKSDRKN
>146.Gossypium darwinii SST1
RKEPGEYWRSVMKDQPMPEAIKGLLHEDETGSGSGAEMKMKQFVKDFDSRHSLIIYHNSP
ESKQEDTTHAKDVKHTKDQKQDKSDRKN
>147.Gossypium hirsutum SST1
RKEPGEYWRSVMKDQPMPEAIKGLLHEDETGSGSGAEMKMKQFVKDFDSRHSLIIYHNSP
ESKQEDTTHAKDVKHTKDQKQDKPDRKN
>148.Gossypium mustelinum SST1

RKEPGEYWRSVMKDQPMPEAIKGLLHEDETGSGSGAEMKMKQFVKDFDSRHSLIIYHNSP
ESKQEDTTHAKDVKHTKDQKQDKSDRKN
>149.Gossypium raimondii SST1
RKEPRDYWKSVMKDQPIPEAIQGLLHQDEASAMDSENFVKDFDSRHSFIIYHSNLKHKEE
EDKTYVKDLKNQKEHKSDKKNQTEKY
>150.Gossypium raimondii SST2
RKEPGEYWRSVMKDQPMPEAIKGLLHEDETGSGSGAEMKMKQFVKDFDSRHSLIIYHNSP
ESKQEDTTHAKDVKHTKDQKQDKPERKN
>151.Gossypium tomentosum SST1
RKEPGEYWRSVMKDQPMPEAIKGLLHEDETGSGSGAEMKMKQFVKDFDSRHSLIIYHNSP
ESKQEDTTHAKDVKHTKDQKQDKPDRKN
>152.Gossypium tomentosum SST2
RKEPRDYWKSVMKEQPIPEAIQGLLHQDEASAMDSENFVKDFDSRHSFIIYHSNLKHKEE
EDKTYVKDLKNQKQHKSEKKNQTEKP
>153.Morus notabilis SST1
RKDAGEYWNSIMKDQPIPEAIRDLFYDQDLPSDLTGPTKHDRFVRDFDVQPNVIIYHSHA
QPQGEDNHKPSVHNHHHEELEETH
>154.Corymbia citriodora SST1
RKSPGDYWKKIMKDQPMPEPIRDLIRLRNEENAIKFVRDFDTGPNVIIYHSHGDAKEKKH
CRENVEEGEDERLKMRDDQDQKEMTSA
>155.Eucalyptus grandis SST1
RKSPGDYWKKIMKDQPMPESIRDLIRPGNEKNTNDFMRDFDMRPNVIIYHSHGDAKEKKH
CGENAEKGEDERLKMPDNQDQKKMTSA
>156.Olea europaea SST1
RKDPGDYWKSMMNDEPMPKAITDLIHHDTAEESNHFLRNFDTKPNVIIYHSHVQSAKMKP
SPLEG
>157.Sesamum indicum SST1
RKEPGAYWKSVMNGDEMPKAITDLLHHDHQSSSATDLNTERDRFIRNFDTKANVIIYHSH
DDHHHDHNSKDQRRPMWMV
>158.Portulaca amilis SST1
RIGPEEYWKRVMKDEKLPEAITSLLNQDSADPLSQENLFHGYFKREFYVDNPDNAIIYHP
NHHDTQIPTWSAKRLDSALGKLDPQ
>159.Ziziphus jujuba SST1
RKDIGDYWKSIMKEQPMPEAIRDLFHQEDVPSLPGSRKMDRFARDFDIRPNLIIYHAHPK
PVEGKTETEDEEEFKVIKQINRG
>160.Fragaria vesca SST1
RKDMGDYWKSVMNDQPMPEALKDLFSHQDEDVPSFSASKNKDHRFVRDFDIRPNVIIYHS
AHHHHADHQPEEMMHEMQPKAYIQTVNHG
>161.Malus domestica SST1
RKDSGGYWKSVMNDQPMPEAIKGLFVHHEQEDQVPSKEKSHFVRDFDMRPNVIIYHGAHH
HHQDQPAEKKPFFQETSYIQTVNHG
>171.Poncirus trifoliata SST2
RKDLEEYWRIVMKGQPMPESIQDLLAVDRAASNTNENADQKSNPLILSDNAQPSEVESLA
KNFNPRPNVLAYSFCW
>172.Populus deltoides SST1
RKEPREYYWKSMTKDQPMPEAIKDLFVRDPAGAGKLNHFVKDFDTRHSAIIYHSRDGKDE
LKETNPTNARDHEEDKAHAP
>173.Populus deltoides SST2
RKEPRENYWKSMTKDQPIPGAIRDLFVQDPAAGADKMNHFVKDFDTKHNAIIYHSHEKDK
LKEKKSMNPTNTWDHEKEKE
>174.Populus euphratica SST1
RKEPREYYWKSMMKDQPMPEAIKDLFVQDPAGAGKLGHFVKDFDTRHSAIIYHSHDEKDE
LKETNPTNARDHEEDKAYAP
>176.Populus trichocarpa SST2
RKEPRENYWKSMTKDQPIPGAIRDLFVQDPAAGADKMNHFVKDFDTKHNAIIYHSHEKDK
LKEKKSMNPTNTWDHEKEKE
>177.Salix purpurea SST1
ARKEPREYYWKSMMKDEPMPEAIKELFVEDPAGAGKMSHFVKDFDTRHSAIIYHSHAEKD
RLKERKSTNARDHDGDAQ
>178.Salix purpurea SST2
RKEASEYYWKSMTKDQPIPEAIRDLLVRDPAGSDKMNHFVKDFDTKHSAIIYHSPEKDKL

KEKNP
>179.Lindenbergia philippensis SST1
RNNPGENYWKKLMGKEPIPDAIKGLFSTVDSSHKFVKDFETKENVIIYHKHFDVAGKNKK
PSVGK
>180.Mimulus guttatus SST1
RPSPGEYWKSRMNGEAMPKALMDVVSDSDAKTSRFVKDFNTKPNVIIYHSNYHANKHAQA
PKPC
>181.Capsicum annun SST1
DGPEEYWNSKMNGDPMPKALRGLLNDQYQNFPTERNKRSHKFLRDFDMKANIIIYHNDVD
IYPKRPRPTAEEAERRKTVDP
>182.Nicotiana attenuata SST1
TSDPEEYWKSVMNGDPMPKALTDLLHNQYQDFPVERNKDRFLRDFDLKPNIIIYHNDVDI
YPKRPRPTATDDNFSDTKEAERKEPVNPEN
>183.Nicotiana sylvestris SST1
RSDPEEYWKSVMNGDPMPKALTDLLHNQYQDFPVERNKDRFLRDFDLKPNIIIYHNDVDI
YPKRPRPTAKDDIFSENKEAERREPVNPGN
>184.Nicotiana tabacum SST1
RSDPEEYWKSVMNGDPMPKALTDLLHNQYPDFPVERNKDRFLRDFDLKPNIIIYHNDVDI
YPKRPRPTAKDDIFSENKEAERREPVNPGN
>185.Solanum lycopersicum SST1
DGPEEYWKSKMNGDPMPKALKELLNDQYQDFPIERNKFVRNFDLKANIIIYHNDVDIYPK
RSRPTP
>186.Solanum pennelli SST1
DGPEEYWKSKMNGDPMPKALKELLNDQYQDFPIERNKFVRNFDLKANIIIYHNDVDIYPK
RSRPTP
>187.Solanum tuberosum SST1
DGPEEYWKSKMNGDPMPKALKDLLNDQYQDFPIERNKFVRNFDLKANIIIYHNDVDIYPK
RSRPTP
>188.Solanum tuberosum SST2
RKDPGEYWRDVMKDEPMPKAIQHLMPQPDKEKIDSHKSSFEPIPNASSFIE
>189.Herrania umbratica SST1
RKEPGDYWKSVMKDQPMPEAIKGLLHQDPASALGSEKNMKHFVTDFDTRHSAIIYHSGPQ
SKVEDNPQVKDLKDQKQQKSDKKN
>190.Theobroma cacao SST1
RKEPGDYWKSVMKDQPMPEAIKGLLHQDPASALGSEKNMKHFVKDFDTKHSVIIYDSGPQ
SKVEDNPHVKDLKDQKQQKSDKKN
>191.Corchorus olitorius SST1
RKEPGEYYWKSVMKDQPMPEAIKGLFHDQDPASSSSALGSDKKMNTFVKDFDSRHSVIIY
HTSPVSEKEESKHSVKDLKP
