# Supplementary figures and images for: A Comprehensive Analysis of Short Specific Tissue (SST) Proteins, a New Group of Proteins from PF10950 That May Give Rise to Cyclopeptide Alkaloids
Source: Plants (Basel). 2025 Apr 3;14(7):1117. doi: 10.3390/plants14071117 (PMC11991032; doi:10.3390/plants14071117)

**B**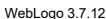

**C**

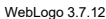

D

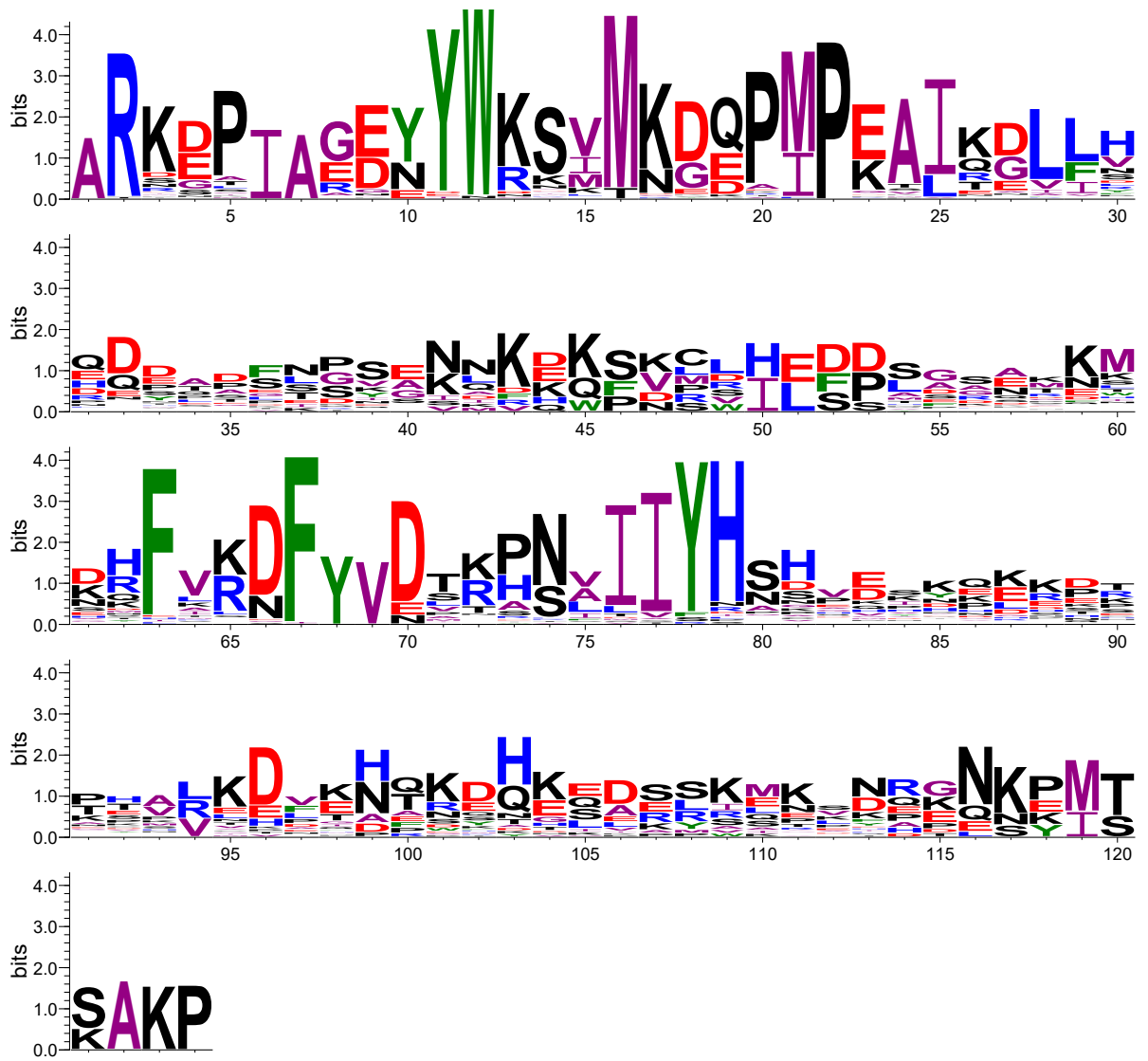

Supplement: Supplementary file 1 [file plants-14-01117-s001.zip › Figure S2.pdf]
